# Prophylactic endoscopic transpapillary gallbladder stenting at fully covered self-expandable metal stent placement to prevent cholecystitis in malignant distal biliary obstruction: A multicenter prospective single-arm study protocol (PROTECT study)

**DOI:** 10.1371/journal.pone.0361468

**Published:** 2026-10-29

**Authors:** Takayuki Oda, Yusuke Kurita, Masato Suzuki, Satoru Shinoda, Takamitsu Sato, Shinichi Nihei, Yu Honda, Takeshi Iizuka, Shin Yagi, Sho Hasegawa, Kensuke Kubota, Masato Yoneda

**Affiliations:** 1 Department of Gastroenterology and Hepatology, Yokohama City University Hospital, Yokohama, Japan; 2 Department of Gastroenterology, Yokohama Rosai Hospital, Yokohama, Japan; 3 Department of Biostatistics, Yokohama City University School of Medicine, Yokohama, Japan; 4 Department of Gastroenterology, Chigasaki Municipal Hospital, Chigasaki, Japan; 5 Department of Gastroenterology, National Hospital Organization Yokohama Medical Center, Yokohama, Japan; Athens Medical Group, Psychiko Clinic, GREECE

## Abstract

**Background:**

Malignant distal biliary obstruction (MDBO) is commonly managed with endoscopic retrograde cholangiopancreatography (ERCP)-guided transpapillary stenting. Fully covered self-expandable metal stents (FCSEMSs) provide durable biliary drainage but may increase the risk of acute cholecystitis due to cystic duct obstruction. Prophylactic endoscopic transpapillary gallbladder stenting (ETGBS) at the time of FCSEMS placement has been proposed to reduce the risk of cholecystitis after FCSEMS placement. However, the available evidence is limited. This study aims to explore the potential clinical benefit of attempting prophylactic ETGBS by estimating the incidence of acute cholecystitis after FCSEMS placement and characterizing the technical feasibility and safety of the strategy in patients with MDBO.

**Methods:**

This multicenter, prospective, single-arm exploratory study (PROTECT study) will be conducted at four institutions in Japan. In all eligible patients undergoing FCSEMS placement for MDBO, prophylactic ETGBS will be attempted. The primary endpoint is the incidence of acute cholecystitis within 30 days after FCSEMS placement. Secondary endpoints include the technical success rate of ETGBS, procedure-related adverse events, the incidence of acute cholecystitis within 30 days among patients with technically successful ETGBS, and the cumulative incidence of acute cholecystitis from day 31 until the end of follow-up. The target enrollment is 50 patients. The primary endpoint will be summarized as a proportion with a two-sided 90% Wilson score confidence interval. A two-sided 95% Wilson score confidence interval will also be reported as a supplementary analysis, and a supplementary exploratory two-sided one-sample score test will compare the observed proportion with the historical benchmark of 12.1%.

**Discussion:**

This study will provide prospective data on the potential clinical utility, technical feasibility, and safety of attempting prophylactic ETGBS and may inform the design of future comparative studies.

**Trial registration:**

Japan Registry of Clinical Trials number: jRCT1030250361. Registered on 12 September 2025.

## Introduction

Malignant distal biliary obstruction (MDBO) can cause obstructive jaundice and may lead to hepatic dysfunction and cholangitis. Therefore, prompt biliary drainage is required, and endoscopic retrograde cholangiopancreatography (ERCP)-guided transpapillary stenting is widely accepted as the standard treatment [1–3]. Self-expandable metal stents (SEMSs) provide longer patency than plastic stents and are recommended for palliative drainage of MDBO [1,4–6]. Furthermore, in the preoperative setting, SEMSs have been reported to reduce the incidence of recurrent biliary obstruction and to minimize delays in neoadjuvant therapy compared with plastic stents [7–10].

SEMSs are categorized into uncovered and covered types. Fully covered SEMSs (FCSEMSs) can prevent tumor ingrowth and are removable when necessary, and are commonly used for MDBO [11–15]. Acute cholecystitis is a clinically important complication after FCSEMS placement and is thought to be associated with obstruction of the cystic duct orifice by the FCSEMS [16,17]. When acute cholecystitis occurs, additional invasive management—such as percutaneous transhepatic gallbladder drainage (PTGBD), endoscopic ultrasound-guided gallbladder drainage (EUS-GBD) or cholecystectomy—may be required, and planned surgery or chemotherapy may be delayed, potentially compromising the patient’s prognosis.

Prophylactic endoscopic transpapillary gallbladder stenting (ETGBS) at the time of FCSEMS placement may prevent obstruction of the cystic duct orifice and reduce the risk of acute cholecystitis after FCSEMS placement. However, evidence supporting this strategy remains limited to single-center retrospective studies [18–20]. Although these studies suggest that the incidence of post-procedural cholecystitis may be lower when ETGBS is technically successful, the reported technical success rate of ETGBS is 57.9%–75%, and ETGBS-related complications, such as guidewire perforation of the cystic duct, have also been described [18–20]. In addition, attempting ETGBS may prolong procedure time and increase procedure-related adverse events. Consequently, it remains uncertain whether ETGBS should be routinely attempted at the time of FCSEMS placement for MDBO.

The aim of this study is to explore the potential clinical benefit of attempting prophylactic ETGBS by estimating the incidence of acute cholecystitis after FCSEMS placement and characterizing the technical feasibility and safety of the strategy in patients with MDBO, thereby providing preliminary data to inform the design of future comparative studies.

## Materials and methods

### Study design and setting

This is a multicenter, prospective, single-arm exploratory study conducted at four institutions: Yokohama City University Hospital, Yokohama Rosai Hospital, Chigasaki Municipal Hospital, and National Hospital Organization Yokohama Medical Center. This study is referred to as the PROTECT study (PROphylactic gallbladder stenting To prEvent CholecysTitis). Patients who meet the eligibility criteria will be informed about the study using a written participant information document. Written informed consent will be obtained by the investigator at each participating institution before enrollment. In all enrolled patients, prophylactic ETGBS will be attempted at the time of FCSEMS placement for MDBO. The SPIRIT schedule of enrollment, interventions, and assessments is shown in Fig 1. Participant recruitment is ongoing and has not been completed. The first participant was enrolled on October 15, 2025. The SPIRIT checklist is provided in S1 File. The study protocol, version 1.3, is provided in S2 File (original Japanese-language version) and S3 File (English translation).

**Fig 1:**
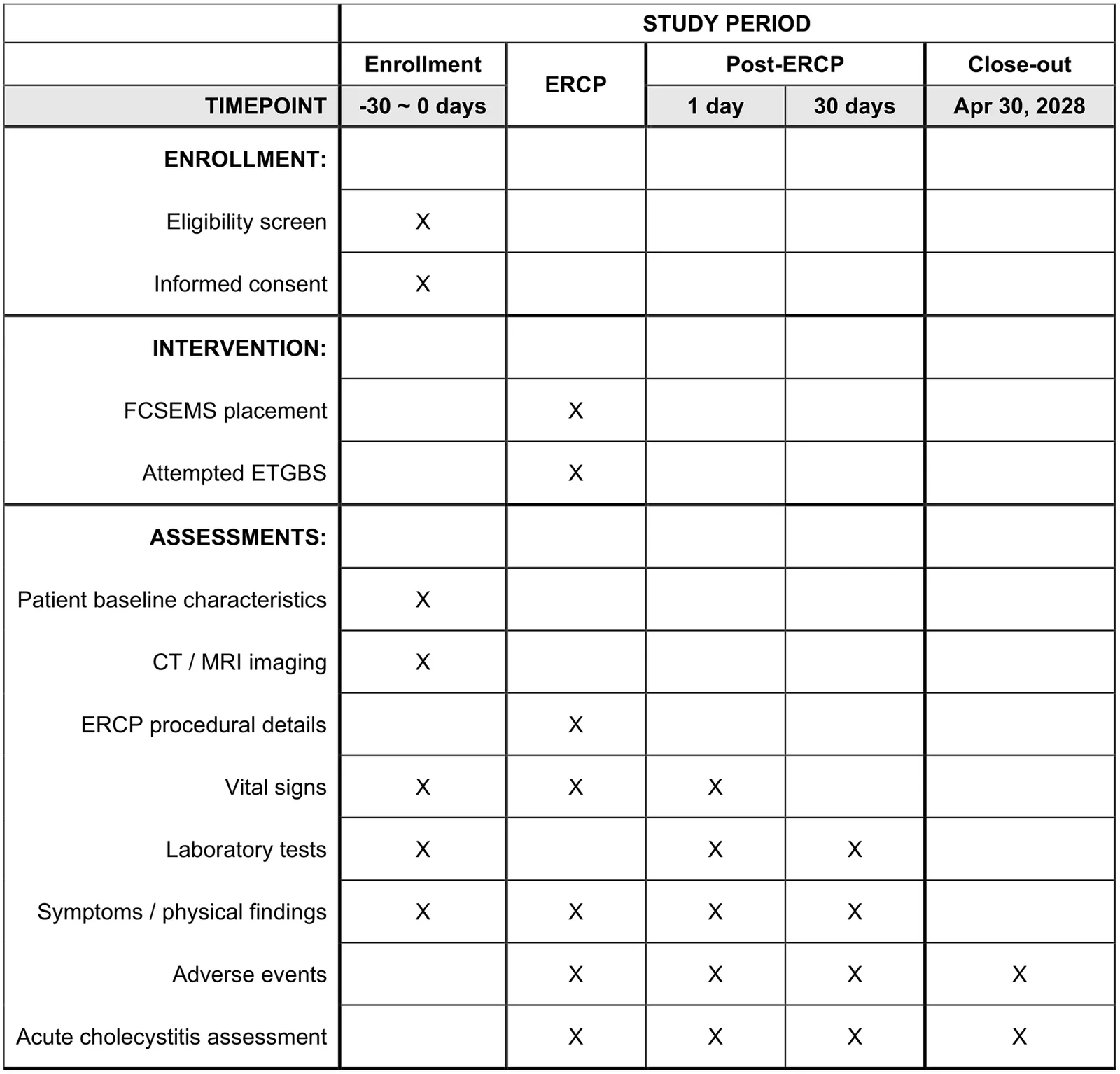
The SPIRIT schedule of enrollment, interventions, and assessments. CT, computed tomography; ETGBS, endoscopic transpapillary gallbladder stenting; ERCP, endoscopic retrograde cholangiopancreatography; FCSEMS, fully covered self-expandable metal stent; MRI, magnetic resonance imaging.

### Inclusion criteria

Eligible patients must meet all of the following criteria:

1 Age ≥ 18 years at enrollment;
2 Obstructive jaundice due to MDBO of any malignant etiology;
3 No history of cholecystectomy;
4 Expected survival of at least 4 months, as assessed by the site principal investigator based on the type and extent of the underlying malignancy, performance status, organ function, and planned anticancer treatment;
5 Written informed consent obtained from the participant.*

*If the participant is objectively assessed as lacking capacity to provide informed consent, written consent may be obtained from a legally authorized representative, limited to the participant’s spouse, a relative within the second degree of kinship, or a cohabiting family member aged ≥18 years.

### Exclusion criteria

Patients meeting any of the following criteria will be excluded:

1 Gallbladder drainage already in place at enrollment (PTGBD, EUS-GBD, or ETGBS);
2 Surgically altered anatomy other than Billroth I reconstruction that makes ERCP with a side-viewing duodenoscope difficult;
3 Patients will be excluded only when both of the following conditions are met on pre-ERCP CT and/or MRI: (i) the cystic duct orifice is clearly identifiable, and (ii) the proximal end of the planned FCSEMS is expected to remain distal to the cystic duct orifice without overlapping it. If either condition cannot be determined, the patient will not be excluded on the basis of this criterion. No fixed cutoff will be applied for the distance between the proximal margin of the malignant biliary stricture and the cystic duct orifice. The imaging assessment will be performed independently by two experienced endoscopists at each participating institution, and exclusion will require agreement between both endoscopists;
4 Duodenal obstruction due to tumor invasion that precludes ERCP;
5 Upper gastrointestinal perforation contraindicating ERCP;
6 Pregnancy, suspected pregnancy, or intention to become pregnant during the study period;
7 Follow-up at 30 days after FCSEMS placement is anticipated to be difficult;
8 Concurrent participation in another clinical trial of a drug or medical device, or participation in such a trial within 30 days before enrollment;
9 Any other condition that, in the opinion of the site principal investigator, would make participation unsafe or compromise the interpretation of the study results.

### Discontinuation criteria

Participants will be withdrawn from the study if any of the following applies:

1 Withdrawal of consent by the participant (or legally authorized representative);
2 Post-enrollment confirmation that the participant does not meet the eligibility criteria or meets any exclusion criterion;
3 Progression of the underlying disease after enrollment that renders FCSEMS placement difficult or infeasible;
4 Development of acute cholecystitis after enrollment but before FCSEMS placement;
5 Occurrence of a major protocol deviation;
6 Death;
7 Inability to continue follow-up for reasons such as transfer to another hospital;
8 Confirmation of pregnancy;
9 Any other situation in which the principal investigator judges that continued participation in the study is inappropriate.

Whenever possible, participants who discontinue the intervention or are withdrawn from the study will be followed according to the study schedule, and available data will be analyzed in the prespecified analysis set.

### Procedural details

#### ERCP and ETGBS procedures.

All ERCP procedures will be performed with the patient in the prone position under sedation with either midazolam or propofol, according to each institution’s protocol. A side-viewing duodenoscope (JF-260V, TJF-260V, or TJF-Q290V; Olympus Medical Systems, Tokyo, Japan) will be used. After the scope is advanced to the duodenum, a 0.025-inch guidewire (VisiGlide2; Olympus Medical Systems, Tokyo, Japan; Fielder, Asahi Intecc, Aichi, Japan; or equivalent) will be inserted transpapillarily into the bile duct. A catheter (MTW catheter; MTW Endoskopie, Wesel, Germany) will then be advanced over the guidewire, and cholangiography will be performed to confirm the distal biliary stricture and attempt to identify the cystic duct orifice. In patients with a naïve papilla, endoscopic sphincterotomy will be performed using a sphincterotome (CleverCut3; Olympus Medical Systems, Tokyo, Japan; ENGETSU, Kaneka Medix, Osaka, Japan; or equivalent).

After cholangiography, the endoscopist will intentionally manipulate a guidewire toward the visualized or presumed location of the cystic duct orifice with the aim of advancing it through the cystic duct into the gallbladder. Initiation of this intentional guidewire manipulation will constitute an ETGBS attempt, regardless of whether the cystic duct orifice is visualized or guidewire cannulation of the cystic duct is successful. Visualization of the cystic duct orifice without intentional guidewire manipulation will not be considered an ETGBS attempt.

If the guidewire is successfully advanced into the gallbladder, ETGBS will be performed using either a 7-Fr Through & Pass double-pigtail stent or a 5-Fr IYO stent (Gadelius Medical, Tokyo, Japan). The type of stent will be selected by the endoscopist on the basis of cholangiographic assessment of the course and diameter of the cystic duct. Regardless of the technical success of ETGBS, a 10-mm FCSEMS (HANAROSTENT; M.I. Tech, Seoul, Republic of Korea; or BONASTENT, Standard Sci. Tech Inc, Seoul, Republic of Korea) will be placed across the distal biliary stricture.

#### Discontinuation criteria and procedure times.

The ETGBS attempt will be discontinued if ETGBS is not successfully completed within 60 minutes after scope insertion. The attempt may be discontinued earlier if the endoscopist considers further manipulation technically difficult or unsafe. The 60-minute limit refers to the total elapsed time from scope insertion and includes standard ERCP steps performed before the ETGBS attempt, such as biliary cannulation, cholangiography, and endoscopic sphincterotomy when required. It does not represent 60 minutes of ETGBS-specific guidewire manipulation.

Total procedure time will be measured from scope insertion to scope withdrawal. ETGBS attempt time will be measured from the initiation of intentional guidewire manipulation toward the visualized or presumed location of the cystic duct orifice to either successful placement of the gallbladder stent or discontinuation of the ETGBS attempt. FCSEMS placement time will be measured from the initiation of advancement of the FCSEMS delivery system over the biliary guidewire to completion of FCSEMS deployment.

#### Procedural data and post-procedure management.

The manufacturer and model of all guidewires and catheters used during the ETGBS attempt will be recorded for each procedure. The type and caliber of the gallbladder stent placed and the FCSEMS model used will also be recorded. All procedures will be performed during hospitalization. Both operators and assistants will be endoscopists who have performed at least 200 ERCP procedures as the primary operator [21].

If acute cholecystitis develops during follow-up, management will be determined at each institution in accordance with Tokyo Guidelines 2018 (TG18) [22] and the patient’s clinical condition. No standardized rescue-treatment algorithm or predefined priority among interventions will be mandated across the participating institutions. All treatments provided for acute cholecystitis, including rescue interventions, will be recorded.

### Primary endpoint

The primary endpoint is the proportion of participants who develop definite acute cholecystitis within 30 days after FCSEMS placement among those in whom ETGBS is attempted. An ETGBS attempt is defined as the initiation of intentional guidewire manipulation toward the visualized or presumed location of the cystic duct orifice with the aim of advancing the guidewire through the cystic duct into the gallbladder.

Acute cholecystitis will be defined as definite acute cholecystitis according to TG18; cases classified as suspected acute cholecystitis will not be counted as events. Definite acute cholecystitis occurring within 30 days after FCSEMS placement will be counted as a primary endpoint event. Cholecystectomy or gallbladder drainage performed for definite acute cholecystitis will also be counted as an event.

Participants who die without preceding definite acute cholecystitis or who undergo cholecystectomy or gallbladder drainage for an indication other than definite acute cholecystitis within 30 days will remain in the denominator and will not be counted as having the primary endpoint. The number of such participants, the timing and causes of death, and the timing and indications for these interventions will be reported separately. The severity of every definite acute cholecystitis event occurring during follow-up will be graded as Grade I (mild), Grade II (moderate), or Grade III (severe) according to TG18.

### Secondary endpoints

The secondary endpoints are as follows: (1) the technical success rate of ETGBS; (2) the proportion of patients who experience endoscopic procedure-related adverse events; (3) the proportion of patients with technically successful ETGBS who develop acute cholecystitis within 30 days after FCSEMS placement; and (4) the cumulative incidence of definite acute cholecystitis from day 31 after FCSEMS placement until the end of follow-up, accounting for competing events. Technical success of ETGBS will be defined as successful placement of a plastic stent in the gallbladder. Endoscopic procedure-related adverse events and their severity will be defined according to the American Society for Gastrointestinal Endoscopy lexicon [23]. The diagnosis and severity grading of acute cholecystitis will be performed as described for the primary endpoint according to TG18. Among patients with technically successful ETGBS, the incidence of acute cholecystitis will also be summarized separately according to the type and caliber of the plastic stent placed (7-Fr Through & Pass double-pigtail stent or 5-Fr IYO stent). The overall study flow is shown in Fig 2.

**Fig 2:**
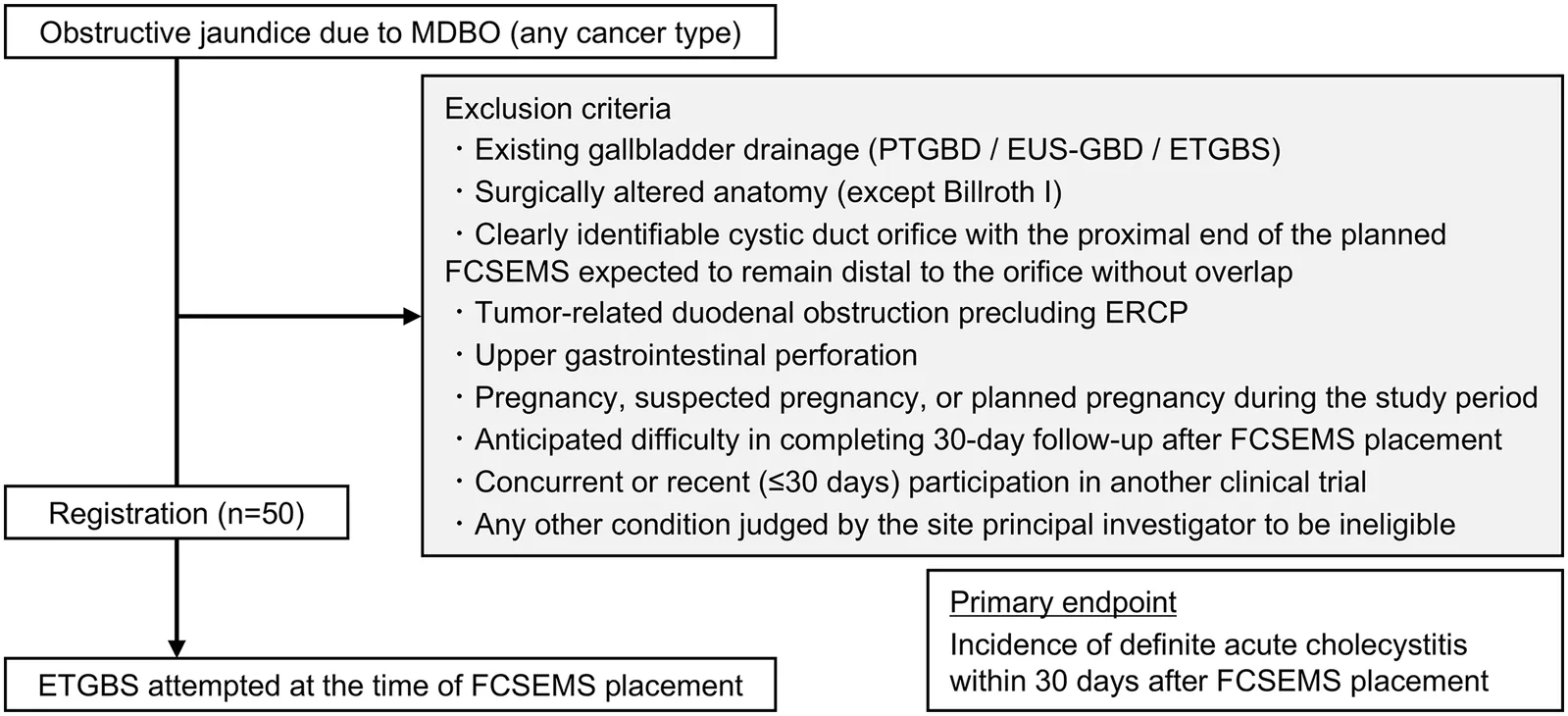
The study flow diagram. Patients with obstructive jaundice due to malignant distal biliary obstruction are screened according to the eligibility criteria. A total of 50 patients are planned to be enrolled, and ETGBS is attempted at the time of FCSEMS placement. The primary endpoint is the incidence of acute cholecystitis within 30 days after FCSEMS placement. CT, computed tomography; ERCP, endoscopic retrograde cholangiopancreatography; ETGBS, endoscopic transpapillary gallbladder stenting; EUS-GBD, endoscopic ultrasound-guided gallbladder drainage; FCSEMS, fully covered self-expandable metal stent; MDBO, malignant distal biliary obstruction; PTGBD, percutaneous transhepatic gallbladder drainage.

### Study status and timeline

Participant recruitment began on October 15, 2025 and is ongoing. Recruitment is planned to continue until March 31, 2028, and follow-up will continue until April 30, 2028. Any episode of acute cholecystitis occurring during follow-up, including its TG18 severity grade, will be recorded. Data cleaning and analysis will be performed after completion of follow-up, and the study results are expected to be submitted for publication in a peer-reviewed journal by March 31, 2030.

### Sample size

As this is an exploratory single-arm study designed to provide data for future comparative studies, no formal sample size calculation based on statistical power was performed. The target sample size was determined pragmatically based on the anticipated number of eligible patients who could be enrolled across the four participating institutions during the planned recruitment period. Accordingly, the target enrollment was set at 50 patients. Allowing for 10% of enrolled participants to be unevaluable for the primary endpoint because of withdrawal of consent, major protocol deviations, or unavailability of 30-day outcome data, we anticipate that 45 patients will be evaluable for the primary analysis. Technical failure of ETGBS will not be considered attrition. Participants with technically unsuccessful ETGBS will remain evaluable for the primary endpoint, provided that an ETGBS attempt has been initiated and the primary endpoint can be ascertained through day 30 or until an earlier competing event.

For planning purposes, and acknowledging that the available published data are not fully comparable with the present study, we estimated the expected precision of the primary endpoint based on previous reports. The historical benchmark of 12.1% was selected from the study by Ishii et al. [24] because it included a relatively large cohort of 339 patients who underwent initial placement of a 10-mm covered SEMS for distal malignant biliary obstruction, excluded patients with prior or concurrent gallbladder drainage, and evaluated early acute cholecystitis using the same 30-day period as the primary endpoint of the present study. In that study, early acute cholecystitis occurred in 41 of 339 patients (12.1%). Thus, despite the limitations inherent in using a single retrospective study as a historical reference, we considered this estimate the most directly comparable benchmark available. The incidence of acute cholecystitis within 30 days after FCSEMS placement was reported to be 3.1% when ETGBS was attempted [18]. Based on these findings, we assumed an incidence of 3% in the present study. Accordingly, with 45 evaluable patients and an assumed event rate of 3%, the two-sided 90% Wilson confidence interval is expected to be approximately 0.8% to 10.5%.

### Statistical analysis

#### Analysis populations.

The full analysis set (FAS) will serve as the primary analysis population and will include all enrolled participants in whom an ETGBS attempt is initiated and for whom at least one item of post-procedure outcome information is available, regardless of the technical success of ETGBS or subsequent treatment. Participants in whom an ETGBS attempt is not initiated, participants for whom no post-procedure outcome information is available, participants without baseline data, and participants with protocol violations that preclude inclusion in the FAS will be excluded. The number of excluded participants and the reasons for exclusion will be reported.

The per-protocol set (PPS) will be defined as a subset of the FAS, excluding participants with major protocol deviations related to eligibility. For PPS eligibility, such deviations will be defined as confirmed failure to meet any of the prespecified inclusion criteria (criteria 1–5) or fulfillment of any of the prespecified exclusion criteria (criteria 1–9) at enrollment, including violations identified after enrollment. For the expected survival criterion, eligibility will be based on the site principal investigator’s assessment at enrollment. Death occurring within 4 months after enrollment will not, in itself, be considered a violation of this criterion. Technical failure of ETGBS will not, in itself, constitute a major protocol deviation. The number of participants excluded from the PPS and the specific eligibility criteria violated will be reported. The safety analysis set (SAS) will include all enrolled participants in whom the protocol-defined ERCP procedure, undertaken with planned FCSEMS placement and ETGBS attempt, is initiated. Participants in whom biliary cannulation fails before initiation of an ETGBS attempt will therefore not be included in the FAS but will be included in the SAS. The number of such participants and the reasons for not initiating ETGBS will be reported.

The FAS was selected to evaluate the clinical strategy of attempting ETGBS at the time of FCSEMS placement, irrespective of its technical success. Therefore, participants with technically unsuccessful ETGBS will remain in the FAS if post-procedure outcome information is available. The PPS analysis will be supplementary. If the FAS and PPS are identical, this will be explicitly reported.

#### Descriptive analyses.

The number of participants in each analysis population will be summarized from all enrolled participants. Baseline demographic and clinical characteristics will be summarized descriptively using frequencies and percentages for categorical variables and medians with interquartile ranges for continuous variables. Baseline factors potentially associated with acute cholecystitis after FCSEMS placement, including gallstones and tumor involvement of the cystic duct orifice, will also be recorded and summarized descriptively. To explore center-level variability, the number of enrolled participants, key baseline characteristics, and primary endpoint event counts, denominators, and proportions will be summarized by institution. The same denominator criteria as in the overall primary analysis will be applied within each institution. Given the limited sample size and expected small number of events, no formal between-center comparisons or center-adjusted regression analyses are planned. Differences in recruitment, patient characteristics, and observed event rates across institutions will be considered when interpreting the pooled results, recognizing the limited precision of center-specific estimates.

#### Primary endpoint analysis.

In the primary analysis, the frequency and proportion of participants who develop definite acute cholecystitis within 30 days after FCSEMS placement will be calculated among participants in the FAS whose primary endpoint status is ascertainable. A two-sided 90% confidence interval will be calculated using the Wilson score method.

Participants who are lost to follow-up before day 30 and whose primary endpoint status cannot be ascertained will be treated as having missing primary endpoint data and will be excluded from the denominator used to calculate the primary endpoint proportion. This approach avoids assuming that participants with incomplete follow-up and unascertainable endpoint status remained free of acute cholecystitis throughout the 30-day period. As supplementary sensitivity analyses, the robustness of the primary endpoint estimate to missing outcome data will be assessed using best-case and worst-case scenarios. FAS participants with unascertainable primary endpoint status will be included in the denominator and classified as non-events in the best-case scenario and as events in the worst-case scenario. Participants classified as non-events under the prespecified rules for death without preceding definite acute cholecystitis or for cholecystectomy or gallbladder drainage performed for other indications will retain this classification in both scenarios. Event proportions and corresponding two-sided 90% Wilson score confidence intervals will be calculated under each scenario and compared descriptively with the primary analysis. Missing primary endpoint data will not be imputed in the primary analysis. The number of participants with unascertainable primary endpoint status, their available follow-up duration, and the reasons for loss to follow-up will be reported.

As a supplementary exploratory analysis, a two-sided one-sample score test for a proportion without continuity correction will be performed using a null proportion of 12.1% and a significance level of 10%. This analysis is intended to assess whether the observed incidence provides a preliminary signal relative to the historical benchmark and to inform the design of future comparative studies. Because of the single-arm design, the comparison will not by itself be interpreted as establishing a causal treatment effect or providing confirmatory evidence of efficacy. Differences in relevant baseline risk factors between the present study population and the historical cohort will be considered when interpreting the results, where corresponding historical data are available. No multivariable-adjusted analysis is planned because of the limited sample size and expected small number of events.

As supplementary analyses, a two-sided 95% Wilson score confidence interval will also be calculated for the primary endpoint, and the primary analysis will be repeated in the PPS. The incidence of acute cholecystitis within 30 days will also be summarized descriptively according to the FCSEMS model used, reporting event counts, denominators, and proportions with two-sided 90% Wilson score confidence intervals. No formal statistical comparison between FCSEMS models is planned.

#### Secondary endpoint analyses.

Among participants in the SAS in whom an ETGBS attempt is initiated, the technical success rate of ETGBS will be summarized using frequencies and proportions, with a two-sided 90% Wilson score confidence interval.

Endoscopic procedure-related adverse events will be summarized in the SAS using frequencies and proportions, with a two-sided 90% Wilson score confidence interval. The type and severity of adverse events will also be summarized descriptively.

Among participants with technically successful ETGBS, the incidence of definite acute cholecystitis within 30 days after FCSEMS placement will be summarized using frequencies and proportions, with a two-sided 90% Wilson score confidence interval. The incidence will also be summarized descriptively according to the type and caliber of the gallbladder stent placed, reporting event counts, denominators, and proportions with two-sided 90% Wilson score confidence intervals. Because gallbladder stent selection is not randomized, these descriptive summaries will not be interpreted as causal comparisons between the two stent types.

For definite acute cholecystitis occurring from day 31 onward, a landmark time-to-event analysis will be performed among participants who are alive and under follow-up at day 31 and have experienced neither definite acute cholecystitis nor a competing event before that time. Time will be measured from the day-31 landmark to the first definite acute cholecystitis event. Death before definite acute cholecystitis and cholecystectomy or gallbladder drainage performed for an indication other than definite acute cholecystitis will be treated as competing events. Cholecystectomy or gallbladder drainage performed for definite acute cholecystitis will be counted as an acute cholecystitis event. Participants without an event or competing event will be censored at the last follow-up assessment or the administrative end of follow-up on April 30, 2028, whichever occurs first. The cumulative incidence function will be estimated using the Aalen–Johansen method. The cumulative incidence curve will be reported. Among participants included in the landmark analysis, observed follow-up duration from the day-31 landmark to the end of observation will be summarized using the median and interquartile range (25th–75th percentiles). The TG18 severity grade and management of definite acute cholecystitis will be summarized descriptively. The guidewire and catheter models used will also be summarized descriptively; no formal device-specific comparisons are planned. No interim analysis is planned. All statistical analyses will be performed using R (R Foundation for Statistical Computing, Vienna, Austria).

### Data management and monitoring

Data will initially be recorded on paper case report forms by investigators at each participating institution. Completed case report forms, identified only by study-specific participant codes, will be submitted to the study office. No dedicated electronic data capture system will be used. Authorized study-office personnel will centrally enter the data into a password-protected Microsoft Excel database (Microsoft Corporation, Redmond, WA, USA). A designated data-management investigator will verify the electronic entries against the paper case report forms, and any discrepancies will be resolved through queries to the relevant institution. Corrections to the electronic database will be documented in a data-change log, and version-controlled copies will be retained. The database will be locked before statistical analysis. Identifiable participant information and de-identified study data will be stored separately, and access will be restricted to authorized study personnel. Electronic data will be stored on password-protected devices or electronic media, and paper records will be stored securely in locked cabinets. The Data Center at Yokohama City University Hospital will conduct central monitoring to ensure data quality, oversee study conduct, and review all adverse events recorded in the case report forms. The Data Center monitors safety data but does not independently make decisions regarding study suspension, and no fixed numerical stopping boundary has been prespecified. Any serious adverse event related or possibly related to ETGBS, or any concerning pattern of procedural adverse events, including cystic duct perforation, will be promptly reported and trigger an ad hoc safety review by the study representative in consultation with the participating investigators and the Ethics Committee. Enrollment may be suspended, the protocol modified, or the study terminated if continuation is considered unsafe. Records will be retained until the later of 5 years after the report of study completion or 3 years after the final publication of the study results. De-identified data will be retained beyond this period, provided that consent for secondary use has been obtained at enrollment, to allow potential use in future research as appropriate.

### Ethics and dissemination

The study will be conducted in accordance with the principles of the Declaration of Helsinki and has been approved by the Ethics Committee of Yokohama City University Hospital (approval number: F250904002). The initial protocol version 1.0 was approved on September 4, 2025. Protocol versions 1.1 and 1.2 involved only organizational changes to the participating institutions and site principal investigators and did not alter the study design or analysis plan. The current protocol version 1.3, approved on September 3, 2026, incorporated revisions to eligibility assessment, procedural definitions, data collection, safety monitoring, and statistical analysis without changing the study intervention or primary endpoint. The study was registered with the Japan Registry of Clinical Trials (jRCT1030250361) on September 12, 2025. The registry record has been updated to reflect the current approved study information. The study overview and recruitment status are publicly available in the registry. Any future substantive protocol amendments will be submitted to the Ethics Committee for approval before implementation, communicated to all participating institutions, and reflected in the trial registry as applicable. The results will be submitted for presentation at an international conference and for publication in an international peer-reviewed journal.

## Discussion

For MDBO, FCSEMSs generally provide longer patency than plastic stents and are recommended, including on cost-effectiveness grounds, when survival of at least 4 months is anticipated [1,4–6,25]. However, FCSEMSs may compress the cystic duct orifice and/or pancreatic duct orifice, potentially increasing the risk of complications such as acute cholecystitis and pancreatitis [15–17]. By contrast, in patients with an anticipated survival of 3 months or less, plastic stents may be selected for short-term drainage. For these reasons, an expected survival of at least 4 months was included in the inclusion criteria for the present study.

The incidence of acute cholecystitis after FCSEMS placement for MDBO has been reported to range from 6.5% to 15.3% [26–29]. Proposed mechanisms and risk factors include gallstones, tumor involvement of the cystic duct orifice, mechanical obstruction of the cystic duct orifice by FCSEMS, and reflux of contrast agents into the gallbladder during cholangiography [16,17,24,29]. Acute cholecystitis may require additional invasive interventions and delay subsequent cancer treatment; it may also be life-threatening in frail patients. Preventing this complication is therefore clinically important.

Prophylactic ETGBS at the time of FCSEMS placement has been proposed as a strategy to maintain bile outflow from the gallbladder, prevent cystic duct orifice obstruction, and thereby reduce the risk of subsequent acute cholecystitis. Nevertheless, the current evidence base is limited to three single-center retrospective studies [18–20]. As summarized in Table 1, these reports suggest that the incidence of cholecystitis after FCSEMS placement may be lower when ETGBS is technically successful. Ishii et al. reported a technical success rate of 75.0%, with cholecystitis occurring in 4.2% of technically successful ETGBS cases [18]. Kozakai et al. and Gosain et al. reported technical success rates of 70.0% and 57.9%, respectively, with no cases of cholecystitis among patients with technically successful ETGBS [19,20]. However, randomized comparative trials have not yet been conducted.

**Table 1:** Summary of studies evaluating prophylactic ETGBS.

References	Study design	Patients with attempted ETGBS, n	Technical success of ETGBS, n (%)	Cholecystitis after successful ETGBS, n (%)	Cholecystitis after failed ETGBS, n (%)	Adverse events (n)
Ishii *et al.* [18]	Single-center retrospective study	32	24 (75.0)	1 (4.2)	NR	Pancreatitis (2)
						Perforation of cystic duct (1)
Kozakai *et al.* [19]	Single-center retrospective study	30	21 (70.0)	0 (0)	2 (22.2)	Pancreatitis (7)
						Non-obstructive cholangitis (1)
						Perforation of cystic duct (1)
Gosain *et a*l. [20]	Single-center retrospective study	19	11 (57.9)	0 (0)	2 (25.0)	Perforation of cystic duct (3)

ETGBS, endoscopic transpapillary gallbladder stenting; n, number of cases; NR, not reported.

In the present study, the primary endpoint is the incidence of acute cholecystitis within 30 days after FCSEMS placement. This time window was selected because cholecystitis after FCSEMS placement tends to occur early. Ishii et al. reported that 41 of 51 acute cholecystitis cases (80.4%) occurred within 30 days after FCSEMS placement [24], and Kozakai et al. reported a median time to onset of 18 days after FCSEMS placement [19].

In patients with MDBO, the reported technical success rate of ETGBS ranges from 57.9% to 75.0%, indicating that technical success is not consistently achieved [18–20]. By contrast, in patients undergoing ETGBS for acute cholecystitis without concomitant MDBO, reported technical success rates range from 72.8% to 84.6% [30–32]. In MDBO, tumor-related biliary narrowing may limit guidewire manipulation, making cannulation and advancement into the cystic duct and gallbladder more difficult. The technical success rate of ETGBS may be lower in this population.

Cholangiography is routinely performed during ERCP to delineate the biliary anatomy. However, when ETGBS is attempted, additional cholangiography may be required to identify the cystic duct orifice and delineate the course of the cystic duct, potentially increasing the volume of contrast agent administered and the risk of reflux into the gallbladder. If ETGBS is unsuccessful, repeated attempts could theoretically increase the volume of refluxed contrast agent and thereby increase the risk of cholecystitis [16,29]. Consistent with this concern, Kozakai et al. and Gosain et al. reported cholecystitis among patients with unsuccessful ETGBS in 2 of 9 and 2 of 8 patients, respectively [19,20]. Although the numbers are small, these observations raise the possibility that unsuccessful ETGBS attempts may be associated with an increased risk of cholecystitis. In addition, procedure-related complications such as cystic duct perforation have been reported [18–20]. Thus, whether ETGBS should be attempted routinely at the time of FCSEMS placement remains uncertain.

Given these uncertainties, we designed a multicenter, prospective, single-arm exploratory study to explore the potential clinical benefit of attempting prophylactic ETGBS by estimating the incidence of acute cholecystitis after FCSEMS placement and characterizing the technical feasibility and safety of the strategy in patients with MDBO. This approach will capture outcomes among all patients in whom ETGBS is attempted, rather than limiting the evaluation to technically successful cases alone. This study has several limitations. First, because this is a single-arm study without randomization or a concurrent control group, it is designed to estimate feasibility, safety, and event rates rather than to establish the efficacy of prophylactic ETGBS. Any comparison with a non-ETGBS strategy will rely on historical data and may be confounded by differences in patient characteristics, stent selection, and procedural techniques. Although relevant baseline risk factors will be described and considered when interpreting the historical comparison, residual confounding cannot be excluded. Therefore, the exploratory one-sample score test without continuity correction will be interpreted as a hypothesis-generating comparison with the historical benchmark rather than as confirmatory evidence of treatment efficacy. Second, although all operators and assistants are required to have performed at least 200 ERCP procedures as the primary operator, this threshold reflects general ERCP experience and does not necessarily ensure ETGBS-specific competency. Technical success and safety may therefore depend on individual ETGBS experience, and generalizability to less experienced or lower-volume settings may be limited. Third, although prespecified imaging criteria and independent assessment by two experienced endoscopists will be used, no blinded central imaging review or formal assessment of interobserver agreement is planned. Some site-level interobserver variability may therefore remain and may limit the reproducibility and external validity of exclusion criterion 3. Fourth, all four participating institutions are located in Kanagawa Prefecture, which may limit the geographic generalizability of the findings. Fifth, excluding participants with unascertainable primary endpoint status may introduce selection bias and reduce precision. If their risk of acute cholecystitis differs from that of participants with ascertainable outcomes, the estimated event proportion may be underestimated or overestimated.

To the best of our knowledge, this is the first multicenter prospective study to explore the potential clinical benefit of attempting prophylactic ETGBS during FCSEMS placement for MDBO and to characterize the technical feasibility and safety of this strategy. The decision to proceed to a future randomized trial will be based on the totality of the findings, including technical feasibility, procedure-related safety, and the observed 30-day incidence of acute cholecystitis. As a quantitative framework for interpreting the results, an upper limit of the two-sided 90% confidence interval below the historical benchmark of 12.1% will be considered supportive of proceeding to a future randomized trial, whereas achievement of the same criterion using the two-sided 95% confidence interval will be regarded as stronger supportive evidence. These criteria will be interpreted together with technical feasibility and procedure-related safety and will not, by themselves, be considered confirmatory evidence of efficacy. Conversely, low technical success, safety concerns, or an incidence that does not suggest potential benefit would indicate the need to refine the intervention or study design before proceeding to a randomized trial. The findings may therefore help clarify the potential role of this strategy in clinical practice and determine whether and how it should be evaluated in a future randomized trial.

## Supporting information

S1 File SPIRIT checklist. (DOCX)

S2 File Study protocol, version 1.3 (original Japanese-language version). (DOCX)

S3 File Study protocol, version 1.3 (English translation of S2 File). (DOCX)
