# Supplementary material for: Prophylactic endoscopic transpapillary gallbladder stenting at fully covered self-expandable metal stent placement to prevent cholecystitis in malignant distal biliary obstruction: A multicenter prospective single-arm study protocol (PROTECT study)
Source: PLoS One. 2026 Oct 29;21(10):e0361468. doi: 10.1371/journal.pone.0361468 (PMC13718957; doi:10.1371/journal.pone.0361468)
Supplement: Supplementary file 2 [file pone.0361468.s002.docx]

研究計画書

研究課題名：

悪性遠位胆管狭窄に対する、fully covered self-expandable metallic stent (FCSEMS)留置後の胆嚢炎発症予防の観点から、endoscopic gallbladder stenting (EGBS)の有効性・安全性をみる多施設・単群臨床試験

研究代表者：

横浜市立大学附属病院　　肝胆膵消化器病学　小田貴之

版数：第1.3版

作成年月日：2026年7月28日

改訂履歴

| 版数 | 作成/改訂日 |
| --- | --- |
| 第1.0版 | 2025年8月18日 |
| 第1.1版 | 2026年2月4日 |
| 第1.2版 | 2026年4月6日 |
| 第1.3版 | 2026年7月28日 |

**機密情報保全**

本研究計画書は、公立大学法人横浜市立大学（以下「本学」という。）が所有する機密情報を含んでいます。

そのため、本研究計画書に従って臨床研究を実施する研究機関、研究者等および審査等業務を行う倫理審査委員会等の本研究計画書関係者以外の第三者には、公立大学法人横浜市立大学の事前の承諾なしに、本研究計画書の一部又は全部を開示することはできません。また、当該目的以外に使用することも固く禁じます。

**本学の臨床研究に関する個人情報の利用目的について**

　本学は、個人情報の保護に関する法律（平成15年法律第57号、以下「個情法」という。）第16条第8項に規定される「学術研究機関等」に該当し、大学その他の学術研究を目的とする機関である。

本学で実施する臨床研究は、個情法第18条第3項第5号及び第6号に規定される個人情報及び個人データについて学術研究目的で取り扱われる。

内容

[**0. 概要**	7](#_Toc82429667)

[**0.1.** **シェーマ**	7](#_Toc82429668)

[**0.2. 研究の目的と主要評価項目**	7](#_Toc82429669)

[**0.3. 研究対象者**	7](#_Toc82429670)

[**0.4. 治療**	7](#_Toc82429671)

[**0.5. 目標症例数**	7](#_Toc82429672)

[**0.6. 研究期間**	8](#_Toc82429673)

[**0.7. 研究代表者と問い合わせ先**	8](#_Toc82429674)

[**1. 本研究の目的**	9](#_Toc82429675)

[**2. 背景と試験計画の科学的根拠**	9](#_Toc82429676)

[**2.1. 対象疾患について**	9](#_Toc82429677)

[**2.2. 標準治療について**	9](#_Toc82429678)

[**2.3. 試験治療について**	10](#_Toc82429679)

[**2.4. 試験デザインと主要評価項目について**	11](#_Toc82429680)

[**2.5 本研究の意義**	11](#_Toc82429681)

[**3. 薬物／機器情報**	12](#_Toc82429682)

[**3.1. FCSEMSとして用いるデバイス**	12](#_Toc82429683)

[**3.2. EGBSの際に用いるデバイス**	12](#_Toc82429684)

[**4. 本研究で用いる規準・定義**	14](#_Toc82429685)

[**5. 研究対象者の選定方針**	14](#_Toc82429686)

[**5.1. 選択基準**	14](#_Toc82429687)

[**5.2. 除外基準**	14](#_Toc82429688)

[**6. 研究計画**	15](#_Toc82429689)

[**6.1. 研究デザイン**	15](#_Toc82429690)

[**6.2. 目標症例数**	15](#_Toc82429691)

[**6.3. 研究期間**	15](#_Toc82429692)

[**6.4. 割付方法**	15](#_Toc82429693)

[**6.5. 治療計画**	15](#_Toc82429694)

[**7. 観察・検査・調査・評価項目**	17](#_Toc82429695)

[**7.1. スケジュール表**	17](#_Toc82429696)

[**7.2. 実施スケジュール時期と評価項目**	18](#_Toc82429697)

[**7.3. 評価の方法**	19](#_Toc82429698)

[**8. 評価項目**	19](#_Toc82429699)

[**8.1. 主要評価項目**	19](#_Toc82429700)

[**8.2. 副次評価項目**	20](#_Toc82429701)

[**9. 統計解析**	20](#_Toc82429702)

[**9.1. 解析対象集団**	20](#_Toc82429703)

[**9.2. 目標症例数の設定根拠**	21](#_Toc82429704)

[**9.3. 統計解析方法**	21](#_Toc82429705)

[**9.4. 中間解析**	22](#_Toc82429706)

[**10. 有害事象の取り扱いについて**	23](#_Toc82429707)

[**10.1. 有害事象の定義**	23](#_Toc82429708)

[**10.2. 有害事象の評価**	23](#_Toc82429709)

[**10.3. 予期される有害事象等**	24](#_Toc82429710)

[**10.4. 有害事象が発現した場合の措置**	24](#_Toc82429711)

[**11. 効果安全性評価委員会**	25](#_Toc82429712)

[**12.研究参加に伴い研究対象者に予測される利益及び不利益等**	25](#_Toc82429713)

[**12.1. 予想される利益**	25](#_Toc82429714)

[**12.2. 負担並びに予想されるリスク**	25](#_Toc82429715)

[**13. インフォームド・コンセントを受ける手続**	25](#_Toc82429716)

[**13.1. 研究対象者等及びその関係者からの相談等への対応**	26](#_Toc82429717)

[**13.2. 代諾者等からインフォームド・コンセントを受ける場合**	26](#_Toc82429718)

[**13.3. インフォームド・アセントを得る場合**	26](#_Toc82429719)

[**13.4. 指針第8の8の規定による研究を実施しようとする場合**	26](#_Toc82429720)

[**14. 施設登録及び症例登録**	26](#_Toc82429721)

[**14.1. 登録センター**	26](#_Toc82429722)

[**14.2. 施設登録**	27](#_Toc82429723)

[**14.3. 症例登録**	27](#_Toc82429724)

[**15. 試料/情報の授受**	27](#_Toc82429725)

[**16. 試料・情報の保管及び廃棄の方法**	27](#_Toc82429726)

[**16.1. 試料・情報の二次利用について**	29](#_Toc82429727)

[**17. 個人情報等の取扱い**	29](#_Toc82429728)

[**18. 研究対象者に係る研究結果（偶発的所見を含む．）の取扱い**	29](#_Toc82429729)

[**18.1.遺伝カウンセリングの体制**	30](#_Toc82429730)

[**19. 研究計画書の遵守、逸脱及び変更について**	30](#_Toc82429731)

[**19.1. 研究計画書の遵守**	30](#_Toc82429732)

[**19.2. 研究計画書からの逸脱**	30](#_Toc82429733)

[**19.3. 研究計画書の変更手順**	30](#_Toc82429734)

[**20. 研究実施に係る手続**	30](#_Toc82429735)

[**20.1 遵守すべき諸規則**	30](#_Toc82429736)

[**20.2.倫理審査委員会への付議**	31](#_Toc82429737)

[**20.3.研究機関の長による許可等**	31](#_Toc82429738)

[**20.4.定期報告**	31](#_Toc82429739)

[**20.5.研究の停止・中止**	31](#_Toc82429740)

[**20.6.研究の終了**	32](#_Toc82429741)

[**21.研究の資金源等，研究機関の研究に係る利益相反及び個人の収益等，研究者等の研究に係る利益相反に関する状況**	32](#_Toc82429742)

[**21.1. 資金源及び財政上の関係**	32](#_Toc82429743)

[**21.2. 利益相反**	32](#_Toc82429744)

[**22.　研究対象者の費用負担・謝礼について**	32](#_Toc82429745)

[**23. 健康被害に対する補償**	32](#_Toc82429746)

[**24. モニタリング及び監査**	33](#_Toc82429747)

[**24.1. モニタリング**	33](#_Toc82429748)

[**24.2. 監査**	33](#_Toc82429749)

[**25. 研究の情報公開及び結果公表**	34](#_Toc82429750)

[**25.1. 研究の登録**	34](#_Toc82429751)

[**25.2. 研究結果の公表**	34](#_Toc82429752)

[**26．研究成果の帰属（知的財産権）**	34](#_Toc82429753)

[**27. 研究の実施体制**	34](#_Toc82429754)

[**27.1. 研究代表者**	34](#_Toc82429755)

[**27.2. 研究事務局**	34](#_Toc82429756)

[**27.3. 共同研究機関および研究責任者**	34](#_Toc82429757)

[**27.4.　研究協力機関と責任者**	35](#_Toc82429758)

[**27.5. 統計解析責任者**	35](#_Toc82429759)

[**27.6. データセンター/データマネージメント責任者**	35](#_Toc82429760)

[**27.7. モニタリング責任者**	35](#_Toc82429761)

[**27.8. 監査責任者**	35](#_Toc82429762)

[**27.9. 外部委託機関**	35](#_Toc82429763)

[**28. 文献**	36](#_Toc82429764)

**略語及び用語の定義**

本研究計画書に使用する略語及び用語を下記に示す。

| 略語 | 正式名称 | 説明 |
| --- | --- | --- |
| CRP | C-reactive protein | C-反応性蛋白 |
| EGBS | endoscopic gallbladder stenting | 内視鏡的胆嚢留置ステント |
| ERCP | endoscopic retrograde cholangiopancreatography | 内視鏡的逆行性胆管膵管造影 |
| ESGE | European Society for Gastrointestinal Endoscopy | 欧州消化器内視鏡学会 |
| EUS-BD | endoscopic ultrasonography-guided biliary drainage | 超音波内視鏡ガイド下胆道ドレナージ |
| EUS-GBD | endoscopic ultrasonography-guided gallbladder drainage | 超音波内視鏡ガイド下胆嚢ドレナージ |
| FCSEMS | fully covered self-expandable metallic stent | 被覆型自己拡張型金属製ステント |
| PTBD | percutaneous transhepatic biliary drainage | 経皮経肝胆道ドレナージ |
| PTGBA | percutaneous transhepatic gallbladder aspiration | 経皮経肝胆嚢吸引穿刺法 |
| PTGBD | percutaneous transhepatic gallbladder drainage | 経皮経肝胆嚢ドレナージ |
| RCT | randomized controlled trial | ランダム化比較試験 |
| UCSEMS | uncovered self-expandable metallic stent | 非被覆型自己拡張型金属製ステント |
|  |  |  |
|  |  |  |
|  |  |  |
|  |  |  |
|  |  |  |
|  |  |  |
|  | l |  |
|  |  |  |
|  |  |  |
|  |  |  |
|  |  |  |
|  |  |  |
|  |  |  |
|  |  |  |
|  |  |  |
|  |  |  |
|  |  |  |

# 0. **概要**

## **0.1.** **シェーマ**

対面説明

文書同意取得

登録

除外対象：急性胆嚢炎を合併している症例、胆嚢摘出術後の症例、FCSEMSが胆嚢管開口部を塞がないと予想される症例、同意が得られない症例

試験治療群（介入群）

FCSEMS留置時にendoscopic gallbladder stenting (EGBS)を試みる

悪性遠位胆管狭窄でfully covered self-expandable metallic stent (FCSEMS)を留置する、

登録時年齢が18歳以上の男女

主要評価項目：FCSEMS留置後30日以内の急性胆嚢炎発症割合

（胆嚢炎の診断は「急性胆管炎・胆嚢炎診療ガイドライン2018」に基づいて行う）

副次評価項目：EGBSの技術的成功率、EGBSに関連した合併症の発症割合、EGBS成功例でのFCSEMS留置後の胆嚢炎発症割合、FCSEMS留置後31日以降の胆嚢炎発症割合

## **0.2. 研究の目的と主要評価項目**

目的：悪性遠位胆管狭窄に対してfully covered self-expandable metallic stent (FCSEMS)を留置する際に、endoscopic gallbladder stenting (EGBS)を併用することで、術後の急性胆嚢炎発症リスクを低減させる事ができるか検討する。

主要評価項目：EGBSを試みた全症例での、FCSEMS留置後30日以内の胆嚢炎発症割合

## **0.3. 研究対象者**

18歳以上の悪性遠位胆管狭窄による閉塞性黄疸をきたした患者

## 0.4. **治療**

悪性遠位胆管狭窄に対してFCSEMSを留置する際に、適格症例に対してEGBSを試みる

## **0.5. 目標症例数**

50症例

## **0.6. 研究期間**

研究期間：研究機関の長の実施許可日から2030年3月31日まで（5年間）

登録期間：研究機関の長の実施許可日から2028年3月31日まで

追跡期間：2028年4月30日まで

## **0.7. 研究代表者と問い合わせ先**

所属：　　　　横浜市立大学附属病院　肝胆膵消化器病学教室

氏名：　　　　小田　貴之

問い合わせ先： t246016b@yokohama-cu.ac.jp

# **1. 本研究の目的**

悪性遠位胆管狭窄を来した患者に対して、FCSEMSを留置する際のEGBSの有効性を、術後の急性胆嚢炎発症割合の観点から、単群で探索的に検討する。

# **2.** **背景と試験計画の科学的根拠**

## **2.1. 対象疾患について**

膵癌や胆道癌などの悪性疾患の進行による胆管閉塞により閉塞性黄疸を来すことがある。本邦での統計では、2020年に膵癌と診断された患者は44448人、胆道癌と診断された患者は21392人であり、共に罹患数は増加傾向にある[1]。 罹患患者の増加に伴い、悪性遠位胆管狭窄による閉塞性黄疸を来す患者数も増加傾向にあることが予想される。閉塞性黄疸の進行は肝不全や急性胆管炎の発症に繋がる可能性があり、閉塞を解除するためにfully covered self-expandable metallic stent (FCSEMS)を留置することがある。FCSEMS留置後に急性胆嚢炎を発症することがあり、その頻度は15%と報告されている[2]。急性胆嚢炎の発症によりその後の手術への移行や化学療法の導入が遅れ、予後に大きく影響することがある。FCSEMS留置時のEGBSには、術後の胆嚢炎の発症リスクを低減する可能性があるため、本研究を計画した。

## **2.2. 標準治療について**

**2.2.1. 現在の標準治療**

悪性遠位胆管狭窄に対する治療として、endoscopic retrograde cholangiopancreatography（ERCP）による胆道ドレナージが標準治療となっている[3]。その他の胆道ドレナージの方法として、percutaneous transhepatic biliary drainage（PTBD）やendoscopic ultrasonography-guided biliary drainage（EUS-BD）の有用性も報告されている。しかしながら悪性胆管狭窄に対してERCPおよびPTBDを実施した症例を後ろ向きに検討した報告では、偶発症発生率がERCPと比較しPTBDで高い（8.6% vs 12.3%）と報告されている[4]。他の報告でも、ERCPに比しPTBDの方が死亡率が高い（4.35% vs 6.86%）とされている[5]。ERCPとEUS-BDを比較したRCTでは[6-8]、手技成功率、治療奏効率は同等で、偶発症発生率も差はないという結果であったが、EUS-BDは手技的難易度がERCPより高く、不成功による偶発症は時に致死的となるため、ERCP困難症例での代替治療という位置づけである。以上からERCPによる胆道ドレナージが第一選択となる。

**2.2.2. 現在の標準治療の有効性に関して**

ERCPによる胆道ドレナージの際に使用するステントとしてプラスチックステントと金属ステントがある。悪性遠位胆管狭窄症例においては、ステント径の大きい金属ステントの方がより開存期間が長いとされ、広く使用されている[9, 10] 。Davidsらはプラスチックステントの開存期間の中央値は126日であったのに対して、金属ステントでは開存期間の中央値は273日と有意に長かったと報告している[10]。European Society for Gastrointestinal Endoscopy (ESGE)によるガイドラインでも、予後が4カ月以上見込まれる遠位胆管悪性閉塞症例においては、費用対効果の観点からも金属ステントの留置を推奨している[11]。金属ステントには金属メッシュ間がシリコーンでカバーされているFCSEMSと、 カバーされていないuncovered self-expandable metallic stent (UCSEMS)がある。UCSEMSを悪性遠位胆管狭窄に用いると、腫瘍が金属ステントメッシュ間隙から胆管内に進展する、所謂tumor ingrowthによるステント閉塞が問題となる。FCSEMSはこのtumor ingrowthを予防できることから、開存期間の中央値が304日と、UCSEMSの開存期間中央値166日と比較して長いと報告されており、有事の際は抜去できる事からも、FCSEMSの優位性が示されている[12]。

**2.2.3. 現在の標準治療の安全性に関して**

FCSEMS留置後の合併症として、長期留置に伴うステント機能不全の頻度が20%と報告されている[11]。 その他急性膵炎（発症率：約8%）[13]や急性胆嚢炎の合併症も報告されている。IshiiらはFCSEMSに限定した後方視的検討で、FCSEMS留置後の急性胆嚢炎の頻度は15%と報告している[2]。

**2.2.4. 現在の標準治療の課題点**

2.2.3で述べた様に、FCSEMS留置後の合併症の1つに急性胆嚢炎がある。金属ステントが胆嚢管開口部を閉塞する事、胆管造影した際の造影剤が胆嚢へ逆流する事が急性胆嚢炎の発症に関連すると報告されている[14]。急性胆嚢炎を発症するとpercutaneous transhepatic gallbladder aspiration（PTGBA）、percutaneous transhepatic gallbladder drainage (PTGBD)、endoscopic ultrasonography-guided gallbladder drainage (EUS-GBD)、胆嚢摘出術などの観血的処置を必要とすることがある。急性胆嚢炎の発症はその後の手術への移行や化学療法の導入の遅れにつながり、致命的となることがある[15]。

## **2.3. 試験治療について**

**2.3.1. 試験治療の概要**

2.2.4項で記載の様に、FCSEMS留置後の急性胆嚢炎発症の機序として、胆嚢管開口部のステントによる閉塞が考えられている[14]。FCSEMS留置の際に、Endoscopic gallbladder stenting (EGBS) を行うことで、FCSEMS留置後も胆嚢内の胆汁が十二指腸に排泄され、FCSEMS留置後の急性胆嚢炎発症リスクを低減させることができると期待される。

**2.3.2. 試験治療の有効性に関して**

悪性遠位胆管狭窄症例にFCSEMSを留置する際のEGBS併用の有効性に関して2つの報告がある。 Gosainらは19例の悪性遠位胆管狭窄症例でFCSEMS留置の際にEGBSを試み、11例（58%）でEGBSに成功し、胆嚢炎を含め処置後の合併症は0例であったと報告している[16]。またIshiiらはEGBS併用群と、非併用群で後方視的比較検討を行い、併用群で胆嚢炎の発症割合は4%であったのに対して、EGBS非併用群では21%であり、悪性遠位胆管狭窄に対してFCSEMS留置時にEGBSを行うことで有意に胆嚢炎の発症割合が低下したと報告している[15]。Ishiiらの報告ではEGBSの成功割合は75%であり、EGBSを試みた群での胆嚢炎の発症割合は3.1%であった[15]。

**2.3.3. 試験治療の安全性に関して**

2.3.2項で記載した同様の報告から記載する。Gosainらの報告では, EGBSが不成功に終わった8例のうち3例（37.5%）はガイドワイヤーが胆嚢管を穿通し、うち1例は緊急胆嚢摘出術を必要としたと報告している[16]。またIshiiらの報告では、EGBSを試みた32例のうち1例（3.1%）で急性胆嚢炎、2例（6.3％）でERCP後膵炎、1例（3.1%）でガイドワイヤーの胆嚢管穿通が生じたと報告している[15]。

**2.3.4. 試験治療のリスク・ベネフィットについて**

試験治療が被験者にもたらすリスクをまず述べる。悪性遠位胆管狭窄に対してFCSEMS留置時のEGBSの有効性に関する既報[15, 16]は少なく、前向き比較試験は実施されていない。EGBSの成功率はおよそ70%程度[15]とされている。EGBSを行う際は胆道造影を行い胆嚢管の走行を確認するため、EGBSを行わない際より造影剤の使用量が多くなる可能性がある。胆嚢への造影剤の逆流が処置後の胆嚢炎のリスクを上げる[17]という報告もあり、EGBSを試みたが失敗に終わった症例では、処置後の急性胆嚢炎の発症リスクを上げてしまう可能性がある。またEGBSを試みた際のガイドワイヤーの胆嚢管の穿通というリスクもあり、既報ではEGBSを試みたことでFCSEMSの留置が困難となった例は報告されていないが、場合によってはFCSEMS留置が困難となる可能性もある。その他EGBSを試みることで、ERCP全体の処置時間が延長することが予想される。一方で試験治療がもたらすベネフィットは前述のとおり、FCSEMS留置後の胆嚢炎の頻度を減らすことができる可能性を有する事である。胆嚢炎を予防することで、化学療法の導入が早期にでき、患者の予後を改善する可能性がある。2.3.2項および2.3.3項で述べたEGBSの有効性および安全性のデータからは、FCSEMS留置の際のEGBSが被験者にもたらすベネフィットはリスクを上回る可能性が高いと考える。

## **2.4. 試験デザインと主要評価項目について**

**2.4.1. 試験デザインについて**

本試験のデザインは前向きの単群での探索的試験である。2.3.2項で記載したように悪性遠位胆管狭窄に対するFCSEMS留置時のEGBSの有効性に関しての報告は少ない。そのため、まずはその有効性に関して探索的検討を行う。参加4施設の過去数年、悪性遠位胆管狭窄に対してFCSEMSを留置した症例数から、年間組み込み症例数は27例程度と予想され、実施可能性の観点からまずは単群での探索的検討を予定する。本試験でEGBSの有効性の可能性が示された場合、今後検証的試験を予定する。

**2.4.1. 主要評価項目について**

主要評価項目は「EGBSを試みた全症例での、FCSEMS留置後30日以内の急性胆嚢炎発症割合」と設定する。本研究の目的は悪性遠位胆管狭窄を来した症例に対してFCSEMSを留置する際に、EGBSを行うことで術後の胆嚢炎の発症割合を下げることができるのか、探索的に検討することである。そのため主要評価目的をFCSEMS留置後の胆嚢炎発症割合と定めた。既報ではFCSEMS留置後の胆嚢炎の約8割は30日以内に集積すると報告されている[2]ことを参考とし、30日以内の胆嚢炎発症割合と定めた。

＜標準治療について＞

- 標準治療（治療の現状）について説明する。研究計画の時点で標準治療を決めた臨床試験について、その試験デザインや主な有効性・安全性のデータ（数値）を記述する。また、その標準治療でのunmet medical needs（標準治療で困っている事，足りない事が何か）を記載する。
- 現在の標準治療を決めた臨床試験があるはずなので，その試験についての情報（研究デザイン，有効性や安全性に関する数値データ）を記載する。
- 標準治療が確立されていない場合はその旨を明記し、広く用いられている治療が何かを記述する。

＜標準治療について＞

- 標準治療（治療の現状）について説明する。研究計画の時点で標準治療を決めた臨床試験について、その試験デザインや主な有効性・安全性のデータ（数値）を記述する。また、その標準治療でのunmet medical needs（標準治療で困っている事，足りない事が何か）を記載する。
- 現在の標準治療を決めた臨床試験があるはずなので，その試験についての情報（研究デザイン，有効性や安全性に関する数値データ）を記載する。
- 標準治療が確立されていない場合はその旨を明記し、広く用いられている治療が何かを記述する。

＜標準治療について＞

- 標準治療（治療の現状）について説明する。研究計画の時点で標準治療を決めた臨床試験について、その試験デザインや主な有効性・安全性のデータ（数値）を記述する。また、その標準治療でのunmet medical needs（標準治療で困っている事，足りない事が何か）を記載する。
- 現在の標準治療を決めた臨床試験があるはずなので，その試験についての情報（研究デザイン，有効性や安全性に関する数値データ）を記載する。
- 標準治療が確立されていない場合はその旨を明記し、広く用いられている治療が何かを記述する。

＜標準治療について＞

- 標準治療（治療の現状）について説明する。研究計画の時点で標準治療を決めた臨床試験について、その試験デザインや主な有効性・安全性のデータ（数値）を記述する。また、その標準治療でのunmet medical needs（標準治療で困っている事，足りない事が何か）を記載する。
- 現在の標準治療を決めた臨床試験があるはずなので，その試験についての情報（研究デザイン，有効性や安全性に関する数値データ）を記載する。
- 標準治療が確立されていない場合はその旨を明記し、広く用いられている治療が何かを記述する。

＜標準治療について＞

- 標準治療（治療の現状）について説明する。研究計画の時点で標準治療を決めた臨床試験について、その試験デザインや主な有効性・安全性のデータ（数値）を記述する。また、その標準治療でのunmet medical needs（標準治療で困っている事，足りない事が何か）を記載する。
- 現在の標準治療を決めた臨床試験があるはずなので，その試験についての情報（研究デザイン，有効性や安全性に関する数値データ）を記載する。
- 標準治療が確立されていない場合はその旨を明記し、広く用いられている治療が何かを記述する。

＜標準治療について＞

- 標準治療（治療の現状）について説明する。研究計画の時点で標準治療を決めた臨床試験について、その試験デザインや主な有効性・安全性のデータ（数値）を記述する。また、その標準治療でのunmet medical needs（標準治療で困っている事，足りない事が何か）を記載する。
- 現在の標準治療を決めた臨床試験があるはずなので，その試験についての情報（研究デザイン，有効性や安全性に関する数値データ）を記載する。
- 標準治療が確立されていない場合はその旨を明記し、広く用いられている治療が何かを記述する。

## **2.5 本研究の意義**

本研究でEGBSの有効性の可能性を示せた場合、FCSEMS留置の際のEGBSの併用が標準治療となり、FCSEMS留置後の胆嚢炎の発症割合を下げ、癌患者の予後の改善につながる可能性があり、有益性は高いと考える。2.3.2項で示したように、本研究に関連した報告はこれまで少なく、EGBSの有効性に関しては明確に示されていない。EGBSの成功率は70%程度[15]とされており、失敗に終わる症例では、処置時間の増加や、胆嚢への造影剤の逆流など、かえって胆嚢炎の発症割合を増加させる可能性も予想される。EGBSを試みても、FCSEMS留置後の胆嚢炎発症割合を低減させる可能性を示せなかった場合は、反対にEGBSはFCSEMS留置時に不要である可能性を示せるため、重要な知見となり得ると考える。

# **3. 機器情報**

## **3.1. FCSEMSとして用いるデバイス**

本研究でFCSEMSとして用いるデバイスは以下の2つである。両デバイスとも10mm径のものを使用する。ステント長に関しては、症例毎に責任者が適当と判断した長さのものを使用する。

①商品名：HANAROSTENT Biliary Full Cover NEO

製造販売会社名：Boston Scientific社

使用目的又は効果：

本品は、内視鏡的、経皮的、開腹術下に胆嚢（胆嚢、胆嚢管 及び胆管系）に挿入し、排のう、排液、灌流、狭窄部位の拡張・ 支持、狭窄の予防等の処置を行うために使用するステントである。デリバリーシステムにより閉塞部へ送達される。なお、本品は滅菌済みディスポーザブル製品のため、１回限りの使用でステントの再装填、再使用はできない。

外観：

<https://www.bostonscientific.com/jp-JP/products/stent/HANAROSTENT_Biliary_Full_Cover_NEO.html>より引用

②商品名：胆道用 BONASTENT カバー付き

製造販売会社名：メディコスヒラタ社

使用目的又は効果：

本品は悪性腫瘍により狭窄した胆管内へ経内視鏡的に挿入され、狭窄部の拡張を維持し、ドレナージを図るためのステントとステントを挿入するためのデリバリーシステムからなる。

外観：

<http://www.medicoshirata-products.com/article.php?p_id=36&b_id=5>より引用

## **3.2. EGBSの際に用いるデバイス**

本研究でEGBSの際に用いるデバイスは以下の2つである。尚、Through＆Pass Double Pigtailのステント径は7Fr、IYO-stentのステント径は5Frであり、FCSEMS留置後の胆嚢炎発症予防の観点からの有効性に差異が生じる可能性がある。そのため8.2.項で後述する様に、副次評価項目としてステント毎のサブグループ解析を実施する。

1. 商品名：Through＆Pass　Double Pigtail

製造販売会社名：GADELIUS社

使用目的又は効果：

本品は、長期的使用を目的として経内視鏡的に胆道（胆のう、胆のう管および胆管系）、膵管に挿入し、排のう、排液、狭窄部位の拡張・支持、狭窄の予防の処置を行う際に用いるチューブステント及びステントシステムである。

外観：

<https://www.gadeliusmedical.com/ja/products/Catheters/drainage.html>より引用

1. 商品名：胆嚢用IYO-stent

製造販売会社名：GADELIUS社

使用目的又は効果：

本品は、長期的使用を目的として経内視鏡的に胆道（胆のう、胆のう管および胆管系）、膵管に挿入し、排のう、排液、狭窄部位の拡張・支持、狭窄の予防の処置を行う際に用いるチューブステント及びステントシステムである。

外観：

 <https://www.gadeliusmedical.com/ja/products/Catheters/ps.html>より引用

# **4. 本研究で用いる規準・定義**

遠位胆管の定義は、胆道癌取扱い規約第8版に従う。

急性胆嚢炎の診断基準、重症度評価は「急性胆管炎・胆嚢炎診療ガイドライン2018」に記載された急性胆嚢炎の診断基準[18]に基づいて行う。確診症例のみを胆嚢炎と診断し、疑診となる症例は胆嚢炎として扱わない。これは胆嚢炎を発症していなくても、腫瘍による腹痛および血液検査での白血球、CRPの上昇のみで疑診に該当してしまうためである。

# **5. 研究対象者の選定方針**

## **5.1. 選択基準**

(1) 登録時年齢が18歳以上の患者

(2) 膵癌、遠位胆管癌、十二指腸乳頭部癌、癌種は問わずリンパ節転移など、悪性腫瘍で遠位胆管狭窄による閉塞性黄疸を来していると診断された患者

(3) 胆嚢摘出術の既往がない患者

(4) 4か月以上の予後が見込まれる患者

(5) 本研究の参加に関して本人又は代諾者^※^から同意が文書で得られる患者

※代諾者による同意が許容される状況に関しては「13.2. 代諾者等からインフォームド・コンセントを受ける場合」で詳細を記した。

## **5.2. 除外基準**

(1) PTGBD、EUS-GBD、EGBSによる胆嚢ステント留置による胆嚢ドレナージが登録時に既に行われている患者
(2) BillrothⅠ法以外の術後再建腸管のため、側視鏡を用いたERCP処置が困難な患者。
(3) ERCP施行前のCTおよび／またはMRIにおいて、①胆嚢管開口部を明瞭に同定でき、かつ②留置予定のFCSEMSの肝門側端が胆嚢管開口部より十二指腸側に位置し、胆嚢管開口部と重ならないことが予想される患者。①もしくは②のいずれかの条件を判断できない場合には、本基準による除外は行わない。悪性胆管狭窄部の肝門側端から胆嚢管開口部までの距離について、一律のカットオフ値は設定しない。画像評価は各研究機関の経験豊富な内視鏡医2名が独立して行い、両者の判断が一致した場合に限り除外する。
(4) 腫瘍の十二指腸浸潤による消化管狭窄により、ERCPが実施困難な患者
(5) 上部消化管穿孔を来しており、ERCPが禁忌となる患者
(6) 妊娠中，妊娠している可能性がある、または治療期間中に妊娠を希望する患者
(7) FCSEMS留置30日後の担当医による対面でのフォローアップが困難と予想される患者

(8) 本試験と同時又は本試験への組み入れ前30日以内に医薬品または医療機器等の治験あるいは臨床試験に参加した者

(9) その他，医師の判断により対象として不適当と判断された患者

# **6. 研究計画**

## **6.1. 研究デザイン**

本試験は前向きの単群での探索的試験である。

## **6.2. 目標症例数**

研究全体の目標症例数：50例

症例数の設定根拠は「9.2.目標症例数の設定根拠」参照

## **6.3. 研究期間**

研究期間：研究機関の長の実施許可日から2030年3月31日まで（5年間）

登録期間：研究機関の長の実施許可日から2028年3月31日まで

追跡期間：2028年4月30日

## **6.4. 割付方法**

**6.4.1. 割付方法と割付調整因子**

本研究では割付を実施しないため、該当しない。

**6.4.2. 盲検化の方法**

本研究では盲検化しないため、該当しない。

**6.4.3. 開鍵（キーオープン）の必要性の判断及び手順**

本研究では盲検化しないため、該当しない。

## **6.5. 治療計画**

**6.5.1. プロトコル治療**

**6.5.1.1. FCSEMS＋EGBS群**

登録後30日以内に、悪性遠位胆管狭窄に対してERCP下にFCSEMSを留置する際、処置後の胆嚢炎の発症予防を目的としてEGBSを試みる。

まず内視鏡を十二指腸まで挿入し、十二指腸乳頭部より胆管にガイドワイヤーを挿入する。次いでガイドワイヤーに沿ってカテーテルを挿入し、胆道造影を行い、腫瘍による遠位胆管狭窄を確認するとともに、胆嚢管開口部の同定を試みる。必要に応じて内視鏡的乳頭括約筋切開術を加える。

胆道造影後、胆嚢管開口部が造影で確認できたか否かにかかわらず、胆嚢管開口部またはその推定位置に向けて、胆嚢管を経由して胆嚢内へガイドワイヤーを進めることを目的とした意図的なガイドワイヤー操作を行う。この意図的なガイドワイヤー操作を開始した時点を「EGBSの試行」と定義する。胆嚢管開口部を造影で確認したのみで、同部位またはその推定位置に向けたガイドワイヤー操作を行わなかった場合は、EGBSの試行とはみなさない。

ガイドワイヤーを胆嚢内まで進めることに成功した場合は、胆嚢と十二指腸が交通するように胆嚢ステントを留置する。胆嚢ステントには、あらかじめ規定した7-Fr Through & Pass Double Pigtail stentまたは5-Fr IYO-stentを使用する。使用するステントの種類および口径は、胆道造影で確認した胆嚢管の走行および径を踏まえて術者が判断し、実際に留置したステントの種類および口径を症例ごとに記録する。

内視鏡挿入後60分を経過してもEGBSが成功しない場合は、EGBSの試行を中止する。ただし、術者がそれ以前に手技の継続を技術的に困難または安全でないと判断した場合は、60分を待たずに試行を中止する。この60分は内視鏡挿入からの経過時間を指し、胆管挿管、胆道造影および必要に応じて実施する内視鏡的乳頭括約筋切開術など、EGBS試行前に行う通常のERCP処置時間を含む。したがって、EGBSに関連するガイドワイヤー操作のみを60分間継続することを意味するものではない。

総処置時間は、内視鏡挿入から抜去までの時間とする。EGBS試行時間は、胆嚢管開口部またはその推定位置に向けた意図的なガイドワイヤー操作の開始から、胆嚢ステントの留置成功またはEGBS試行の中止までの時間とする。FCSEMS留置時間は、胆管内に留置したガイドワイヤーに沿ってFCSEMSデリバリーシステムの挿入を開始してから、FCSEMSの展開を完了するまでの時間とする。

EGBSの成否にかかわらず、遠位胆管狭窄部にHANAROSTENT Biliary Full Cover NEOまたはBONASTENTカバー付を留置し、処置を終了する。使用したFCSEMSの種類を症例ごとに記録する。EGBSの試行に使用したすべてのガイドワイヤーおよびカテーテルについても、製造販売会社名および製品名を症例ごとに記録する。

一連の処置は入院下で行う。また術者および介助者は、既報[19]も参考に、いずれも術者として 200件以上のERCP経験を有する内視鏡医に限定する。処置後に合併症を認めない場合、入院期間はおおむね1週間を想定する。

追跡期間中に急性胆嚢炎を発症した場合は、「急性胆管炎・胆嚢炎診療ガイドライン2018」[18]に準じて、各施設ごとに対応し、実施した治療内容を記録する。

**6.5.1.2. 対照群**

本研究は単群での探索的研究あり、対照群は存在しない

**6.5.2. 投与基準**

**6.5.2.1. 投与開始基準**

研究を目的とした薬剤投与は本研究では行わないため、該当しない。

**6.5.2.2 減量・休薬基準**

研究を目的とした薬剤投与は本研究では行わないため、該当しない。

**6.5.2.3再開基準**

研究を目的とした薬剤投与は本研究では行わないため、該当しない。

**6.5.3. 併用薬・併用療法**

- 項目名の“○○投与群”は実際の薬剤名等に変更する等適切に修正する。
- 登録後何日以内に試験治療薬の投与を開始する必要があるのかを規定する。
- 試験治療薬の用法・用量、投与期間を明記する。
- 遵守すべき事項を記載する（入院が必要な場合は入院期間など）。

**6.5.3.1. 併用禁止治療**

　　該当なし

**6.5.3.2. 併用注意治療**

該当なし

**6.5.3.3. 併用可能治療**

該当なし

**6.5.4. 個々の研究対象者における研究の中止**

**6.5.4.1. 中止基準**

以下の基準のいずれかに該当する場合は、研究対象者の研究を中止する。

（1）患者または代諾者から同意撤回の申し出があった場合

（2）登録後に選択基準に合致しない又は除外基準に抵触し対象として不適切であることが判明した場合

（3）登録後、疾患の症状、所見の悪化によりFCSEMSの留置が困難となった場合

（4）登録後、FCSEMS留置を目的としたERCP前に急性胆嚢炎を発症し、PTGBD、EUS-GBD、胆嚢摘出術による処置が実施された場合

（5）研究計画書からの重大な逸脱が発生した場合

（6）死亡

（7）転院等の理由により、追跡調査の継続が困難となった場合

（8）妊娠が判明した場合

（9））その他、研究の継続が好ましくないと研究責任者又は研究分担者が判断した場合

**6.5.4.2. 中止手順**

（1）研究責任者又は研究分担者は、研究期間中に研究対象者へのFCSEMS留置の際のEGBSを予め行わないと決定する場合、当該研究対象者に速やかにその旨を説明し、必要な観察、検査及び評価を行う。

（2）ERCPでFCSEMSを留置する際のEGBSを予め中止した日付と理由を症例報告書に記載する。

**6.5.4.3. プロトコル治療終了（中止）後の後治療**

本研究では該当しない。

# **7. 観察・検査・調査・評価項目**

## **7.1. スケジュール表**

| 時　期  項　目 | | スクリーニング  期間 | ERCP |  | | 後観察  期間 | 中止時 |
| --- | --- | --- | --- | --- | --- | --- | --- |
|  |  |  | Day0 | Day1 | Day30 | 2028/04/30^*3^まで |  |
| 許容範囲（日） | | -30～-1 | 0 | 0 | ＋10 | ±14 | ±7 |
| 同意取得 | | ○ |  |  |  |  |  |
| 患者背景の確認 | | ○ |  |  |  |  |  |
| プロトコル治療 | |  | ○ |  |  |  |  |
| 自覚症状・他覚所見 | | ○ | ○ | ○ | ○^*2^ |  |  |
| バイタルサイン | | ○ | ○ | ○ |  |  |  |
| 臨  床  検  査 | 血液学的検査 | ○ |  | ○ | ○ |  |  |
|  | 血液生化学検査 | ○ |  | ○ | ○ |  |  |
| 画像検査（CT）^*1^ | | ○ |  |  |  |  |  |
| 有害事象の観察 | |  |  |  |  |  |  |

※1 研究登録前30日以内にCTの撮像歴がない場合は、登録前60日以内のCTでも代用可能とする。

※2　FCSEMS留置後の胆嚢炎発症の有無の確認は、研究対象者と対面で確認する事が望ましいが、対面での確認が難しい場合は電話での確認も許容する。

※3　2028年4月30日（±14日）時点で、FCSEMS留置後の胆嚢炎発症の有無を電話もしくは診察で確認する。この期間に確認が難しい場合は、それ以前の最終診察日までの期間を追跡期間とする。

## **7.2. 実施スケジュール時期と評価項目**

7.2.1. スクリーニング期間

(1)研究対象者背景

・性別、年齢、身長、体重、既往歴、合併症

・同意取得日

・悪性遠位胆管狭窄の診断日および、原疾患、胆嚢結石の有無、胆嚢管開口部への腫瘍浸潤の有無

(2)自覚症状・他覚所見

急性胆嚢炎の主要症状を中心に身体所見をとる

(3)バイタルサイン

・血圧、脈拍数、体温

(4)血液学的検査

・白血球数、赤血球数、ヘマトクリット、ヘモグロビン、血小板

(5)生化学的検査

・血清アルブミン、総ビリルビン、直接ビリルビン、AST、ALT、LDH、CRP、ALP、γ-GTP、AMY、BUN、血清クレアチニン、Na、K、Cl

(6)CT検査

7.2.2. Day0

(1)自覚症状・他覚所見

急性胆嚢炎の主要症状を中心に身体所見をとる

(2)バイタルサイン

・血圧、脈拍数、体温

(3)有害事象の評価

　　　　　　自覚症状、他覚所見等から有害事象が想定される場合は血液検査や画像検査の実施を検討する

7.2.3. Day1

(1)自覚症状・他覚所見

急性胆嚢炎の主要症状を中心に身体所見をとる

(2)バイタルサイン

・血圧、脈拍数、体温

(3)血液学的検査

・白血球数、赤血球数、ヘマトクリット、ヘモグロビン、血小板

(4)生化学的検査

・血清アルブミン、総ビリルビン、直接ビリルビン、AST、ALT、LDH、CRP、ALP、γ-GTP、AMY、BUN、血清クレアチニン、Na、K、Cl

　　　　(5)有害事象の評価

　　　　　　自覚症状、他覚所見等から有害事象が想定される場合は血液検査や画像検査の実施を検討する

7.2.4. Day30

(1)自覚症状・他覚所見

急性胆嚢炎の主要症状を中心に身体所見をとる

(2)血液学的検査

・白血球数、赤血球数、ヘマトクリット、ヘモグロビン、血小板

(3)生化学的検査

・血清アルブミン、総ビリルビン、直接ビリルビン、AST、ALT、LDH、CRP、ALP、γ-GTP、AMY、BUN、血清クレアチニン、Na、K、Cl

　　　　(4)有害事象の評価

　　　　　　自覚症状、他覚所見等から有害事象が想定される場合は血液検査や画像検査の実施を検討する

7.2.5. 後観察期間　（2028/04/30まで）

(1)胆嚢炎発症の有無の確認

　　　　(2)有害事象の評価

　　　　　　自覚症状、他覚所見等から有害事象が想定される場合は血液検査や画像検査の実施を検討する

## **7.3. 評価の方法**

CT検査の撮影条件は以下とする。

・撮像範囲：総胆管の評価目的に上腹部が含まれている事を条件とする。

・スライス厚は5mm以下とする。

・造影剤の使用：造影剤は原則使用するが、アレルギーがある場合は単純撮影も可能とする。

# **8. 評価項目**

## **8.1. 主要評価項目**

・EGBSを試みた全症例での、FCSEMS留置後30日以内の胆嚢炎発症割合

　　　　【測定方法および各イベントの定義】

胆嚢炎の診断は「急性胆管炎・胆嚢炎診療ガイドライン2018」に記載された急性胆嚢炎の診断基準[18]に基づいて行う。確診となる場合のみ胆嚢炎と診断し、疑診となる症例は胆嚢炎として扱わない。胆嚢炎と診断した場合は、「急性胆管炎・胆嚢炎診療ガイドライン2018」に基づき重症度の評価も行う。評価者は各施設の研究責任者が行う。FCSEMS留置翌日に、血液検査および身体診察による評価を行う。以降も発熱や腹痛などが出現した場合は、診察を行い、胆嚢炎発症の有無の評価を行う。FCSEMS留置後30日以内に、急な転院などやむを得ない事情により、担当医による対面でのフォローアップが難しい場合は、電話による聞き取り調査で腹痛や発熱が無かったか確認する。

FCSEMS留置後30日以内に急性胆嚢炎を発症した場合を、主要評価項目のイベントとする。急性胆嚢炎に対して胆嚢摘出術または胆嚢ドレナージが実施された場合も、主要評価項目のイベントとして扱う。

FCSEMS留置後30日以内に急性胆嚢炎を発症することなく死亡した場合、または急性胆嚢炎以外の適応で胆嚢摘出術もしくは胆嚢ドレナージが行われた場合は、イベントとはせず分母に含める。該当する研究対象者数、事象の時期および理由を別途報告する。

【設定根拠】

本研究の目的は悪性遠位胆管狭窄を来した症例に対してFCSEMSを留置する際に、EGBSを行うことで術後の胆嚢炎の発症割合を下げることができるのか探索的に検討することである。そのため主要評価目的をFCSEMS留置後の胆嚢炎発症割合と定めた。既報ではFCSEMS留置後の胆嚢炎の約8割は30日以内に集積すると報告されている[2]ことを参考とし、30日以内の胆嚢炎発症割合と定めた。

## **8.2. 副次評価項目**

・EGBSの成功率

FCSEMS留置前に、胆嚢にThrough＆Pass　Double PigtailもしくはIYO-stentを留置する事ができた場合をEGBSの成功と定義する。算出方法は、「EGBSが成功した被験者数/EGBSを試みた被験者数」とする。

・EGBSに関連した有害事象の発生割合

本研究において実施された内視鏡処置に関連して発生した有害事象を対象とし、10.2項に基づいて評価する。算出方法は「内視鏡処置関連有害事象が1件以上発生した研究対象者数／SASに含まれる研究対象者数」とする。有害事象の種類、重症度およびプロトコル治療との因果関係についても記録する。

・EGBS成功例でのSEMS留置後の胆嚢炎発症割合

胆嚢炎の診断および測定方法は主要評価項目と同様である。算出方法は「EGBSが成功しFCSEMSを留置した後、胆嚢炎を発症した被験者数/EGBSが成功しFCSEMSを留置した被験者数」とする。また「Through＆Pass Double pigtail」、「胆嚢用IYO-stent」留置例それぞれで胆嚢炎の発症割合も測定する。

・FCSEMS留置後31日以降の胆嚢炎の累積発生率

胆嚢炎の診断および測定方法は主要評価項目と同様である。FCSEMS留置後31日目を起点とし、31日時点で生存かつ追跡中であり、それ以前に確診急性胆嚢炎を発症していない研究対象者を対象として、死亡等の競合事象を考慮した累積発生率を評価する。

- 本研究で最も重要な価項目を1つ記載すること。またその設定根拠も記載すること。
- 設定した主要評価項目の定義および算出方法を記載すること。

# **9. 統計解析**

## **9.1. 解析対象集団**

解析対象集団は、以下のように定義する。主たる解析対象集団はFASとする。

**9.1.1. 最大解析対象集団（full analysis set：FAS）**

本研究に登録され、EGBSの試行が開始され、かつ処置後の転帰情報が少なくとも1つ得られたすべての研究対象者を、EGBSの技術的成否およびその後の治療にかかわらずFASに含める。登録後にEGBSの試行に至らなかった研究対象者、処置後の転帰情報が全く得られなかった研究対象者、ベースラインデータが取得できなかった研究対象者、および重大な研究計画書違反が認められた研究対象者はFASから除外する。除外された研究対象者の人数および理由を報告する。

**9.1.2. 研究計画書に適合した対象集団（per protocol set：PPS）**

FASから、選択基準または除外基準に関する重大な研究計画書違反が認められた研究対象者を除いた集団をPPSとする。

**9.1.3. 安全性解析対象集団（safety analysis set：SAS）**

本研究に登録され、プロトコルで規定された内視鏡処置（FCSEMS留置およびEGBSの試行を予定して実施するERCP）が開始されたすべての研究対象者をSASとする。

**9.1.4．解析対象集団の設定根拠**

FASは、FCSEMS留置時にEGBSを試みるという治療戦略の転帰を、EGBSの技術的成否にかかわらず評価するために設定する。したがって、EGBSの技術的不成功例は、処置後の転帰情報が得られている限りFASに含める。

一方、EGBSの試行に至らなかった研究対象者および処置後の転帰情報が全く得られなかった研究対象者は、本研究の治療戦略または処置後転帰を評価できないためFASに含めない。PPSを用いた解析は補足的解析として実施する。PPSを実施する目的は、よりプロトコルに厳密だった集団に対して、本試験が想定する環境下での効果に関心があるためである。

## **9.2. 目標症例数の設定根拠**

既報では、EGBSを試みない場合のFCSEMS留置後30日以内の胆嚢炎発症割合が12.1%（41/339）[2]、EGBSを試みた場合のSEMS留置後30日以内の胆嚢炎発症割合が3.1%（1/32）[15]と報告されている。本研究の主要評価項目は3%と仮定する。4施設の参加施設における2年間の登録期間から想定される登録被験者数は54例であることから目標登録数を50例とする。中止や逸脱が10%あることを考慮した際に、45例におけるWilson Score を用いた90％信頼区間は0.8-10.5%の範囲と算出された。

## **9.3. 統計解析方法**

**9.3.1. 主要評価項目の解析**

**9.3.1.1 主要な解析**

FASを対象として、FCSEMS留置後30日以内に急性胆嚢炎を発症した研究対象者の頻度および割合を算出し、Wilson score法による両側90％信頼区間を算出する。

30日以前に追跡不能となり主要評価項目を判定できない場合は欠測として扱い、発症割合を算出する際の分母から除外する。欠測値の補完は行わず、該当する研究対象者数、追跡期間および追跡不能の理由を報告する。

**9.3.1.2. 副次的な解析**

先行研究との探索的比較として、FCSEMS留置後30日以内の急性胆嚢炎発症割合について、帰無仮説の母比率を12.1％とする両側有意水準10％の1標本母比率検定を実施する。本検定は将来の比較試験の計画に資するための探索的解析であり、その結果のみから予防的EGBSの因果的な治療効果を結論づけない。

**9.3.1.3．補足解析**

主要評価項目について、Wilson score法による両側95％信頼区間も算出する。また、PPSを対象として主要解析と同様の解析を実施する。

使用したFCSEMSの種類別の発症割合についても探索的に集計するが、正式な統計学的比較は行わない。

**9.3.2. 副次評価項目の解析**

**9.3.2.1．EGBSの技術的成功率**

SASのうち、6.5.1.1項に定義するEGBSの試行が開始された研究対象者を対象として、EGBSの技術的成功例の頻度および割合を算出し、Wilson score法による両側90％信頼区間を算出する。

**9.3.2.2．内視鏡処置関連有害事象**

SASを対象として、内視鏡処置関連有害事象が1件以上発生した研究対象者の頻度および割合を算出し、Wilson score法による両側90％信頼区間を算出する。有害事象の種類、重症度およびプロトコル治療との因果関係についても記述的に集計する。

**9.3.2.3．EGBS技術的成功例における30日以内の急性胆嚢炎**

EGBSが技術的に成功した研究対象者を対象として、FCSEMS留置後30日以内の急性胆嚢炎の頻度および割合を算出し、Wilson score法による両側90％信頼区間を算出する。留置した胆嚢ステントの種類および口径別の発症割合についても探索的に集計する。

**9.3.2.4．FCSEMS留置後31日目以降の急性胆嚢炎**

FCSEMS留置後31日時点で生存かつ追跡中であり、それ以前に確診急性胆嚢炎を発症していない研究対象者を対象として、31日目を起点とするランドマーク時間‐イベント解析を実施する。確診急性胆嚢炎の発症前の死亡、ならびに確診急性胆嚢炎以外の適応で実施された胆嚢摘出術または胆嚢ドレナージを競合事象として扱う。Aalen–Johansen法を用いて確診急性胆嚢炎の累積発生曲線を推定し、ランドマーク時点から観察終了までの実観察期間の中央値を報告する。

**9.3.2.5．その他の副次評価項目**

急性胆嚢炎の重症度、治療内容、および使用したデバイスに関する項目は記述的に集計する。

## **9.4. 中間解析**

本研究では中間解析は実施しない。

# **10. 有害事象の取り扱いについて**

## **10.1. 有害事象の定義**

有害事象（Adverse Event：AE）とは，研究との因果関係の有無に関わらず研究対象者に生じた全ての好ましくない又は意図しない医療上の出来事であり、意図しない徴候、臨床検査値の臨床的に有意な変動、症状、合併症の悪化を含む。

本研究では治療開始日から後観察期間終了までの間に発生した事象を有害事象として取り扱い、重篤な有害事象および中等症以上の有害事象についてのデータを収集する。尚、研究責任者が原疾患の悪化に起因すると判断した事象は除外する。

## **10.2. 有害事象の評価**

AEの程度はERCP関連手技による事象については、Cottonらによって提唱されたERCP関連手技における主要な偶発症の定義とグレード分類[20]に準じ評価する。

・ERCP関連手技における主要な偶発症の定義

1. 穿孔：腸管外への造影剤や空気の流出を認める状態。
2. 穿通：穿孔には至っていないが、腸管外への意図しないデバイスの貫通を認めたもの。
3. デバイス陥頓：デバイスの除去が不可能な状態。
4. 出血：吐血または黒色便の出現、もしくは2g/dLを超えるヘモグロビン値の低下を呈する状態。
5. 胆管炎：胆汁のうっ滞を伴った、24時間以上持続する38度の発熱を呈する状態。
6. ERCP後膵炎：正常値の3倍以上のアミラーゼもしくはリパーゼ値の上昇を伴った、心窩部痛を呈する状態。

・ERCP関連手技における偶発症のグレード分類

1. 軽症

ERCP関連の手技が偶発症により中止された場合。

偶発症により他科への医療相談がなされた場合。

偶発症により3日以内の入院の延期が生じた場合。

1. 中等症

偶発症により4日以上10日以内の入院の延期が生じた場合。

偶発症により1泊以内のICUへの入室が生じた場合。

偶発症により輸血を要する場合。

偶発症に対して内視鏡処置、血管内治療を要する場合。

1. 重症

偶発症により11日以上の入院の延期が生じた場合。

偶発症により2泊以上のICUへの入室が生じた場合。

偶発症に対して外科手術を要する場合。

偶発症により患者に永続的な障害が生じた場合。

1. 致命的合併症

偶発症により患者が死亡した場合。

尚、上記入院期間の延長について、以下の入院は本研究では有害事象とみなさない。

1）同意書への署名以前に予定されていた待機的手術

2）健康状態とは関係のない事情による、内科的/外科的介入を必要としない入院・入院期間の延長（住居の欠如、経済的困窮、介護者の休息を目的としたレスパイトケア、家族的な事情等）

3）健康状態の評価のための入院（通常の大腸内視鏡検査等）

プロトコル治療との因果関係は以下の3つに分類する。

(1) 関連あり：プロトコル治療との因果関係は妥当で原疾患の増悪、合併症、他の治療などが原因でないと考えられる

(2) 関連が否定できない：プロトコル治療との因果関係は明らかでない。原疾患の増悪、合併症、他の治療などでも説明しうる

(3) 関連なし：プロトコル治療との因果関係がなく、原疾患の増悪、合併症、他の治療などで明らかに説明できる

## **10.3. 予期される有害事象等**

2.3.3.項でも記述した様に、Gosainらの報告では、EGBSを試みた19例のうち3例（15.8%）はガイドワイヤーが胆嚢管を穿通し、うち1例は緊急胆嚢摘出術を必要としたと報告している[16]。また国内のIshiiらの報告では, EGBSを試みた32例のうち1例（3.1%）でガイドワイヤーによる胆嚢管穿通が生じたと報告している[15].

## **10.4. 有害事象が発現した場合の措置**

**10.4.1. 研究対象者への措置**

研究責任者又は研究分担者は、有害事象が発生した場合、研究対象者の安全確保のため必要に応じ研究対象者に対し、治療及び試験薬投与の中止等、適切な措置を講じる。治療等が必要となった場合は、その旨を研究対象者に伝える。

研究責任者又研究分担者は、試験薬終了時の最終観察時点で有害事象が継続している場合は、それ以降もベースライン値の状態（Grade）に回復するまで、又は臨床的に安定するまで追跡調査を実施する。

**10.4.2. 評価及び記録**

研究責任者又は研究分担者は、発現した有害事象について、原資料（診療録等）に有害事象名、発現日、重篤・非重篤の別、処置・治療の内容、転帰（回復した場合は回復時期、症状が固定した場合はその時期）を記載する。

**10.4.3. 有害事象の報告**

有害事象の発生を知った場合は、研究責任者又は研究分担者は、症例報告書により、研究代表者（又は研究事務局）に報告する。研究責任者は、研究機関の手順書に従い、必要に応じて、研究機関の長等に当該有害事象を報告する。

**10.4.4. 重篤な有害事象の報告**

重篤な有害事象の発生を知った場合、当該機関で定められている手順に従い、研究分担者は研究責任者に速やかに報告する。研究責任者は、重篤な有害事象の発生を知った場合は、所属する研究機関の長へ報告した上で、速やかに、その旨を研究代表者（又は研究事務局）に報告する。また、研究分担者に対して、情報を共有する。

研究代表者は、有害事象の評価及びそれに伴う研究の継続の適否、研究の変更について倫理審査委員会の意見を聴き、必要な措置を講じる。

研究代表者（又は研究事務局）は、研究責任者より報告を受けた有害事象を、有害事象が発生した以外の研究機関の研究責任者に、可能な限り速やかに報告する。

研究代表者及び各研究機関の研究責任者は、研究機関の手順に従い、倫理審査委員会の意見を踏まえて所属する研究機関の長に報告するとともに、研究分担者に情報を共有する。

# **11. 効果安全性評価委員会**

本研究では効果安全評価委員会は設置しない。

# **12.研究参加に伴い研究対象者に予測される利益及び不利益等**

## **12.1. 予想される利益**

本研究参加により研究対象者に直接の利益は生じない。研究参加により将来の医療の進歩に貢献できる可能性がある。

## **12.2. 負担並びに予想されるリスク**

本研究で用いる使用機材はいずれも本研究の対象疾患に対して適応が承認され保険適用されているものであり、日常診療に比して、研究対象者が本研究に参加することで経済上の負担はない。本研究に参加する事で研究対象者に起こり得る有害事象は「10.3. 予期される有害事象等」に記載した。研究責任者又は研究分担者は、有害事象が発生した場合、「10.4. 有害事象が発現した場合の措置」に従い適切に対処する。

# **13. インフォームド・コンセントを受ける手続**

本研究は、侵襲・介入を伴い、研究対象者から本研究のために新たに試料・情報を取得して実施する研究である。人を対象とする生命科学・医学系研究に関する倫理指針第8の1（1）に該当する。

研究責任者および研究分担者は、患者が研究に参加する前に、「倫理審査委員会」（20.2.項参照）で承認された説明文書・同意書・同意撤回書を用いて以下の事項を十分に説明する。患者が内容を十分理解したことを確認した後、参加の同意を文書により取得する。

インフォームド・コンセントを受ける際に研究対象者等に対し説明すべき事項は、原則として以下のとおりとする。ただし、倫理審査委員会の意見を受けて研究機関の長が許可した事項については、この限りでない。

１． 研究の実施について許可を受けていること

２． 研究機関の名称及び研究責任者の氏名

３． 研究の目的及び意義

４． 研究の方法及び期間

５． 研究の対象者として選定された理由

６． 研究対象者に生じる負担並びに予測されるリスク及び利益

７． 健康被害に対する補償の有無及びその内容

８． 同意を撤回できること

９． 不利益を受けないこと

10．個人情報等の取り扱い

11．試料・情報の保管及び廃棄の方法

12．試料・情報の二次利用について

13．研究により得られた結果等の取り扱い

14．費用及び謝礼について

15．研究の資金源と利益相反

16．他の治療方法について

17．研究実施後の医療の提供に関すること

18．研究に関する情報公開の方法

19．資料を閲覧できること

20．関係者が試料・情報を閲覧すること

21．連絡お問い合わせ先

## **13.1. 研究対象者等及びその関係者からの相談等への対応**

研究対象者等やその関係者からの相談には、研究責任者または研究分担者が対応する。対応の方法が不明の場合には、相談内容に合わせて研究事務局と協議の上、対応する。

## **13.2. 代諾者等からインフォームド・コンセントを受ける場合**

本研究では、対象疾患の特性上高齢者が多く、判断能力を有さない患者も一定の割合で含まれる可能性がある。対象者本人にインフォームド・コンセントを与える能力がないと客観的に判断される場合は、倫理審査委員会で承認された説明・同意文書を用いて代諾者の同意を文書により取得する。代諾者の範囲は、研究対象者の配偶者、2親等以内の親族、同居の親族とする（未成年者を除く）。ただし研究対象者本人に対しても、その理解力に応じ、説明・同意文書の項目に従って内容を分かりやすい言葉で説明し、同意又は賛意を得るものとする。

## **13.3. インフォームド・アセントを得る場合**

本研究では成人患者を対象としているため該当しない。

## **13.4. 指針第8の8の規定による研究を実施しようとする場合**

本研究では該当しない。

# **14. 施設登録及び症例登録**

施設登録及び症例登録は，登録センター（担当部署：横浜市立大学附属病院　肝胆膵消化器病学教室）の中央登録制とする．施設登録が完了した研究機関から症例登録が可能となる．

## **14.1. 登録センター**

担当者：八木　伸

施設名：横浜市立大学附属病院　肝胆膵消化器病学教室

TEL：045-787-2800

FAX：045-787-2931

Mail：t216074f@yokohama-u.ac.jp

受付時間：　平日9時から16時（土日、祝祭日、年末年始は受付けない）

## **14.2. 施設登録**

各研究機関の研究責任者は，倫理審査委員会での承認（20.2.項参照）及び研究機関の長の実施許可（20.3.項参照）が得られた後、研究機関の長の許可書（写し）を登録センターに送付する。登録センターは施設登録を行い、研究責任者に施設登録完了通知を行う。

## **14.3. 症例登録**

（1）研究責任者又は研究分担者は、候補となる研究対象者から文書による同意を取得後、適格性を判断するために必要な検査等（スクリーニング）を実施する。研究対象者が選択基準を満たし、かつ除外基準のいずれにも該当していないことを確認する。

（2）研究責任者又は研究分担者は、症例登録票に必要事項を記載し登録センターにFAXもしくはe-mailで送信する。この際、各研究機関において研究対象者識別コードを付与するが、この研究対象者識別コードは単体で特定の個人を識別することができる情報を含まない任意のコードを用いる。

（3）登録センターは、症例登録票の記載内容から適格性を判定し、適格と判定した場合に本研究に組み入れる。

（4）登録センターは、登録確認票に研究への組み入れの可否を記載し、研究責任者又は研究分担者にFAXもしくはe-mailで送信する。

（5）研究責任者又は研究分担者は登録確認票を保存する。

# **15. 試料/情報の授受**

症例登録票と各症例報告書等は下表に記載されたとおりに提出する。

提出元：患者登録を行う共同研究機関（27.3.項）

症例登録票および症例報告書等の提出時期と提出方法

| 種類 | 提出時期 | 提出方法 | 安全管理 | 提出先 |
| --- | --- | --- | --- | --- |
| 症例登録票 | 症例登録時 | FAXもしくはe-mail | 送付先のダブルチェックを行う  e-mailの場合はパスワードで保護する | 登録センター（14.1項） |
| 治療前報告書 | 登録日から30日以内 |  |  | 研究事務局（27.2.項） |
| ERCP | 術後30日以内 |  |  |  |
| ERCP後30日 | 当該時期から30日以内 |  |  |  |
| 中止時 |  |  |  |  |
| 追跡調査票 | 2028年4月30日～  2028年5月31日 |  |  |  |

# **16. 試料・情報の保管及び廃棄の方法**

［情報の保管］

研究責任者は、下記に掲げる本研究に関する文書および記録を施錠可能な保管庫で厳重に保管管理する。電子データで保管する場合は、パスワードを設定した上で、院内LANやインターネットから独立したパソコンまたはUSBメモリ等の電磁的記録媒体にて保管し、使用していない時は施錠可能な保管庫で厳重に保管管理する。保管期間は、研究の終了について報告された日から5年間もしくは当該研究の結果の最終の公表について報告された日から3年を経過した日のいずれか遅い日までの期間とするが、個人が特定できないよう加工した情報については二次利用の可能性があるため保管期間終了後も期間を定めず保管する。

保管方法は情報の種類に応じて以下のとおり管理する。

保管する情報の種類

・研究対象者の個人が特定できる情報を含む情報（研究対象者の同定及び照会のため、研究対象者識別コードから研究対象者を特定することができる個人情報を管理する表（以下「対応表」という。）、同意書、同意撤回書等）

・研究対象者の個人が特定できないよう加工した情報

・研究に関する資料（研究計画書等の審査書類等）

それぞれ以下の通り保管する。

研究対象者の個人が特定できる情報について、電子データで情報を管理する場合は、各機関の研究責任者のもとで、記録メディアを保管庫で施錠管理し、紙媒体の情報を管理する場合は、保管庫で厳重に施錠管理する。また、同意書・同意撤回書については、紙媒体として原本保管するが、電子カルテにスキャンされた電子データは写しとして扱うものとする。

研究対象者の個人が特定できないよう加工した情報は、各機関の研究責任者のもとで、パスワードで管理されたパソコン/USBメモリ等の記録媒体にて保管する。

研究代表者へ集積した情報は、横浜市立大学附属病院　肝胆膵消化器病学教室で、パスワードで管理されたパソコン/USBメモリ等の記録媒体にて保管する。紙で提出を受けた症例報告書は、ファイルに綴じて保管庫で施錠管理する。

保管期間を経過した文書および記録は、個人情報や機密情報の漏洩がないように細心の注意を払い廃棄する。コンピュータ上にある情報は完全に削除し、紙媒体はシュレッダーにて裁断し廃棄する。（管理責任者：各機関の責任者）

1）研究計画書

2）説明文書・同意書・同意撤回書

3）症例登録票

4）同意書（署名ありの原本）

5）症例報告書（写）

6）対応表

7）倫理審査委員会へ提出した書類

8）倫理審査委員会の結果通知書および研究機関の長の実施許可書

9）モニタリング報告書

10）その他本研究に関連する文書又は記録

## **16.1. 試料・情報の二次利用について**

本研究で得られた試料・情報は、保管され、将来他の目的の研究に二次使用する可能性、国内外の研究機関・研究者等への第三者提供を行う可能性がある。その二次利用目的、提供先を現在特定することはできないが、研究対象者にはあらかじめその旨、同意取得時点において想定可能な範囲で説明し、同意を取得する。

本研究で得られた試料・情報を将来的に他の目的の研究に利用する際は、新たに研究計画書を作成し、倫理審査委員会の承認及び研究機関の長の許可を得る。そして、二次利用することについて文書での同意や本人への通知、もしくは情報公開文書の公告とともに拒否機会の保障を行ったうえで使用する。

# **17. 個人情報等の取扱い**

本研究では患者を登録する際に、研究対象者識別コードを付与する。研究対象者識別コードは、イニシャルやカルテID等のような特定の個人を識別できる情報とは無関係の数字記号等で構成され、症例登録票や症例報告書等の本研究に関する書類を作成する際には研究対象者識別コードを使用することで個人が特定できないよう加工を行う。研究責任者は、加工された情報から、必要に応じて研究対象者を識別することができるように研究対象者の氏名やカルテID等の情報が記載された対応表を作成し、外部に漏洩することがないよう厳重に保管管理を行う（管理責任者：各共同研究機関の研究責任者）。

本研究は横浜市立大学附属病院を含む4施設で実施する多機関共同研究であり、各研究機関で対応表を適切に管理することとし、研究機関外への提供は行わない。研究対象者の症例登録票、症例報告書、検査データ等を当該研究機関外に提供する際には、研究対象者識別コードまたは登録番号を用い、個人が特定できないよう加工する（どの研究対象者の試料・情報であるかが直ちに判別できないよう、加工又は管理されたものに限る）。本研究における個人情報等は、本研究計画書のほか、各研究実施機関における臨床研究に関する個人情報等の取扱いに関する手順書を遵守して取り扱う。

# **18. 研究対象者に係る研究結果（偶発的所見を含む．）の取扱い**

研究対象者個別における研究参加に伴う結果は、診療の中で研究対象者本人に説明する。。

本研究で研究対象者の健康、子孫に受け継がれ得る遺伝的特徴等に関する重要な知見が得られる可能性はないが、実施する検査等により、研究対象者の健康に重大な影響を与える情報（偶発的所見を含む）を入手した場合は、研究責任者または研究分担者が研究対象者に説明し、治療や処置を行う等の適切な措置を講じる。

研究対象者が、開示を希望しない旨の意思表示を行っている場合は開示しないが、その結果等が研究対象者、研究対象者の血縁者等の生命に重大な影響を与えることが判明し、かつ、有効な対処方法があるときは、研究責任者を通して研究代表者に報告する。研究代表者は、研究対象者等への説明に関して、説明の可否、方法及び内容について以下の観点を含めて考慮し、倫理審査委員会の意見を求める。

1. 研究対象者及び研究対象者の血縁者等の生命に及ぼす影響
2. 有効な治療法の有無と研究対象者の健康状態
3. 研究対象者の血縁者等が同一の疾患等に罹患している可能性
4. インフォームド・コンセントに際しての結果の説明に関する内容

研究責任者及び研究分担者は、倫理審査委員会の意見を踏まえ、研究対象者等に対し十分な説明を行ったうえで、研究対象者の意向を確認し、なお説明を希望しない場合には説明しない。

## **18.1.遺伝カウンセリングの体制**

本研究では遺伝子解析を行わないため、本項目は該当しない。

# **19. 研究計画書の遵守、逸脱及び変更について**

## **19.1. 研究計画書の遵守**

研究責任者又は研究分担者は、研究代表者の事前の合意及び倫理審査委員会の審査に基づく文書による事前の承認を得ることなく、研究計画書からの逸脱又は変更を行ってはならない。

## **19.2. 研究計画書からの逸脱**

1）研究責任者又は研究分担者は、研究対象者の緊急の危険を回避するためのものである等医療上やむを得ない場合には、研究代表者との事前の合意及び倫理審査委員会の事前の承認を得る前に、研究計画書からの逸脱あるいは変更を行うことができる。その際には、研究責任者又は研究分担者は、逸脱又は変更の内容及び理由並びに研究計画書の変更等が必要であればその案を速やかに、研究代表者を経由し倫理審査委員会に提出してその承認を得るとともに、研究機関の長の許可を得る。

2）研究責任者又は研究分担者は、研究計画書からの逸脱があった場合には、逸脱事項についてその理由とともに全て記録を作成し、研究責任者は研究機関の長及び研究代表者に報告しなければならない。研究責任者は、逸脱に関する記録の写しを保存する。

## **19.3. 研究計画書の変更手順**

研究計画書の内容を変更する場合は、以下の手順により行う。

1）研究代表者は、研究計画書の変更が必要と判断した場合は、研究責任者に研究計画書変更案及びその他必要な資料・情報を提供する。

2）研究代表者は、研究責任者が前項により提供された研究計画書変更案及びその他の資料・情報を十分検討し、研究代表者と協議するのに必要な時間を研究責任者に与える。

3）研究代表者は，研究責任者の合意が得られた後に、20.2.項、20.3項に従い変更の手続きを行う。

# **20. 研究実施に係る手続**

## **20.1 遵守すべき諸規則**

本研究は、ヘルシンキ宣言に基づいた倫理原則を遵守し、「人を対象とする生命科学・医学系研究に関する倫理指針」に従って実施する。研究責任者及び研究分担者は、本研究計画書を遵守してプロトコル治療を実施する。

## **20.2.倫理審査委員会への付議**

研究代表者は、本研究を実施する前に関係する研究機関と事前に調整を行った上で、一の倫理審査委員会に一括した審査を受け、承認を得る。また、研究計画書等の変更又は改訂を行う場合にも、最初に審査を依頼した倫理審査委員会による一括した審査を受け、承認を得る。

なお、中央一括審査を行わない共同研究機関は、研究代表機関の中央一括審査で承認された資料をもって当該機関の倫理審査委員会で審査を受け、承認を得る。当該機関の研究責任者は、当該機関における審査結果通知書を研究代表者へ提出し、審査結果の報告を行う。

## **20.3.研究機関の長による許可等**

研究代表者は中央一括審査結果、審査過程のわかる記録及び当該倫理審査委員会の委員の出欠状況、承認された資料を共同研究機関の研究責任者に共有し、各研究機関の研究責任者はそれをもって当該研究機関の長に研究の実施許可を得る。初回の審査に限らず、研究の変更について倫理審査委員会の審査を受けた際にはその都度、研究機関の長の許可を得るものとする。

なお、中央一括審査を行わない共同研究機関においては、当該機関の倫理審査委員会で承認された後、研究機関の長の許可を得るものとする。実施許可を得た際には、実施許可書を研究代表者へ提出し、報告する。

## **20.4.定期報告**

研究代表者及び各共同研究機関の責任者は、本研究の実施状況を少なくとも年1回又は倫理審査委員会の求めに応じてそれ以上の頻度で倫理審査委員会及び各研究機関の長に報告する。

## **20.5.研究の停止・中止**

研究者等は、研究の継続に影響を与えると考えられる情報を得た場合、当該機関の研究責任者に報告し、研究責任者は研究代表者及び自機関の長へ報告する。研究代表者は、自機関の長へ報告するとともに各研究機関の研究責任者へ情報を共有し、各研究機関の研究責任者は自機関の長へ報告する。

研究代表者は必要に応じて研究を停止若しくは中止し、又は研究計画書の変更を行う。また研究機関の長より研究の停止又は中止の指示を受けた場合は速やかに対応し、上記の手順で各研究機関と情報を共有し、各研究機関の研究責任者は当該機関の長へ報告する。

研究が中止または中断された場合、理由の如何を問わず、研究担当者は研究対象者に速やかにその旨を通知し、適切な処置ならびに研究対象者の安全を確認するための検査等を実施する。また、研究機関の長に文書で通知する。

（１）試験治療の有効性が期待できないことや安全性に対する問題が明らかとなった場合、または研究継続の意義が無くなったと判断された場合。

（２）症例登録の著しい遅れ、研究計画書からの逸脱の頻発などの理由により、研究の完遂が困難と判断された場合。

（３）研究の実施に影響を及ぼす重大な不適合事案が発生した場合。

## **20.6.研究の終了**

研究が終了（研究を中止し、再開の見込みがない場合を含む）したとき、研究代表者は、全ての研究責任者にその旨および研究結果の概要を文書で報告する。研究代表者及び各共同研究機関の研究責任者は、研究終了の旨および研究結果の概要を遅滞なく倫理審査委員会及び研究機関の長に報告する。

# **21.研究の資金源等，研究機関の研究に係る利益相反及び個人の収益等，研究者等の研究に係る利益相反に関する状況**

## **21.1. 資金源及び財政上の関係**

本研究は、横浜市立大学医学部、肝胆膵消化器病学教室の基礎研究費を財源として実施する。医学的な視点から行われ、特定の企業・団体の利益や便宜をはかるものではない。また、特定の企業・団体から資金援助は受けていない。

## **21.2. 利益相反**

**21.2.1. 研究代表者の利益相反**

　研究代表者である小田貴之には，開示すべき利益相反はない。

**21.2.2.研究責任者及び研究分担者等の利益相反**

研究責任者及び研究分担者の利益相反については、各研究機関の利益相反ポリシー及び規程等に従い各研究機関にて適切に管理を行う。利益相反の管理状況については、研究代表者に報告し、報告内容に変更が生じた場合にも報告する。

# **22.　研究対象者の費用負担・謝礼について**

本研究の実施にかかる費用のうち、個々の患者に要する医療費（診察費，入院費用，薬剤費，検査代など）については、本研究で行う治療がすべて保険診療範囲であることから自己負担分を研究対象者が支払う。本研究へ参加することで研究対象者に別途金銭的負担が増えることはない。本研究では、研究対象者に対して金銭的およびそれ以外による参加謝礼並びに負担軽減費等の支払い等はしない。

# **23. 健康被害に対する補償**

本研究に参加したことにより（起因して）研究対象者に健康被害が生じた場合、研究責任者及び研究分担者は、適切な治療及びその他必要な措置を行う。この場合の治療等は保険診療として行い、自己負担分の医療費を研究対象者が支払う。

本研究は、市販の医薬品の効能・効果，用量・用法、その他の注意事項等について添付文書に記載された範囲内で使用するため、医薬品の副作用により、死亡又は後遺障害1、2級の健康障害が生じた場合は、医薬品副作用救済制度による救済給付申請の対象となる。また、本研究で行う検査は、通常診療で行う検査と同じである。以上のことから、本研究においては臨床研究賠償責任保険への加入は行わない。

# **24. モニタリング及び監査**

## **24.1. モニタリング**

モニタリングとは、研究が適正に行われることを確保するため、研究がどの程度進捗しているか並びに研究計画書や各手順書及び関連法規、関連する指針に従って行われているかについて、研究責任者が指定したものに行わせる調査をいう。

(1) モニタリング方法と担当者

本研究ではデータセンターによる中央モニタリングを行う。また各施設において自己点検を行い、適格性や同意書関連の確認が実施されたことを記録する。

(2) 中央モニタリング
1) 中央モニタリングは症例報告書により収集されたデータを基に実施する。
2) 中央モニタリングの結果、記載されたデータの内容確認（誤記が疑われる場合や欠損データの追加・記入などに関して）クエリを発行し、回答を得るが、回答の結果必要があれば当該施設担当者（研究者やCRCなど研究補助者）に連絡を取る。その連絡方法は電話、FAX、webカンファレンスなどで行うが、どの方法であっても被験者の個人情報がマスキングされた状態で行う。
3) モニタリングの結果については「モニタリング報告書」に記載する。
(3) モニタリング内容

1)被験者登録状況、同意取得状況

2)被験者登録の妥当性

3)背景因子、治療の集計

4)研究計画書遵守状況

5)症例報告書の記載内容の信頼性

6)有害事象に関する適切な記録と報告

7)公開データベースの登録・終了状況

(4) モニタリング報告書の提出

データセンターはモニタリングを実施した結果を「モニタリング報告書」に記載し、研究責任者に実施後2週以内に提出する。実施の頻度は1年に1回とする。

## **24.2. 監査**

本研究では、監査を実施しない。

# **25. 研究の情報公開及び結果公表**

## **25.1. 研究の登録**

本研究の実施に先立ち、厚生労働省が整備するデータベース（jRCT = Japan Registry of Clinical Trials）に登録する。研究計画書の変更及び研究の進捗に応じて適宜更新する。研究を終了したときは、研究の結果を登録する。

## **25.2. 研究結果の公表**

研究終了後、本研究から得られた成果を速やかに学会発表あるいは論文投稿によって公表する。公表する際は、研究対象者等及びその関係者の人権又は研究者等及びその関係者の権利利益の保護のために必要な措置を講じた上で公表する。学会発表者及び論文の筆頭著者は協議の上決定する。発表者及び筆頭著者は、発表前及び投稿前に、研究代表者の審査・承認を受ける。最終の公表を行った場合は、その旨を全ての研究機関の長に報告する。

# **26．研究成果の帰属（知的財産権）**

本研究の結果として、特許権等の知的財産権が生じた場合、その権利は原則として公立大学法人横浜市立大学に帰属する。

# **27. 研究の実施体制**

## **27.1. 研究代表者**

研究機関名：　横浜市立大学附属病院

研究機関住所: 〒236-0004 神奈川県横浜市金沢区福浦3-9

研究機関TEL：　045-787-2800

研究代表者名：　小田　貴之

研究代表者所属： 横浜市立大学附属病院　肝胆膵消化器病学教室

役割：研究の立案、総括

## **27.2. 研究事務局**

氏名：　小田　貴之

所属：　横浜市立大学附属病院　肝胆膵消化器病学教室

住所：　〒236-0004 神奈川県横浜市金沢区福浦3-9

TEL：　045-787-2800

役割：各共同研究機関との連絡調整を行う。

## **27.3. 共同研究機関および研究責任者**

　　　１）患者登録を行う共同研究機関

　　　　役割：患者登録、プロトコル治療の実施

| 研究機関 | 機関の長 | 所属 | 研究責任者 |
| --- | --- | --- | --- |
| 独立行政法人 国立病院機構 横浜医療センター  〒245-8575 神奈川県横浜市戸塚区原宿3-60-2  045-851-2621（代表） | 宇治原　誠 | 消化器内科 | 二瓶　真一 |
| 茅ヶ崎市立病院  〒253-0042 神奈川県茅ケ崎市本村5-15-1  0467-52-1111（代表） | 藤浪　潔 | 消化器内科 | 佐藤　高光 |
| 独立行政法人 労働者健康安全機構 横浜労災病院  〒222-0036 神奈川県横浜市港北区小机町3211  045-474-8111（代表） | 三上　容司 | 消化器内科 | 鈴木　雅人 |

## **27.4.　研究協力機関と責任者**

　　該当なし

## **27.5. 統計解析責任者**

氏名：　篠田　覚

所属：　横浜市立大学医学部　臨床統計学教室

住所：　〒236-0004 神奈川県横浜市金沢区福浦3-9

E-mail： secbios@yokohama-cu.ac.jp

役割： 統計解析

## **27.6. データセンター/データマネージメント責任者**

氏名：栗田　裕介

所属：　横浜市立大学附属病院　肝胆膵消化器病学教室

住所：　〒236-0004 神奈川県横浜市金沢区福浦3-9

TEL：　045-787-2800

役割:　データマネジメント

## **27.7. モニタリング責任者**

氏名：　小林　奈保

所属：　横浜市立大学附属病院　肝胆膵消化器病学教室

住所：　〒236-0004 神奈川県横浜市金沢区福浦3-9

TEL：　045-787-2800

役割:　モニタリング管理

## **27.8. 監査責任者**

該当なし

## **27.9. 外部委託機関**

該当なし

# **28. 文献**

1.	国立研究開発法人国立がん研究センター. *がん情報サービス*. 2020 10 November 2024]; Available from: https://ganjoho.jp/reg_stat/statistics/stat/summary.html.

2.	Ishii, T., et al., *Risk factors for early and late cholecystitis after covered metal stent placement for distal biliary obstruction.* J Hepatobiliary Pancreat Sci, 2023. **30**(10): p. 1180-1187.

3.	Nakai, Y., et al., *International consensus statements for endoscopic management of distal biliary stricture.* J Gastroenterol Hepatol, 2020. **35**(6): p. 967-979.

4.	Inamdar, S., et al., *Comparison of Adverse Events for Endoscopic vs Percutaneous Biliary Drainage in the Treatment of Malignant Biliary Tract Obstruction in an Inpatient National Cohort.* JAMA Oncol, 2016. **2**(1): p. 112-7.

5.	Wang, D.H., et al., *Comparative Analysis of ERCP and PTBD for Biliary Interventions for Readmission Rates and Patient Outcomes.* J Clin Gastroenterol, 2024.

6.	Paik, W.H., et al., *EUS-Guided Biliary Drainage Versus ERCP for the Primary Palliation of Malignant Biliary Obstruction: A Multicenter Randomized Clinical Trial.* Am J Gastroenterol, 2018. **113**(7): p. 987-997.

7.	Bang, J.Y., et al., *Stent placement by EUS or ERCP for primary biliary decompression in pancreatic cancer: a randomized trial (with videos).* Gastrointest Endosc, 2018. **88**(1): p. 9-17.

8.	Park, J.K., et al., *Efficacy of EUS-guided and ERCP-guided biliary drainage for malignant biliary obstruction: prospective randomized controlled study.* Gastrointest Endosc, 2018. **88**(2): p. 277-282.

9.	Kaassis, M., et al., *Plastic or metal stents for malignant stricture of the common bile duct? Results of a randomized prospective study.* Gastrointest Endosc, 2003. **57**(2): p. 178-82.

10.	Davids, P.H., et al., *Randomised trial of self-expanding metal stents versus polyethylene stents for distal malignant biliary obstruction.* Lancet, 1992. **340**(8834-8835): p. 1488-92.

11.	Dumonceau, J.M., et al., *Biliary stenting: indications, choice of stents and results: European Society of Gastrointestinal Endoscopy (ESGE) clinical guideline.* Endoscopy, 2012. **44**(3): p. 277-98.

12.	Isayama, H., et al., *A prospective randomised study of "covered" versus "uncovered" diamond stents for the management of distal malignant biliary obstruction.* Gut, 2004. **53**(5): p. 729-34.

13.	Martinez, N.S., et al., *Evaluation of post-ERCP pancreatitis after biliary stenting with self-expandable metal stents vs. plastic stents in benign and malignant obstructions.* Endosc Int Open, 2021. **9**(6): p. E888-E894.

14.	Jung, J.H., et al., *Identification of risk factors for obstructive cholecystitis following placement of biliary stent in unresectable malignant biliary obstruction: a 5-year retrospective analysis in single center.* Surg Endosc, 2021. **35**(6): p. 2679-2689.

15.	Ishii, T., et al., *Prophylactic endoscopic gallbladder stent placement for cholecystitis after covered metal stent placement for distal biliary obstruction (with video).* Gastrointest Endosc, 2023. **98**(1): p. 36-42 e1.

16.	Gosain, S., et al., *Gallbladder stent placement for prevention of cholecystitis in patients receiving covered metal stent for malignant obstructive jaundice: a feasibility study.* Dig Dis Sci, 2010. **55**(8): p. 2406-11.

17.	Watanabe, M., et al., *Cholecystitis after Placement of Covered Self-Expandable Metallic Stents in Patients with Distal Malignant Biliary Obstructions.* Clin Endosc, 2021. **54**(4): p. 589-595.

18.	Yokoe, M., et al., *Tokyo Guidelines 2018: diagnostic criteria and severity grading of acute cholecystitis (with videos).* J Hepatobiliary Pancreat Sci, 2018. **25**(1): p. 41-54.

19.	Committee, A.S.o.P., et al., *Guidelines for privileging, credentialing, and proctoring to perform GI endoscopy.* Gastrointest Endosc, 2017. **85**(2): p. 273-281.

20.	Cotton, P.B., et al., *A lexicon for endoscopic adverse events: report of an ASGE workshop.* Gastrointest Endosc, 2010. **71**(3): p. 446-54.
