# Supplementary material for: Prophylactic endoscopic transpapillary gallbladder stenting at fully covered self-expandable metal stent placement to prevent cholecystitis in malignant distal biliary obstruction: A multicenter prospective single-arm study protocol (PROTECT study)
Source: PLoS One. 2026 Oct 29;21(10):e0361468. doi: 10.1371/journal.pone.0361468 (PMC13718957; doi:10.1371/journal.pone.0361468)
Supplement: Supplementary file 3 [file pone.0361468.s003.docx]

Study Protocol (English Translation)

Study title:

A multicenter, single-arm clinical study of the efficacy and safety of endoscopic gallbladder stenting (EGBS) for preventing cholecystitis after fully covered self-expandable metallic stent (FCSEMS) placement for malignant distal biliary stricture

Study representative:

Takayuki Oda, Department of Gastroenterology and Hepatology, Yokohama City University Hospital

Version: 1.3

Date of preparation: July 28, 2026

Revision History

| Version | Date prepared/revised |
| --- | --- |
| Version 1.0 | August 18, 2025 |
| Version 1.1 | February 4, 2026 |
| Version 1.2 | April 6, 2026 |
| Version 1.3 | July 28, 2026 |

**Confidentiality**

This study protocol contains confidential information owned by Yokohama City University (hereinafter, the "University").

Accordingly, no part or all of this study protocol may be disclosed, without the prior consent of Yokohama City University, to any third party other than the institutions and investigators conducting the clinical study in accordance with this protocol, the ethics review committee(s) performing review-related activities, and other persons involved with this protocol. Use of this protocol for any purpose other than the foregoing is also strictly prohibited.

**Purposes of use of personal information in clinical research at the University**

The University is an "academic research institution, etc." as specified in Article 16, paragraph 8 of the Act on the Protection of Personal Information (Act No. 57 of 2003; hereinafter, the "APPI") and is a university or other institution whose purpose is academic research.

In clinical research conducted at the University, personal information and personal data are handled for academic research purposes pursuant to Article 18, paragraph 3, items 5 and 6 of the APPI.

Contents

[**0. Overview**	7](#_Toc82429667)

[**0.1.** **Schema**	7](#_Toc82429668)

[**0.2. Study Objective and Primary Endpoint**	8](#_Toc82429669)

[**0.3. Study Population**	8](#_Toc82429670)

[**0.4. Treatment**	8](#_Toc82429671)

[**0.5. Target Sample Size**	8](#_Toc82429672)

[**0.6. Study Period**	8](#_Toc82429673)

[**0.7. Study Representative and Contact Information**	8](#_Toc82429674)

[**1. Study Objective**	9](#_Toc82429675)

[**2. Background and Scientific Rationale for the Study Plan**	9](#_Toc82429676)

[**2.1. Target Disease**	9](#_Toc82429677)

[**2.2. Standard Treatment**	9](#_Toc82429678)

[**2.3. Study Treatment**	10](#_Toc82429679)

[**2.4. Study Design and Primary Endpoint**	11](#_Toc82429680)

[**2.5 Significance of the Study**	12](#_Toc82429681)

[**3. Device Information**	12](#_Toc82429682)

[**3.1. Devices Used as FCSEMSs**	12](#_Toc82429683)

[**3.2. Devices Used for EGBS**	13](#_Toc82429684)

[**4. Criteria and Definitions Used in This Study**	14](#_Toc82429685)

[**5. Selection of Study Participants**	14](#_Toc82429686)

[**5.1. Inclusion Criteria**	14](#_Toc82429687)

[**5.2. Exclusion Criteria**	14](#_Toc82429688)

[**6. Study Plan**	15](#_Toc82429689)

[**6.1. Study Design**	15](#_Toc82429690)

[**6.2. Target Sample Size**	15](#_Toc82429691)

[**6.3. Study Period**	15](#_Toc82429692)

[**6.4. Allocation**	15](#_Toc82429693)

[**6.5. Treatment Plan**	16](#_Toc82429694)

[**7. Observations, Tests, Investigations, and Assessments**	18](#_Toc82429695)

[**7.1. Schedule**	18](#_Toc82429696)

[**7.2. Assessment Time Points and Assessment Items**	19](#_Toc82429697)

[**7.3. Assessment Methods**	20](#_Toc82429698)

[**8. Endpoints**	21](#_Toc82429699)

[**8.1. Primary Endpoint**	21](#_Toc82429700)

[**8.2. Secondary Endpoints**	21](#_Toc82429701)

[**9. Statistical Analysis**	22](#_Toc82429702)

[**9.1. Analysis Populations**	22](#_Toc82429703)

[**9.2. Rationale for the Target Sample Size**	23](#_Toc82429704)

[**9.3. Statistical Methods**	23](#_Toc82429705)

[**9.4. Interim Analysis**	24](#_Toc82429706)

[**10. Handling of Adverse Events**	24](#_Toc82429707)

[**10.1. Definition of an Adverse Event**	24](#_Toc82429708)

[**10.2. Assessment of Adverse Events**	25](#_Toc82429709)

[**10.3. Expected Adverse Events**	26](#_Toc82429710)

[**10.4. Measures to Be Taken if an Adverse Event Occurs**	26](#_Toc82429711)

[**11. Efficacy and Safety Evaluation Committee**	27](#_Toc82429712)

[**12. Anticipated Benefits and Disadvantages Associated with Study Participation**	27](#_Toc82429713)

[**12.1. Anticipated Benefits**	27](#_Toc82429714)

[**12.2. Burden and Anticipated Risks**	27](#_Toc82429715)

[**13. Procedures for Obtaining Informed Consent**	28](#_Toc82429716)

[**13.1. Handling of Consultations from Participants and Related Persons**	28](#_Toc82429717)

[**13.2. Obtaining Informed Consent from a Legally Authorized Representative**	28](#_Toc82429718)

[**13.3. Obtaining Informed Assent**	29](#_Toc82429719)

[**13.4. Research Conducted under Part 8, Section 8 of the Ethical Guidelines**	29](#_Toc82429720)

[**14. Site Registration and Participant Registration**	29](#_Toc82429721)

[**14.1. Registration Center**	29](#_Toc82429722)

[**14.2. Site Registration**	29](#_Toc82429723)

[**14.3. Participant Registration**	29](#_Toc82429724)

[**15. Transfer of Samples and Information**	30](#_Toc82429725)

[**16. Storage and Disposal of Samples and Information**	30](#_Toc82429726)

[**16.1. Secondary Use of Samples and Information**	32](#_Toc82429727)

[**17. Handling of Personal Information**	32](#_Toc82429728)

[**18. Handling of Study Results Relating to Participants (Including Incidental Findings)**	32](#_Toc82429729)

[**18.1.Genetic Counseling System**	33](#_Toc82429730)

[**19. Protocol Compliance, Deviations, and Amendments**	33](#_Toc82429731)

[**19.1. Protocol Compliance**	33](#_Toc82429732)

[**19.2. Protocol Deviations**	33](#_Toc82429733)

[**19.3. Procedures for Protocol Amendments**	33](#_Toc82429734)

[**20. Procedures for Conducting the Study**	34](#_Toc82429735)

[**20.1. Applicable Rules and Guidelines**	34](#_Toc82429736)

[**20.2. Submission to the Ethics Review Committee**	34](#_Toc82429737)

[**20.3. Permission from the Head of Each Research Institution**	34](#_Toc82429738)

[**20.4. Periodic Reports**	34](#_Toc82429739)

[**20.5. Suspension or Termination of the Study**	35](#_Toc82429740)

[**20.6. Completion of the Study**	35](#_Toc82429741)

[**21. Funding Sources and Financial Relationships, Institutional Conflicts of Interest and Individual Financial Interests, and Investigators' Conflicts of Interest**	35](#_Toc82429742)

[**21.1. Funding Sources and Financial Relationships**	35](#_Toc82429743)

[**21.2. Conflicts of Interest**	35](#_Toc82429744)

[**22. Costs and Remuneration for Study Participants**	36](#_Toc82429745)

[**23. Compensation for Health Injury**	36](#_Toc82429746)

[**24. Monitoring and Auditing**	36](#_Toc82429747)

[**24.1. Monitoring**	36](#_Toc82429748)

[**24.2. Auditing**	37](#_Toc82429749)

[**25. Public Disclosure of Study Information and Publication of Results**	37](#_Toc82429750)

[**25.1. Study Registration**	37](#_Toc82429751)

[**25.2. Publication of Study Results**	37](#_Toc82429752)

[**26. Ownership of Study Results (Intellectual Property Rights)**	38](#_Toc82429753)

[**27. Study Organization**	38](#_Toc82429754)

[**27.1. Study Representative**	38](#_Toc82429755)

[**27.2. Study Office**	38](#_Toc82429756)

[**27.3. Collaborating Research Institutions and Principal Investigators**	38](#_Toc82429757)

[**27.4. Cooperating Institutions and Responsible Persons**	39](#_Toc82429758)

[**27.5. Lead Statistician**	39](#_Toc82429759)

[**27.6. Data Center/Data Management Lead**	39](#_Toc82429760)

[**27.7. Monitoring Lead**	39](#_Toc82429761)

[**27.8. Audit Lead**	39](#_Toc82429762)

[**27.9. External Contractors**	39](#_Toc82429763)

[**28. References**	39](#_Toc82429764)

**Abbreviations and Definitions of Terms**

The abbreviations and terms used in this study protocol are shown below.

| Abbreviation | Full term | Description |
| --- | --- | --- |
| CRP | C-reactive protein | C-reactive protein |
| EGBS | endoscopic gallbladder stenting | Endoscopic gallbladder stenting |
| ERCP | endoscopic retrograde cholangiopancreatography | Endoscopic retrograde cholangiopancreatography |
| ESGE | European Society for Gastrointestinal Endoscopy | European Society for Gastrointestinal Endoscopy |
| EUS-BD | endoscopic ultrasonography-guided biliary drainage | Endoscopic ultrasonography-guided biliary drainage |
| EUS-GBD | endoscopic ultrasonography-guided gallbladder drainage | Endoscopic ultrasonography-guided gallbladder drainage |
| FCSEMS | fully covered self-expandable metallic stent | Fully covered self-expandable metallic stent |
| PTBD | percutaneous transhepatic biliary drainage | Percutaneous transhepatic biliary drainage |
| PTGBA | percutaneous transhepatic gallbladder aspiration | Percutaneous transhepatic gallbladder aspiration |
| PTGBD | percutaneous transhepatic gallbladder drainage | Percutaneous transhepatic gallbladder drainage |
| RCT | randomized controlled trial | Randomized controlled trial |
| UCSEMS | uncovered self-expandable metallic stent | Uncovered self-expandable metallic stent |

# 0. **Overview**

## **0.1. Schema**

## **0.2. Study Objective and Primary Endpoint**

Objective: To investigate whether concomitant endoscopic gallbladder stenting (EGBS) can reduce the risk of acute cholecystitis after fully covered self-expandable metallic stent (FCSEMS) placement for malignant distal biliary stricture.

Primary endpoint: Proportion of patients who develop acute cholecystitis within 30 days after FCSEMS placement among all patients in whom EGBS is attempted

## **0.3. Study Population**

Patients aged 18 years or older with obstructive jaundice due to malignant distal biliary stricture

## 0.4. **Treatment**

EGBS will be attempted in eligible patients when an FCSEMS is placed for malignant distal biliary stricture.

## **0.5. Target Sample Size**

50 patients

## **0.6. Study Period**

Study period: From the date on which the head of the research institution grants permission to conduct the study through March 31, 2030 (5 years)

Enrollment period: From the date on which the head of the research institution grants permission to conduct the study through March 31, 2028

Follow-up period: Through April 30, 2028

## **0.7. Study Representative and Contact Information**

Affiliation: Department of Gastroenterology and Hepatology, Yokohama City University Hospital

Name: Takayuki Oda

Contact: t246016b@yokohama-cu.ac.jp

# **1. Study Objective**

To conduct a single-arm exploratory evaluation of the efficacy of EGBS at the time of FCSEMS placement in patients with malignant distal biliary stricture, as assessed by the proportion who develop acute cholecystitis after the procedure.

# **2. Background and Scientific Rationale for the Study Plan**

## **2.1. Target Disease**

Biliary obstruction caused by progression of a malignant disease, such as pancreatic or biliary tract cancer, can result in obstructive jaundice. Japanese statistics show that 44,448 patients were diagnosed with pancreatic cancer and 21,392 with biliary tract cancer in 2020, and the incidence of both diseases is increasing [1]. As the number of affected patients increases, the number of patients with obstructive jaundice due to malignant distal biliary stricture is also expected to rise. Progression of obstructive jaundice may lead to hepatic failure or acute cholangitis, and a fully covered self-expandable metallic stent (FCSEMS) may be placed to relieve the obstruction. Acute cholecystitis can develop after FCSEMS placement, with a reported incidence of 15% [2]. The onset of acute cholecystitis may delay subsequent surgery or initiation of chemotherapy and can have a major effect on prognosis. This study was therefore planned because EGBS at the time of FCSEMS placement may reduce the risk of postprocedural cholecystitis.

## **2.2. Standard Treatment**

**2.2.1. Current Standard Treatment**

Biliary drainage by endoscopic retrograde cholangiopancreatography (ERCP) is the standard treatment for malignant distal biliary stricture [3]. The usefulness of other biliary drainage methods, including percutaneous transhepatic biliary drainage (PTBD) and endoscopic ultrasonography-guided biliary drainage (EUS-BD), has also been reported. However, a retrospective study of patients who underwent ERCP or PTBD for malignant biliary stricture reported a higher adverse-event rate with PTBD than with ERCP (12.3% vs 8.6%) [4]. Another report also found higher mortality with PTBD than with ERCP (6.86% vs 4.35%) [5]. Randomized controlled trials comparing ERCP and EUS-BD [6-8] found comparable technical and clinical success rates and no difference in adverse-event rates. Nevertheless, EUS-BD is technically more difficult than ERCP, and adverse events caused by technical failure can sometimes be fatal; therefore, it is positioned as an alternative for patients in whom ERCP is difficult. For these reasons, ERCP-guided biliary drainage is the first-line treatment.

**2.2.2. Efficacy of the Current Standard Treatment**

Plastic stents and metal stents are used for biliary drainage during ERCP. In patients with malignant distal biliary stricture, the larger diameter of metal stents provides longer patency, and metal stents are therefore widely used [9, 10]. Davids et al. reported that median stent patency was significantly longer with metal stents than with plastic stents (273 days vs 126 days) [10]. Guidelines from the European Society for Gastrointestinal Endoscopy (ESGE) also recommend metal stent placement, including from a cost-effectiveness perspective, for patients with distal malignant biliary obstruction whose expected survival is at least 4 months [11]. Metal stents include FCSEMSs, in which the metal mesh is covered with silicone, and uncovered self-expandable metallic stents (UCSEMSs). When a UCSEMS is used for malignant distal biliary stricture, stent occlusion due to so-called tumor ingrowth, in which the tumor grows through the interstices of the metal mesh into the bile duct, is a concern. Because FCSEMSs can prevent tumor ingrowth, their median patency has been reported to be longer than that of UCSEMSs (304 days vs 166 days). The ability to remove an FCSEMS when necessary also supports its advantages [12].

**2.2.3. Safety of the Current Standard Treatment**

Stent dysfunction associated with long-term placement has been reported in 20% of patients after FCSEMS placement [11]. Other reported complications include acute pancreatitis (incidence, approximately 8%) [13] and acute cholecystitis. In a retrospective study limited to patients who received an FCSEMS, Ishii et al. reported a 15% incidence of acute cholecystitis after FCSEMS placement [2].

**2.2.4. Limitations of the Current Standard Treatment**

As described in Section 2.2.3, acute cholecystitis is one complication of FCSEMS placement. Obstruction of the cystic duct orifice by the metal stent and reflux of contrast medium into the gallbladder during cholangiography have been reported to be associated with the development of acute cholecystitis [14]. When acute cholecystitis develops, invasive procedures such as percutaneous transhepatic gallbladder aspiration (PTGBA), percutaneous transhepatic gallbladder drainage (PTGBD), endoscopic ultrasonography-guided gallbladder drainage (EUS-GBD), or cholecystectomy may be required. Acute cholecystitis may delay subsequent surgery or initiation of chemotherapy and can be fatal [15].

## **2.3. Study Treatment**

**2.3.1. Overview of the Study Treatment**

As described in Section 2.2.4, obstruction of the cystic duct orifice by the stent is considered a mechanism of acute cholecystitis after FCSEMS placement [14]. Performing endoscopic gallbladder stenting (EGBS) at the time of FCSEMS placement is expected to allow bile in the gallbladder to drain into the duodenum after FCSEMS placement and thereby reduce the risk of acute cholecystitis.

**2.3.2. Efficacy of the Study Treatment**

Two reports have evaluated the efficacy of concomitant EGBS when an FCSEMS is placed in patients with malignant distal biliary stricture. Gosain et al. attempted EGBS at the time of FCSEMS placement in 19 patients with malignant distal biliary stricture; EGBS was successful in 11 patients (58%), and no postprocedural complications, including cholecystitis, occurred [16]. Ishii et al. retrospectively compared patients who did and did not undergo concomitant EGBS. The incidence of cholecystitis was 4% in the EGBS group and 21% in the non-EGBS group, indicating that EGBS at the time of FCSEMS placement for malignant distal biliary stricture significantly reduced the incidence of cholecystitis [15]. In the report by Ishii et al., the EGBS success rate was 75%, and the incidence of cholecystitis among patients in whom EGBS was attempted was 3.1% [15].

**2.3.3. Safety of the Study Treatment**

The same reports described in Section 2.3.2 provide safety information. Gosain et al. reported that guidewire perforation of the cystic duct occurred in 3 of 8 patients (37.5%) in whom EGBS was unsuccessful, and one of these patients required emergency cholecystectomy [16]. Ishii et al. reported acute cholecystitis in 1 of 32 patients (3.1%), post-ERCP pancreatitis in 2 patients (6.3%), and guidewire perforation of the cystic duct in 1 patient (3.1%) among patients in whom EGBS was attempted [15].

**2.3.4. Risks and Benefits of the Study Treatment**

The risks that the study treatment may pose to participants are described first. Few studies have evaluated the efficacy of EGBS at the time of FCSEMS placement for malignant distal biliary stricture [15, 16], and no prospective comparative study has been conducted. The success rate of EGBS is reported to be approximately 70% [15]. Because EGBS requires cholangiography to delineate the course of the cystic duct, a greater volume of contrast medium may be used than when EGBS is not performed. Reflux of contrast medium into the gallbladder has been reported to increase the risk of postprocedural cholecystitis [17]; consequently, an unsuccessful EGBS attempt could increase the risk of acute cholecystitis after the procedure. Attempting EGBS also carries a risk of guidewire perforation of the cystic duct. Although previous reports have not described cases in which an EGBS attempt prevented FCSEMS placement, FCSEMS placement could become difficult in some circumstances. Attempting EGBS is also expected to prolong the overall ERCP procedure time. Conversely, as described above, the potential benefit of the study treatment is a reduction in the incidence of cholecystitis after FCSEMS placement. Preventing cholecystitis may permit earlier initiation of chemotherapy and could improve patient prognosis. On the basis of the efficacy and safety data for EGBS described in Sections 2.3.2 and 2.3.3, the potential benefits of EGBS at the time of FCSEMS placement are considered likely to outweigh its risks.

## **2.4. Study Design and Primary Endpoint**

**2.4.1. Study Design**

This is a prospective, single-arm exploratory study. As described in Section 2.3.2, few reports have evaluated the efficacy of EGBS at the time of FCSEMS placement for malignant distal biliary stricture. Therefore, efficacy will first be evaluated on an exploratory basis. Based on the number of FCSEMS placements for malignant distal biliary stricture at the four participating institutions in recent years, approximately 27 patients are expected to be enrolled per year; a single-arm exploratory evaluation is therefore planned first on the basis of feasibility. If this study suggests potential efficacy of EGBS, a future confirmatory study is planned.

**2.4.1. Primary Endpoint**

The primary endpoint is the "proportion of patients who develop acute cholecystitis within 30 days after FCSEMS placement among all patients in whom EGBS is attempted." This study aims to explore whether EGBS performed at the time of FCSEMS placement in patients with malignant distal biliary stricture can reduce the postprocedural incidence of cholecystitis. The incidence of cholecystitis after FCSEMS placement was therefore selected as the primary outcome measure. The 30-day time window was selected on the basis of a previous report indicating that approximately 80% of cases of cholecystitis after FCSEMS placement occur within 30 days [2].

## **2.5. Significance of the Study**

If this study demonstrates the potential efficacy of EGBS, concomitant EGBS at the time of FCSEMS placement could become a standard treatment, reduce the incidence of cholecystitis after FCSEMS placement, and potentially improve the prognosis of patients with cancer; thus, the study may have substantial clinical value. As described in Section 2.3.2, few relevant studies have been reported, and the efficacy of EGBS has not been clearly established. The reported success rate of EGBS is approximately 70% [15], and unsuccessful procedures could instead increase the incidence of cholecystitis by prolonging procedure time or causing reflux of contrast medium into the gallbladder. If attempting EGBS does not show the potential to reduce the incidence of cholecystitis after FCSEMS placement, this finding could conversely suggest that EGBS is unnecessary at the time of FCSEMS placement and would also provide important evidence.

# **3. Device Information**

## **3.1. Devices Used as FCSEMSs**

The following two devices will be used as FCSEMSs in this study. Both devices will have a diameter of 10 mm. The responsible investigator will select an appropriate stent length for each patient.

1) Product name: HANAROSTENT Biliary Full Cover NEO

Marketing authorization holder: Boston Scientific Japan K.K.

Intended use or effect:

This stent is intended for endoscopic, percutaneous, or open surgical insertion into the biliary system (gallbladder, cystic duct, and bile ducts) to provide drainage of pus or fluid, irrigation, dilation or support of a stenotic site, prevention of stenosis, or related treatment. It is delivered to the obstructed site using a delivery system. This is a sterile, disposable, single-use product; the stent cannot be reloaded or reused.

Appearance:

Source: https://www.bostonscientific.com/jp-JP/products/stent/HANAROSTENT_Biliary_Full_Cover_NEO.html

2) Product name: BONASTENT Biliary Fully Covered Stent

Marketing authorization holder: Medicos Hirata Inc.

Intended use or effect:

This device consists of a stent that is inserted endoscopically into a bile duct stenosed by a malignant tumor to maintain dilation of the stenotic segment and provide drainage, together with a delivery system for inserting the stent.

Appearance:

Source: http://www.medicoshirata-products.com/article.php?p_id=36&b_id=5

## **3.2. Devices Used for EGBS**

The following two devices will be used for EGBS in this study. The Through & Pass Double Pigtail stent has a diameter of 7 Fr, whereas the IYO stent has a diameter of 5 Fr; their efficacy in preventing cholecystitis after FCSEMS placement may therefore differ. Accordingly, as described in Section 8.2, subgroup analyses by stent type will be performed as secondary endpoint analyses.

1. Product name: Through & Pass Double Pigtail

Marketing authorization holder: Gadelius Medical K.K.

Intended use or effect:

This tube stent and stent system is intended for long-term endoscopic placement in the biliary system (gallbladder, cystic duct, and bile ducts) or pancreatic duct for drainage of pus or fluid, dilation or support of a stenotic site, or prevention of stenosis.

Appearance:

Source: https://www.gadeliusmedical.com/ja/products/Catheters/drainage.html

1. Product name: IYO Stent for the Gallbladder

Marketing authorization holder: Gadelius Medical K.K.

Intended use or effect:

This tube stent and stent system is intended for long-term endoscopic placement in the biliary system (gallbladder, cystic duct, and bile ducts) or pancreatic duct for drainage of pus or fluid, dilation or support of a stenotic site, or prevention of stenosis.

Appearance:

Source: https://www.gadeliusmedical.com/ja/products/Catheters/ps.html

# **4. Criteria and Definitions Used in This Study**

The distal bile duct is defined in accordance with the eighth edition of the General Rules for Clinical and Pathological Studies on Cancer of the Biliary Tract.

The diagnostic criteria and severity grading for acute cholecystitis will be based on the criteria in the Tokyo Guidelines 2018 for the management of acute cholangitis and cholecystitis [18]. Only cases meeting the criteria for a definite diagnosis will be diagnosed as cholecystitis; cases meeting only the criteria for a suspected diagnosis will not be treated as cholecystitis. This is because abdominal pain caused by the tumor together with elevations in the white blood cell count and C-reactive protein level may meet the criteria for suspected acute cholecystitis even when cholecystitis has not developed.

# **5. Selection of Study Participants**

## **5.1. Inclusion Criteria**

(1) Patients aged 18 years or older at enrollment

(2) Patients diagnosed with obstructive jaundice caused by malignant distal biliary stricture due to pancreatic cancer, distal bile duct cancer, ampullary cancer, lymph-node metastasis, or another malignant tumor, irrespective of cancer type

(3) Patients with no history of cholecystectomy

(4) Patients with an expected survival of at least 4 months

(5) Patients from whom written consent to participate in this study can be obtained, either from the patient or from a legally authorized representative*

*The circumstances in which consent from a legally authorized representative is permitted are described in detail in Section 13.2, "Obtaining Informed Consent from a Legally Authorized Representative."

## **5.2. Exclusion Criteria**

(1) Patients in whom gallbladder drainage by PTGBD, EUS-GBD, or gallbladder stent placement by EGBS has already been performed at enrollment
(2) Patients with surgically altered anatomy other than Billroth I reconstruction that makes ERCP with a side-viewing endoscope difficult
(3) Patients will be excluded only when both of the following conditions are met on pre-ERCP CT and/or MRI: (i) the cystic duct orifice is clearly identifiable, and (ii) the proximal end of the planned FCSEMS is expected to remain distal to the cystic duct orifice without overlapping it. If either condition cannot be determined, the patient will not be excluded on the basis of this criterion. No fixed cutoff will be applied for the distance between the proximal margin of the malignant biliary stricture and the cystic duct orifice. The imaging assessment will be performed independently by two experienced endoscopists at each participating institution, and exclusion will require agreement between both endoscopists.
(4) Patients in whom ERCP is difficult because of gastrointestinal stenosis caused by tumor invasion of the duodenum
(5) Patients with upper gastrointestinal perforation for whom ERCP is contraindicated
(6) Patients who are pregnant, may be pregnant, or wish to become pregnant during the treatment period
(7) Patients for whom in-person follow-up by the treating physician 30 days after FCSEMS placement is expected to be difficult

(8) Patients participating in a clinical trial of a drug, medical device, or other intervention concurrently with this study or within 30 days before enrollment in this study

(9) Other patients considered ineligible by the investigator

# **6. Study Plan**

## **6.1. Study Design**

This is a prospective, single-arm exploratory study.

## **6.2. Target Sample Size**

Target sample size for the entire study: 50 patients

See Section 9.2, "Rationale for the Target Sample Size."

## **6.3. Study Period**

Study period: From the date on which the head of the research institution grants permission to conduct the study through March 31, 2030 (5 years)

Enrollment period: From the date on which the head of the research institution grants permission to conduct the study through March 31, 2028

Follow-up period: Through April 30, 2028

## **6.4. Allocation**

**6.4.1. Allocation Method and Allocation Adjustment Factors**

Not applicable because no allocation will be performed in this study.

**6.4.2. Blinding Method**

Not applicable because this study will not be blinded.

**6.4.3. Criteria and Procedures for Unblinding (Code Breaking)**

Not applicable because this study will not be blinded.

## **6.5. Treatment Plan**

**6.5.1. Protocol Treatment**

**6.5.1.1. FCSEMS plus EGBS Group**

Within 30 days after enrollment, when an FCSEMS is placed under ERCP guidance for malignant distal biliary stricture, EGBS will be attempted to prevent postprocedural cholecystitis.

First, the endoscope will be advanced into the duodenum, and a guidewire will be inserted into the bile duct through the major duodenal papilla. A catheter will then be advanced over the guidewire, and cholangiography will be performed to confirm the malignant distal biliary stricture and attempt to identify the cystic duct orifice. Endoscopic sphincterotomy will be added if necessary.

After cholangiography, regardless of whether the cystic duct orifice is visualized, the endoscopist will intentionally manipulate a guidewire toward the visualized or presumed location of the cystic duct orifice with the aim of advancing it through the cystic duct into the gallbladder. Initiation of this intentional guidewire manipulation is defined as an "EGBS attempt." Visualization of the cystic duct orifice alone, without guidewire manipulation toward the visualized or presumed location, will not be considered an EGBS attempt.

If the guidewire is successfully advanced into the gallbladder, a gallbladder stent will be placed to establish communication between the gallbladder and the duodenum. One of the two prespecified gallbladder stents will be used: a 7-Fr Through & Pass Double Pigtail stent or a 5-Fr IYO stent. The operator will select the stent type and caliber on the basis of the course and diameter of the cystic duct observed on cholangiography, and the type and caliber of the stent actually placed will be recorded for each patient.

If EGBS has not been successfully completed within 60 minutes after endoscope insertion, the EGBS attempt will be discontinued. However, the attempt will be discontinued before 60 minutes if the operator judges that continuing the procedure is technically difficult or unsafe. The 60-minute period is elapsed time from endoscope insertion and includes routine ERCP steps performed before the EGBS attempt, such as biliary cannulation, cholangiography, and endoscopic sphincterotomy when required. It does not mean that EGBS-related guidewire manipulation alone will be continued for 60 minutes.

Total procedure time is defined as the time from endoscope insertion to withdrawal. EGBS attempt time is defined as the time from initiation of intentional guidewire manipulation toward the visualized or presumed location of the cystic duct orifice to either successful placement of the gallbladder stent or discontinuation of the EGBS attempt. FCSEMS placement time is defined as the time from initiation of advancement of the FCSEMS delivery system over the guidewire placed in the bile duct to completion of FCSEMS deployment.

Regardless of whether EGBS is successful, a HANAROSTENT Biliary Full Cover NEO or a fully covered BONASTENT will be placed across the distal biliary stricture to complete the procedure. The FCSEMS type used will be recorded for each patient. For all guidewires and catheters used in the EGBS attempt, the marketing authorization holder and product name will also be recorded for each patient.

The entire procedure will be performed during hospitalization. With reference to a previous report [19], both the operator and assistant will be limited to endoscopists who have each performed at least 200 ERCP procedures as the primary operator. If no postprocedural complication occurs, the anticipated hospital stay is approximately 1 week.

If acute cholecystitis develops during follow-up, treatment will be provided at each institution in accordance with the Tokyo Guidelines 2018 for the management of acute cholangitis and cholecystitis [18], and the treatment provided will be recorded.

**6.5.1.2. Control Group**

This is a single-arm exploratory study; therefore, there is no control group.

**6.5.2. Criteria for Drug Administration**

**6.5.2.1. Criteria for Initiation of Drug Administration**

Not applicable because no drug will be administered for research purposes in this study.

**6.5.2.2. Criteria for Dose Reduction or Treatment Interruption**

Not applicable because no drug will be administered for research purposes in this study.

**6.5.2.3. Criteria for Resumption**

Not applicable because no drug will be administered for research purposes in this study.

**6.5.3. Concomitant Medications and Therapies**

**6.5.3.1. Prohibited Concomitant Therapies**

None

**6.5.3.2. Concomitant Therapies Requiring Caution**

None

**6.5.3.3. Permitted Concomitant Therapies**

None

**6.5.4. Discontinuation of the Study for an Individual Participant**

**6.5.4.1. Discontinuation Criteria**

Participation in the study will be discontinued if any of the following criteria are met:

(1) The patient or legally authorized representative requests withdrawal of consent

(2) After enrollment, the patient is found not to meet an inclusion criterion or to meet an exclusion criterion and is therefore found to be ineligible

(3) After enrollment, worsening symptoms or findings of the underlying disease make FCSEMS placement difficult

(4) After enrollment but before ERCP for FCSEMS placement, the patient develops acute cholecystitis and undergoes PTGBD, EUS-GBD, or cholecystectomy

(5) A major deviation from the study protocol occurs

(6) Death

(7) Continued follow-up becomes difficult because of transfer to another hospital or another reason

(8) Pregnancy is confirmed

(9) The principal investigator or subinvestigator otherwise judges that continuing the study is inadvisable

**6.5.4.2. Discontinuation Procedures**

(1) If the principal investigator or subinvestigator decides during the study period, before the procedure, not to perform EGBS when the participant undergoes FCSEMS placement, the investigator will promptly explain this decision to the participant and perform the necessary observations, tests, and assessments.

(2) The date on which EGBS at the time of FCSEMS placement by ERCP was prospectively discontinued and the reason for discontinuation will be recorded in the case report form.

**6.5.4.3. Treatment after Completion (Discontinuation) of Protocol Treatment**

Not applicable in this study.

# **7. Observations, Tests, Investigations, and Assessments**

## **7.1. Schedule**

| Time point  Item | | Screening  period | ERCP |  | | Follow-up  period | At discontinuation |
| --- | --- | --- | --- | --- | --- | --- | --- |
|  |  |  | Day 0 | Day 1 | Day 30 | Through 2028/04/30*3 |  |
| Permitted window (days) | | −30 to −1 | 0 | 0 | +10 | ±14 | ±7 |
| Informed consent | | ○ |  |  |  |  |  |
| Participant characteristics | | ○ |  |  |  |  |  |
| Protocol treatment | |  | ○ |  |  |  |  |
| Symptoms/findings | | ○ | ○ | ○ | ○^*2^ |  |  |
| Vital signs | | ○ | ○ | ○ |  |  |  |
| Clinical  laboratory  tests | Hematologic tests | ○ |  | ○ | ○ |  |  |
|  | Blood chemistry tests | ○ |  | ○ | ○ |  |  |
| Imaging (CT)*1 | | ○ |  |  |  |  |  |
| Adverse-event monitoring | |  |  |  |  |  |  |

*1 If no CT scan was obtained within 30 days before study enrollment, a CT scan obtained within 60 days before enrollment may be used instead.

*2 The presence or absence of cholecystitis after FCSEMS placement should preferably be confirmed in person with the participant; if an in-person assessment is difficult, confirmation by telephone is permitted.

*3 On April 30, 2028 (±14 days), the presence or absence of cholecystitis after FCSEMS placement will be confirmed by telephone or clinical examination. If confirmation during this period is difficult, follow-up will extend through the date of the most recent earlier examination.

## **7.2. Assessment Time Points and Assessment Items**

7.2.1. Screening Period

(1) Participant characteristics

• Sex, age, height, weight, medical history, and comorbidities

• Date informed consent was obtained

• Date of diagnosis of malignant distal biliary stricture, underlying disease, presence or absence of gallstones, and presence or absence of tumor involvement of the cystic duct orifice

(2) Subjective symptoms and objective findings

A physical examination will be performed with particular attention to the major symptoms of acute cholecystitis.

(3) Vital signs

• Blood pressure, pulse rate, and body temperature

(4) Hematologic tests

• White blood cell count, red blood cell count, hematocrit, hemoglobin, and platelet count

(5) Blood chemistry tests

• Serum albumin, total bilirubin, direct bilirubin, AST, ALT, LDH, CRP, ALP, γ-GTP, AMY, BUN, serum creatinine, Na, K, and Cl

(6) CT

7.2.2. Day 0

(1) Subjective symptoms and objective findings

A physical examination will be performed with particular attention to the major symptoms of acute cholecystitis.

(2) Vital signs

• Blood pressure, pulse rate, and body temperature

(3) Adverse-event assessment

If an adverse event is suspected on the basis of subjective symptoms, objective findings, or other information, blood tests and imaging studies will be considered.

7.2.3. Day 1

(1) Subjective symptoms and objective findings

A physical examination will be performed with particular attention to the major symptoms of acute cholecystitis.

(2) Vital signs

• Blood pressure, pulse rate, and body temperature

(3) Hematologic tests

• White blood cell count, red blood cell count, hematocrit, hemoglobin, and platelet count

(4) Blood chemistry tests

• Serum albumin, total bilirubin, direct bilirubin, AST, ALT, LDH, CRP, ALP, γ-GTP, AMY, BUN, serum creatinine, Na, K, and Cl

(5) Adverse-event assessment

If an adverse event is suspected on the basis of subjective symptoms, objective findings, or other information, blood tests and imaging studies will be considered.

7.2.4. Day 30

(1) Subjective symptoms and objective findings

A physical examination will be performed with particular attention to the major symptoms of acute cholecystitis.

(2) Hematologic tests

• White blood cell count, red blood cell count, hematocrit, hemoglobin, and platelet count

(3) Blood chemistry tests

• Serum albumin, total bilirubin, direct bilirubin, AST, ALT, LDH, CRP, ALP, γ-GTP, AMY, BUN, serum creatinine, Na, K, and Cl

(4) Adverse-event assessment

If an adverse event is suspected on the basis of subjective symptoms, objective findings, or other information, blood tests and imaging studies will be considered.

7.2.5. Follow-up Period (through April 30, 2028)

(1) Confirmation of whether cholecystitis has developed

(2) Adverse-event assessment

If an adverse event is suspected on the basis of subjective symptoms, objective findings, or other information, blood tests and imaging studies will be considered.

## **7.3. Assessment Methods**

CT acquisition parameters will be as follows:

• Scan range: The upper abdomen must be included to permit evaluation of the common bile duct.

• Slice thickness: 5 mm or less.

• Use of contrast medium: Contrast-enhanced CT will be used in principle; noncontrast CT is permitted in patients with an allergy to contrast medium.

# **8. Endpoints**

## **8.1. Primary Endpoint**

• Proportion of patients who develop acute cholecystitis within 30 days after FCSEMS placement among all patients in whom EGBS is attempted

[Measurement Method and Definitions of Events]

Cholecystitis will be diagnosed according to the diagnostic criteria for acute cholecystitis in the Tokyo Guidelines 2018 for the management of acute cholangitis and cholecystitis [18]. Only cases meeting the criteria for a definite diagnosis will be diagnosed as cholecystitis; cases classified as suspected will not be treated as cholecystitis. When cholecystitis is diagnosed, severity will also be assessed according to the Tokyo Guidelines 2018. The principal investigator at each institution will perform the assessment. Blood tests and a physical examination will be performed on the day after FCSEMS placement. Thereafter, if fever, abdominal pain, or another relevant symptom develops, the participant will be examined for the presence or absence of cholecystitis. If in-person follow-up by the treating physician becomes difficult within 30 days after FCSEMS placement because of an unavoidable circumstance, such as an urgent transfer to another hospital, the participant will be interviewed by telephone to confirm whether abdominal pain or fever occurred.

Development of acute cholecystitis within 30 days after FCSEMS placement will constitute a primary endpoint event. Cholecystectomy or gallbladder drainage performed to treat acute cholecystitis will also be treated as a primary endpoint event.

Death without acute cholecystitis within 30 days after FCSEMS placement, or cholecystectomy or gallbladder drainage performed for an indication other than acute cholecystitis, will not be counted as an event, and the participant will remain in the denominator. The number of applicable participants, timing of the event or intervention, and reasons will be reported separately.

[Rationale]

This study aims to explore whether EGBS performed at the time of FCSEMS placement in patients with malignant distal biliary stricture can reduce the postprocedural incidence of cholecystitis. The incidence of cholecystitis after FCSEMS placement was therefore selected as the primary outcome measure. The 30-day time window was selected on the basis of a previous report indicating that approximately 80% of cases of cholecystitis after FCSEMS placement occur within 30 days [2].

## **8.2. Secondary Endpoints**

• EGBS success rate

EGBS success is defined as successful placement of a Through & Pass Double Pigtail stent or an IYO stent in the gallbladder before FCSEMS placement. The rate will be calculated as the number of participants with successful EGBS divided by the number of participants in whom EGBS was attempted.

• Incidence of EGBS-related adverse events

Adverse events occurring in relation to the endoscopic procedures performed in this study will be assessed in accordance with Section 10.2. The incidence will be calculated as the number of participants who experience at least one endoscopic procedure-related adverse event divided by the number of participants included in the SAS. The type and severity of each adverse event and its causal relationship to protocol treatment will also be recorded.

• Incidence of cholecystitis after SEMS placement among participants with successful EGBS

The diagnosis and method of assessment of cholecystitis will be the same as for the primary endpoint. The incidence will be calculated as the number of participants who develop cholecystitis after successful EGBS and FCSEMS placement divided by the number of participants with successful EGBS and FCSEMS placement. The incidence of cholecystitis will also be assessed separately for participants who receive a Through & Pass Double Pigtail stent and those who receive an IYO Stent for the Gallbladder.

• Cumulative incidence of cholecystitis from day 31 after FCSEMS placement

The diagnosis and method of assessment of cholecystitis will be the same as for the primary endpoint. With day 31 after FCSEMS placement as the landmark, the cumulative incidence will be assessed while accounting for competing events such as death among participants who are alive and under follow-up on day 31 and who have not developed definite acute cholecystitis before that time.

# **9. Statistical Analysis**

## **9.1. Analysis Populations**

The analysis populations are defined below. The FAS will be the primary analysis population.

**9.1.1. Full Analysis Set (FAS)**

The FAS will include all participants enrolled in the study in whom an EGBS attempt was initiated and for whom at least one postprocedural outcome assessment is available, regardless of the technical success or failure of EGBS or subsequent treatment. Participants who did not proceed to an EGBS attempt after enrollment, participants for whom no postprocedural outcome information is available, participants for whom baseline data are unavailable, and participants with a major protocol violation will be excluded from the FAS. The number of excluded participants and the reasons for exclusion will be reported.

**9.1.2. Per-Protocol Set (PPS)**

The PPS will comprise the FAS after exclusion of participants with a major protocol violation related to the inclusion or exclusion criteria.

**9.1.3. Safety Analysis Set (SAS)**

The SAS will include all participants enrolled in the study in whom the protocol-specified endoscopic procedure—ERCP undertaken with the intention of both FCSEMS placement and an EGBS attempt—was initiated.

**9.1.4. Rationale for the Analysis Populations**

The FAS is defined to evaluate outcomes of the treatment strategy of attempting EGBS at the time of FCSEMS placement, regardless of the technical success or failure of EGBS. Therefore, participants with technical failure of EGBS will be included in the FAS as long as postprocedural outcome information is available.

Conversely, participants who do not proceed to an EGBS attempt and those for whom no postprocedural outcome information is available will not be included in the FAS because the study treatment strategy or postprocedural outcomes cannot be evaluated. Analyses using the PPS will be conducted as supplementary analyses. The purpose of the PPS analyses is to evaluate the effect in a population that adhered more strictly to the protocol under the conditions envisaged for this study.

## **9.2. Rationale for the Target Sample Size**

Previous studies reported an incidence of cholecystitis within 30 days after FCSEMS placement of 12.1% (41/339) when EGBS was not attempted [2] and 3.1% (1/32) when EGBS was attempted at the time of SEMS placement [15]. The primary endpoint incidence in this study is assumed to be 3%. The number of participants expected to be enrolled during the 2-year enrollment period at the four participating institutions is 54; therefore, the target enrollment is 50 patients. Allowing for 10% discontinuation or protocol deviation, the two-sided 90% Wilson score confidence interval with 45 participants was calculated to range from 0.8% to 10.5%.

## **9.3. Statistical Methods**

**9.3.1. Analysis of the Primary Endpoint**

**9.3.1.1. Primary Analysis**

In the FAS, the number and proportion of participants who develop acute cholecystitis within 30 days after FCSEMS placement will be calculated, together with a two-sided 90% confidence interval using the Wilson score method.

If a participant is lost to follow-up before day 30 and the primary endpoint cannot be ascertained, the endpoint will be treated as missing and the participant will be excluded from the denominator used to calculate the incidence. Missing values will not be imputed. The number of applicable participants, available follow-up duration, and reasons for loss to follow-up will be reported.

**9.3.1.2. Secondary Analysis**

For an exploratory comparison with a previous study, a one-sample test for a proportion will be performed for the incidence of acute cholecystitis within 30 days after FCSEMS placement, using a null proportion of 12.1% and a single two-sided significance level of 10%. This is an exploratory analysis intended to inform the planning of a future comparative study, and its result alone will not be used to conclude that prophylactic EGBS has a causal treatment effect.

**9.3.1.3. Supplementary Analyses**

A two-sided 95% confidence interval using the Wilson score method will also be calculated for the primary endpoint. In addition, the primary analysis will be repeated in the PPS.

The incidence will also be summarized exploratorily by the type of FCSEMS used; no formal statistical comparison will be performed.

**9.3.2. Analyses of Secondary Endpoints**

**9.3.2.1. Technical Success Rate of EGBS**

Among participants in the SAS in whom an EGBS attempt as defined in Section 6.5.1.1 was initiated, the number and proportion with technical success of EGBS will be calculated, together with a two-sided 90% confidence interval using the Wilson score method.

**9.3.2.2. Endoscopic Procedure-Related Adverse Events**

In the SAS, the number and proportion of participants who experience at least one endoscopic procedure-related adverse event will be calculated, together with a two-sided 90% confidence interval using the Wilson score method. The type and severity of each adverse event and its causal relationship to protocol treatment will also be summarized descriptively.

**9.3.2.3. Acute Cholecystitis within 30 Days among Participants with Technical Success of EGBS**

Among participants with technical success of EGBS, the number and proportion who develop acute cholecystitis within 30 days after FCSEMS placement will be calculated, together with a two-sided 90% confidence interval using the Wilson score method. The incidence will also be summarized exploratorily by the type and caliber of the gallbladder stent placed.

**9.3.2.4. Acute Cholecystitis from Day 31 after FCSEMS Placement**

Among participants who are alive and under follow-up on day 31 after FCSEMS placement and who have not developed definite acute cholecystitis before that time, a landmark time-to-event analysis will be conducted with day 31 as the time origin. Death before definite acute cholecystitis and cholecystectomy or gallbladder drainage performed for an indication other than definite acute cholecystitis will be treated as competing events. The cumulative incidence curve for definite acute cholecystitis will be estimated using the Aalen–Johansen method, and the median actual duration of observation from the landmark to the end of observation will be reported.

**9.3.2.5. Other Secondary Endpoints**

The severity and treatment of acute cholecystitis and items concerning the devices used will be summarized descriptively.

## **9.4. Interim Analysis**

No interim analysis will be performed in this study.

# **10. Handling of Adverse Events**

## **10.1. Definition of an Adverse Event**

An adverse event (AE) is any untoward or unintended medical occurrence in a study participant, regardless of whether it has a causal relationship with the study. This includes unintended signs, clinically significant changes in laboratory values, symptoms, and worsening of complications.

In this study, events occurring from the start of treatment through the end of the follow-up period will be handled as adverse events, and data will be collected for serious adverse events and adverse events of moderate or greater severity. Events judged by the principal investigator to be attributable to worsening of the underlying disease will be excluded.

## **10.2. Assessment of Adverse Events**

The severity of AEs related to ERCP procedures will be assessed in accordance with the definitions and grading system for major adverse events associated with ERCP proposed by Cotton et al. [20].

• Definitions of major adverse events associated with ERCP procedures

1. Perforation: Extravasation of contrast medium or air outside the gastrointestinal tract.
2. Penetration: Unintended penetration of a device beyond the gastrointestinal tract without frank perforation.
3. Device impaction: A condition in which the device cannot be removed.
4. Bleeding: Hematemesis or melena, or a decrease in hemoglobin of more than 2 g/dL.
5. Cholangitis: Fever of 38°C lasting more than 24 hours in association with bile stasis.
6. Post-ERCP pancreatitis: Epigastric pain accompanied by an elevation in amylase or lipase to at least three times the upper limit of normal.

• Grading of adverse events associated with ERCP procedures

1. Mild

An ERCP-related procedure is discontinued because of the adverse event.

A medical consultation with another department is obtained because of the adverse event.

The adverse event prolongs hospitalization by 3 days or less.

1. Moderate

The adverse event prolongs hospitalization by 4 to 10 days.

The adverse event results in admission to an intensive care unit for no more than 1 night.

The adverse event requires blood transfusion.

The adverse event requires an endoscopic procedure or endovascular treatment.

1. Severe

The adverse event prolongs hospitalization by 11 days or more.

The adverse event results in admission to an intensive care unit for 2 nights or more.

The adverse event requires surgery.

The adverse event results in permanent disability.

1. Fatal complication

The patient dies as a result of the adverse event.

The following hospitalizations will not be considered adverse events in this study for purposes of the prolongation-of-hospitalization criteria above:

1) Elective surgery planned before the informed consent form was signed

2) Hospitalization or prolongation of hospitalization for circumstances unrelated to the patient's health and not requiring medical or surgical intervention (e.g., lack of housing, financial hardship, respite care for a caregiver, or family circumstances)

3) Hospitalization to assess health status (e.g., routine colonoscopy)

The causal relationship with protocol treatment will be classified into the following three categories:

(1) Related: A causal relationship with protocol treatment is reasonably considered present, and the event is not considered attributable to worsening of the underlying disease, a complication, another treatment, or another cause.

(2) Relationship cannot be ruled out: The causal relationship with protocol treatment is unclear. The event could also be explained by worsening of the underlying disease, a complication, another treatment, or another cause.

(3) Not related: There is no causal relationship with protocol treatment, and the event can clearly be explained by worsening of the underlying disease, a complication, another treatment, or another cause.

## **10.3. Expected Adverse Events**

As also described in Section 2.3.3, Gosain et al. reported that guidewire perforation of the cystic duct occurred in 3 of 19 patients (15.8%) in whom EGBS was attempted, and one of these patients required emergency cholecystectomy [16]. In a Japanese study, Ishii et al. reported guidewire perforation of the cystic duct in 1 of 32 patients (3.1%) in whom EGBS was attempted [15].

## **10.4. Measures to Be Taken if an Adverse Event Occurs**

**10.4.1. Measures for the Study Participant**

If an adverse event occurs, the principal investigator or subinvestigator will take appropriate measures as necessary to protect the participant's safety, including providing treatment and discontinuing administration of any applicable study medication. If treatment or another measure is required, the participant will be informed accordingly.

If an adverse event is ongoing at the final observation after completion of study treatment, the principal investigator or subinvestigator will continue follow-up until the participant returns to the baseline condition (grade) or becomes clinically stable.

**10.4.2. Assessment and Documentation**

For each adverse event, the principal investigator or subinvestigator will document in the source records (e.g., the medical record) the name of the event, date of onset, whether it was serious or nonserious, details of measures or treatment, and outcome (including the date of recovery if the event resolves or the date on which symptoms stabilize if they become fixed).

**10.4.3. Reporting of Adverse Events**

When an adverse event becomes known, the principal investigator or subinvestigator will report it to the study representative (or the study office) using the case report form. In accordance with the procedures of the research institution, the principal investigator will report the adverse event to the head of the research institution or other appropriate person as necessary.

**10.4.4. Reporting of Serious Adverse Events**

When a serious adverse event becomes known, the subinvestigator will promptly report it to the principal investigator in accordance with the procedures established at that institution. After reporting the serious adverse event to the head of the affiliated research institution, the principal investigator will promptly report it to the study representative (or the study office) and share the information with the subinvestigators.

The study representative will seek the opinion of the ethics review committee regarding assessment of the adverse event, whether the study may continue, and whether the study should be modified, and will take any necessary measures.

The study representative (or the study office) will report an adverse event received from a principal investigator as promptly as possible to the principal investigators at the other research institutions where the event did not occur.

In accordance with the procedures of their respective research institutions and in light of the opinion of the ethics review committee, the study representative and the principal investigator at each institution will report the event to the head of their institution and share the information with the subinvestigators.

# **11. Efficacy and Safety Evaluation Committee**

No efficacy and safety evaluation committee will be established for this study.

# **12. Anticipated Benefits and Disadvantages Associated with Study Participation**

## **12.1. Anticipated Benefits**

Participation in this study will not provide a direct benefit to participants. Participation may contribute to future advances in medical care.

## **12.2. Burden and Anticipated Risks**

All devices used in this study are approved and covered by the national health insurance system for the target disease; therefore, participation in the study will not impose any additional financial burden compared with routine care. Adverse events that may occur as a result of participation are described in Section 10.3, "Expected Adverse Events." If an adverse event occurs, the principal investigator or subinvestigator will take appropriate action in accordance with Section 10.4, "Measures to Be Taken if an Adverse Event Occurs."

# **13. Procedures for Obtaining Informed Consent**

This study involves intervention and invasiveness and will newly obtain samples and information from participants for research purposes. It falls under Part 8, Section 1(1) of the Ethical Guidelines for Life Science and Medical Research Involving Human Subjects.

Before a patient participates in the study, the principal investigator or subinvestigator will provide a full explanation of the following matters using the participant information sheet, informed consent form, and consent withdrawal form approved by the Ethics Review Committee (see Section 20.2). Written consent to participate will be obtained after confirming that the patient has adequately understood the information.

As a rule, the following matters will be explained to the participant or other appropriate person when informed consent is obtained. This does not apply to matters for which the head of the research institution has granted permission after considering the opinion of the ethics review committee.

1. That permission to conduct the study has been obtained

2. The name of the research institution and the name of the principal investigator

3. The purpose and significance of the study

4. The methods and duration of the study

5. The reason the person was selected as a study participant

6. The burden on participants and the anticipated risks and benefits

7. Whether compensation is available for health injury and, if so, its details

8. The right to withdraw consent

9. That the participant will not be disadvantaged

10. Handling of personal information

11. Methods for storage and disposal of samples and information

12. Secondary use of samples and information

13. Handling of results obtained from the study

14. Costs and remuneration

15. Funding sources and conflicts of interest

16. Other treatment options

17. Provision of medical care after completion of the study

18. Methods for public disclosure of study information

19. The right to inspect study materials

20. Access to samples and information by relevant persons

21. Contact information for inquiries

## **13.1. Handling of Consultations from Participants and Related Persons**

The principal investigator or subinvestigator will respond to consultations from participants or related persons. If the appropriate response is unclear, the investigator will consult the study office in accordance with the nature of the inquiry before responding.

## **13.2. Obtaining Informed Consent from a Legally Authorized Representative**

Because of the characteristics of the target disease, many potential participants will be older adults, and some may lack decision-making capacity. If a potential participant is objectively judged to lack the capacity to provide informed consent, written consent will be obtained from a legally authorized representative using the participant information and consent documents approved by the ethics review committee. A legally authorized representative may be the participant's spouse, a relative within the second degree of kinship, or a cohabiting relative, excluding minors. Nevertheless, the study will also be explained to the participant in readily understandable language, according to the items in the participant information and consent documents and in a manner appropriate to the participant's level of understanding, and the participant's consent or assent will be obtained.

## **13.3. Obtaining Informed Assent**

Not applicable because this study includes adult patients only.

## **13.4. Research Conducted under Part 8, Section 8 of the Ethical Guidelines**

Not applicable in this study.

# **14. Site Registration and Participant Registration**

Site and participant registration will be centralized through the Registration Center (responsible department: Department of Gastroenterology and Hepatology, Yokohama City University Hospital). Participant registration may begin at a research institution after its site registration is complete.

## **14.1. Registration Center**

Person in charge: Shin Yagi

Institution/department: Department of Gastroenterology and Hepatology, Yokohama City University Hospital

Telephone: +81-45-787-2800

Fax: +81-45-787-2931

Email: t216074f@yokohama-u.ac.jp

Hours: Weekdays, 9:00 to 16:00 (closed on weekends, national holidays, and during the year-end/New Year holidays)

## **14.2. Site Registration**

After approval by the ethics review committee (see Section 20.2) and permission to conduct the study from the head of the research institution (see Section 20.3) have been obtained, the principal investigator at each institution will send a copy of the written permission from the head of the institution to the Registration Center. The Registration Center will register the site and notify the principal investigator when site registration is complete.

## **14.3. Participant Registration**

(1) After obtaining written consent from a potential participant, the principal investigator or subinvestigator will conduct the examinations and other assessments (screening) necessary to determine eligibility. The investigator will confirm that the participant meets all inclusion criteria and none of the exclusion criteria.

(2) The principal investigator or subinvestigator will complete the required items on the participant registration form and send it to the Registration Center by fax or email. Each institution will assign a participant identification code consisting of an arbitrary code that, by itself, contains no information that can identify a specific individual.

(3) The Registration Center will determine eligibility from the information recorded on the participant registration form and will enroll the participant in the study if the participant is judged eligible.

(4) The Registration Center will record on the registration confirmation form whether the participant has been enrolled in the study and will send the form to the principal investigator or subinvestigator by fax or email.

(5) The principal investigator or subinvestigator will retain the registration confirmation form.

# **15. Transfer of Samples and Information**

The participant registration form and all case report forms will be submitted as shown in the table below.

Source: Collaborating research institutions that register participants (Section 27.3)

Timing and methods for submitting the participant registration form and case report forms

| Document type | Submission timing | Submission method | Security measures | Destination |
| --- | --- | --- | --- | --- |
| Participant registration form | At participant registration | Fax or email | Double-check the recipient  Password-protect email attachments | Registration Center (Section 14.1) |
| Pre-treatment report | Within 30 days after registration |  |  | Study Office (Section 27.2) |
| ERCP | Within 30 days after the procedure |  |  |  |
| 30 days after ERCP | Within 30 days after this time point |  |  |  |
| At discontinuation |  |  |  |  |
| Follow-up form | April 30, 2028 to  May 31, 2028 |  |  |  |

# **16. Storage and Disposal of Samples and Information**

[Storage of Information]

The principal investigator will securely store and manage the documents and records concerning this study listed below in a lockable cabinet. Electronic data will be password-protected and stored on a computer isolated from the institutional local area network and the internet or on electronic recording media such as a USB drive; when not in use, these devices or media will be securely stored in a lockable cabinet. Records will be retained until the later of 5 years after the date on which completion of the study is reported or 3 years after the date on which final publication of the study results is reported. However, information processed so that individuals cannot be identified will be retained indefinitely after the end of this retention period because it may be used secondarily.

Information will be managed as follows, according to its type.

Types of information to be stored

• Information containing data that can identify a participant (including the list that links a participant identification code to personal information that can identify the participant for identification and inquiries [hereinafter, the "linkage table"], informed consent forms, and consent withdrawal forms)

• Information processed so that participants cannot be identified

• Study-related materials (including review documents such as the study protocol)

Each type of information will be stored as follows.

For information that can identify a participant, electronic data will be maintained under the responsibility of the principal investigator at each institution, with the recording media stored in a locked cabinet; paper records will also be securely stored in a locked cabinet. The originals of informed consent forms and consent withdrawal forms will be retained on paper, while electronic data scanned into the electronic medical record will be treated as copies.

Information processed so that participants cannot be identified will be stored under the responsibility of the principal investigator at each institution on password-protected computers or recording media such as USB drives.

Information collected by the study representative will be stored in the Department of Gastroenterology and Hepatology, Yokohama City University Hospital, on password-protected computers or recording media such as USB drives. Case report forms submitted on paper will be filed and stored in a locked cabinet.

Documents and records for which the retention period has elapsed will be disposed of with the utmost care to prevent disclosure of personal or confidential information. Electronic information will be permanently deleted, and paper records will be shredded and discarded. (Person responsible for management: the responsible person at each institution)

1) Study protocol

2) Participant information sheet, informed consent form, and consent withdrawal form

3) Participant registration form

4) Informed consent form (signed original)

5) Case report form (copy)

6) Linkage table

7) Documents submitted to the ethics review committee

8) Notification of the ethics review committee's decision and written permission to conduct the study from the head of the research institution

9) Monitoring reports

10) Other documents or records related to this study

## **16.1. Secondary Use of Samples and Information**

Samples and information obtained in this study will be stored and may be used secondarily for research with other purposes in the future or provided to third parties, including research institutions and researchers in Japan or other countries. Although the purposes of such secondary use and the recipients cannot currently be specified, participants will be informed in advance, to the extent foreseeable when consent is obtained, and their consent will be obtained.

If samples or information obtained in this study are used for research with another purpose in the future, a new study protocol will be prepared and approval from an ethics review committee and permission from the head of the research institution will be obtained. Secondary use will occur only after written consent has been obtained, the participant has been notified, or a public information notice has been posted and an opportunity to opt out has been assured, as applicable.

# **17. Handling of Personal Information**

A participant identification code will be assigned when each patient is registered in this study. The code will consist of numbers, symbols, or other characters unrelated to information that could identify a specific person, such as initials or a medical-record identification number. The participant identification code will be used on the registration form, case report forms, and other study documents so that the individual cannot be identified from those documents. The principal investigator will prepare a linkage table containing the participant's name, medical-record identification number, and other information so that the participant can be identified from the processed information when necessary, and will securely store and manage the table to prevent external disclosure. (Person responsible for management: the principal investigator at each collaborating research institution)

This is a multicenter collaborative study conducted at four institutions, including Yokohama City University Hospital. Each research institution will appropriately manage its linkage table, which will not be provided outside the institution. When a participant registration form, case report form, test data, or other information is provided outside the relevant institution, the participant identification code or registration number will be used so that the participant cannot be identified; only information processed or managed so that it is not immediately apparent which participant it concerns will be transferred. Personal information and related data in this study will be handled in compliance with this protocol and with each participating institution's procedures for handling personal information and related data in clinical research.

# **18. Handling of Study Results Relating to Participants (Including Incidental Findings)**

Results arising from an individual participant's participation in the study will be explained to that participant as part of clinical care.

This study is not expected to yield important findings concerning a participant's health, heritable genetic characteristics, or similar matters. However, if examinations or other procedures provide information, including incidental findings, that may have a serious effect on a participant's health, the principal investigator or subinvestigator will explain the information to the participant and take appropriate measures, such as providing treatment or another intervention.

Information will not be disclosed if the participant has indicated that disclosure is not desired. However, if a result is found to have a serious effect on the life of the participant or a blood relative and an effective response is available, the principal investigator will report it to the study representative. In deciding whether, how, and what to explain to the participant or other relevant person, the study representative will consider the following matters and seek the opinion of the ethics review committee:

1. • The effect on the life of the participant or the participant's blood relatives
2. • The availability of effective treatment and the participant's health status
3. • The possibility that a blood relative of the participant has the same disease or condition
4. • The information provided during informed consent regarding disclosure of results

After considering the opinion of the ethics review committee, the principal investigator and subinvestigators will provide a full explanation to the participant or other relevant person and confirm the participant's wishes. If the participant still does not wish to receive the information, it will not be disclosed.

## **18.1. Genetic Counseling System**

Not applicable because no genetic analysis will be performed in this study.

# **19. Protocol Compliance, Deviations, and Amendments**

## **19.1. Protocol Compliance**

The principal investigator or subinvestigator must not deviate from or modify the study protocol without the prior agreement of the study representative and prior written approval based on review by the ethics review committee.

## **19.2. Protocol Deviations**

1) When medically unavoidable, such as when necessary to eliminate an immediate hazard to a participant, the principal investigator or subinvestigator may deviate from or modify the study protocol before obtaining the prior agreement of the study representative and prior approval of the ethics review committee. In such a case, the principal investigator or subinvestigator will promptly submit the nature of and reason for the deviation or modification, together with a proposed protocol amendment if one is necessary, to the ethics review committee through the study representative, obtain its approval, and obtain permission from the head of the research institution.

2) If a protocol deviation occurs, the principal investigator or subinvestigator will prepare a complete record of the deviation and the reason for it. The principal investigator must report the deviation to the head of the research institution and the study representative and will retain a copy of the deviation record.

## **19.3. Procedures for Protocol Amendments**

The study protocol will be amended according to the following procedures:

1) If the study representative determines that an amendment is necessary, the study representative will provide the principal investigators with the proposed protocol amendment and any other necessary materials and information.

2) The study representative will allow the principal investigators sufficient time to carefully review the proposed protocol amendment and other materials and information provided under the preceding paragraph and to discuss them with the study representative.

3) After obtaining the agreement of the principal investigators, the study representative will complete the amendment procedures in accordance with Sections 20.2 and 20.3.

# **20. Procedures for Conducting the Study**

## **20.1. Applicable Rules and Guidelines**

This study will be conducted in accordance with the ethical principles of the Declaration of Helsinki and the Ethical Guidelines for Life Science and Medical Research Involving Human Subjects. The principal investigators and subinvestigators will conduct the protocol treatment in compliance with this study protocol.

## **20.2. Submission to the Ethics Review Committee**

Before conducting this study, the study representative will coordinate in advance with the relevant research institutions and obtain approval through centralized review by a single ethics review committee. Amendments or revisions to the protocol or related documents will also undergo centralized review and approval by the ethics review committee to which the initial review was submitted.

A collaborating research institution that does not participate in the centralized review will use the materials approved through the central review at the lead institution to undergo review and obtain approval from its own ethics review committee. The principal investigator at that institution will submit the institution's notice of review results to the study representative and report the outcome of the review.

## **20.3. Permission from the Head of Each Research Institution**

The study representative will share with the principal investigators at the collaborating research institutions the centralized review outcome, records showing the review process and attendance of the ethics review committee members, and the approved materials. Using these materials, the principal investigator at each institution will obtain permission to conduct the study from the head of that institution. Permission from the head of the institution will be obtained not only after the initial review but each time an amendment to the study undergoes review by the ethics review committee.

At a collaborating research institution that does not participate in centralized review, permission from the head of the institution will be obtained after approval by that institution's ethics review committee. After permission is obtained, the written permission will be submitted to the study representative and reported.

## **20.4. Periodic Reports**

The study representative and the principal investigator at each collaborating research institution will report the status of the study to the ethics review committee and the head of each research institution at least annually, or more frequently if requested by the ethics review committee.

## **20.5. Suspension or Termination of the Study**

If an investigator or other study personnel obtains information considered likely to affect continuation of the study, the information will be reported to the principal investigator at that institution, who will report it to the study representative and the head of the institution. The study representative will report it to the head of the lead institution and share the information with the principal investigators at all participating institutions, each of whom will report it to the head of their institution.

The study representative will suspend or terminate the study or amend the study protocol as necessary. If instructed by the head of a research institution to suspend or terminate the study, the study representative will respond promptly and share the information with all research institutions according to the procedure above; each principal investigator will report it to the head of their institution.

If the study is terminated or suspended, regardless of the reason, the investigators will promptly notify the participants, provide appropriate treatment, and perform examinations or other assessments necessary to confirm participant safety. The head of the research institution will also be notified in writing.

(1) The study treatment is found unlikely to be effective, a safety concern becomes evident, or continuation of the study is judged no longer meaningful.

(2) Completion of the study is judged difficult because of markedly delayed enrollment, frequent protocol deviations, or another reason.

(3) A serious noncompliance event affecting conduct of the study occurs.

## **20.6. Completion of the Study**

When the study is completed, including when it has been terminated with no prospect of resumption, the study representative will notify all principal investigators in writing and provide a summary of the study results. Without delay, the study representative and the principal investigator at each collaborating research institution will report completion of the study and a summary of its results to the ethics review committee and the head of the research institution.

# **21. Funding Sources and Financial Relationships, Institutional Conflicts of Interest and Individual Financial Interests, and Investigators' Conflicts of Interest**

## **21.1. Funding Sources and Financial Relationships**

This study will be conducted using basic research funds of the Department of Gastroenterology and Hepatology, Yokohama City University School of Medicine. It is conducted from a medical perspective and is not intended to benefit or provide preferential treatment to any particular company or organization. No financial support is received from any particular company or organization.

## **21.2. Conflicts of Interest**

**21.2.1. Conflict of Interest of the Study Representative**

Takayuki Oda, the study representative, has no conflict of interest to disclose.

**21.2.2. Conflicts of Interest of Principal Investigators, Subinvestigators, and Other Study Personnel**

Conflicts of interest of principal investigators and subinvestigators will be appropriately managed at each research institution in accordance with that institution's conflict-of-interest policies and regulations. The status of conflict-of-interest management will be reported to the study representative, and any change in the reported information will also be reported.

# **22. Costs and Remuneration for Study Participants**

Of the costs incurred in conducting this study, medical expenses for individual patients, including consultation fees, hospitalization costs, medication costs, and examination fees, will be paid by the participant to the extent of the participant's usual copayment because all treatment performed in this study is covered by national health insurance. Participation will not impose any additional financial burden on participants. No monetary or nonmonetary remuneration, expense allowance, or other payment will be made to participants for taking part in this study.

# **23. Compensation for Health Injury**

If a participant experiences a health injury caused by participation in this study, the principal investigator and subinvestigators will provide appropriate treatment and take any other necessary measures. Such treatment will be provided under national health insurance, and the participant will pay the applicable copayment.

Because commercially available drugs will be used within the indications, effects, dosages, administration methods, and other precautions described in their package inserts, a drug-related adverse reaction resulting in death or a grade 1 or 2 residual disability may be eligible for relief benefits under the Relief System for Adverse Drug Reactions. Examinations performed in this study are the same as those performed in routine care. For these reasons, clinical research liability insurance will not be obtained for this study.

# **24. Monitoring and Auditing**

## **24.1. Monitoring**

Monitoring is an investigation performed by a person designated by the principal investigator to ensure that the study is conducted appropriately by assessing its progress and whether it is being conducted in accordance with the study protocol, applicable procedures, relevant laws and regulations, and relevant guidelines.

(1) Monitoring Methods and Personnel

Central monitoring will be performed by the Data Center. Each institution will also conduct a self-inspection and document that eligibility and informed-consent requirements have been checked.

(2) Central Monitoring
1) Central monitoring will be based on data collected in the case report forms.
2) Based on the results of central monitoring, queries will be issued to verify the recorded data, including possible transcription errors and requests to add or complete missing data, and responses will be obtained. If necessary after reviewing the responses, the relevant site personnel, such as investigators, clinical research coordinators, or other research assistants, will be contacted. Contact may be made by telephone, fax, web conference, or another method, but participant-identifying information will be masked regardless of the method used.
3) Monitoring results will be documented in a monitoring report.
(3) Monitoring Items

1) Participant enrollment status and informed-consent status

2) Appropriateness of participant enrollment

3) Summary of baseline factors and treatment

4) Protocol compliance

5) Reliability of information recorded in the case report forms

6) Appropriate documentation and reporting of adverse events

7) Registration and study-completion status in the public database

(4) Submission of Monitoring Reports

The Data Center will document the monitoring results in a monitoring report and submit it to the principal investigator within 2 weeks after monitoring. Monitoring will be conducted once per year.

## **24.2. Auditing**

No audit will be conducted in this study.

# **25. Public Disclosure of Study Information and Publication of Results**

## **25.1. Study Registration**

Before the study begins, it will be registered in the database maintained by the Ministry of Health, Labour and Welfare (jRCT: Japan Registry of Clinical Trials). The registration will be updated as appropriate in response to protocol amendments and study progress. The study results will be registered when the study is completed.

## **25.2. Publication of Study Results**

After completion of the study, the findings will be promptly disclosed through presentation at a scientific meeting or submission of a manuscript. Before disclosure, necessary measures will be taken to protect the human rights of participants and related persons and the rights and interests of investigators and related persons. Presenters at scientific meetings and the first author of publications will be determined through discussion. Presenters and the first author must obtain review and approval from the study representative before presentation or submission. After the final publication, the head of every research institution will be notified.

# **26. Ownership of Study Results (Intellectual Property Rights)**

If intellectual property rights, such as patent rights, arise from the results of this study, those rights will, in principle, belong to Yokohama City University.

# **27. Study Organization**

## **27.1. Study Representative**

Research institution: Yokohama City University Hospital

Address: 3-9 Fukuura, Kanazawa-ku, Yokohama, Kanagawa 236-0004, Japan

Telephone: +81-45-787-2800

Name of study representative: Takayuki Oda

Affiliation of study representative: Department of Gastroenterology and Hepatology, Yokohama City University Hospital

Role: Study conception and overall coordination

## **27.2. Study Office**

Name: Takayuki Oda

Affiliation: Department of Gastroenterology and Hepatology, Yokohama City University Hospital

Address: 3-9 Fukuura, Kanazawa-ku, Yokohama, Kanagawa 236-0004, Japan

Telephone: +81-45-787-2800

Role: Communication and coordination with the collaborating research institutions

## **27.3. Collaborating Research Institutions and Principal Investigators**

1) Collaborating research institutions that will register participants

Role: Participant registration and implementation of protocol treatment

| Research institution | Head of institution | Department | Principal investigator |
| --- | --- | --- | --- |
| National Hospital Organization Yokohama Medical Center  3-60-2 Harajuku, Totsuka-ku, Yokohama, Kanagawa 245-8575, Japan  +81-45-851-2621 (main) | Makoto Ujihara | Department of Gastroenterology | Shinichi Nihei |
| Chigasaki Municipal Hospital  5-15-1 Honson, Chigasaki, Kanagawa 253-0042, Japan  +81-467-52-1111 (main) | Kiyoshi Fujinami | Department of Gastroenterology | Takamitsu Sato |
| Yokohama Rosai Hospital, Japan Organization of Occupational Health and Safety  3211 Kozukue-cho, Kohoku-ku, Yokohama, Kanagawa 222-0036, Japan  +81-45-474-8111 (main) | Yoji Mikami | Department of Gastroenterology | Masato Suzuki |

## **27.4. Cooperating Institutions and Responsible Persons**

None

## **27.5. Lead Statistician**

Name: Satoru Shinoda

Affiliation: Department of Biostatistics, Yokohama City University School of Medicine

Address: 3-9 Fukuura, Kanazawa-ku, Yokohama, Kanagawa 236-0004, Japan

Email: secbios@yokohama-cu.ac.jp

Role: Statistical analysis

## **27.6. Data Center/Data Management Lead**

Name: Yusuke Kurita

Affiliation: Department of Gastroenterology and Hepatology, Yokohama City University Hospital

Address: 3-9 Fukuura, Kanazawa-ku, Yokohama, Kanagawa 236-0004, Japan

Telephone: +81-45-787-2800

Role: Data management

## **27.7. Monitoring Lead**

Name: Naho Kobayashi

Affiliation: Department of Gastroenterology and Hepatology, Yokohama City University Hospital

Address: 3-9 Fukuura, Kanazawa-ku, Yokohama, Kanagawa 236-0004, Japan

Telephone: +81-45-787-2800

Role: Monitoring management

## **27.8. Audit Lead**

None

## **27.9. External Contractors**

None

# **28. References**

1. National Cancer Center Japan. Cancer Information Service. 2020 [cited November 10, 2024]. Available from: https://ganjoho.jp/reg_stat/statistics/stat/summary.html.

2.	Ishii, T., et al., *Risk factors for early and late cholecystitis after covered metal stent placement for distal biliary obstruction.* J Hepatobiliary Pancreat Sci, 2023. **30**(10): p. 1180-1187.

3.	Nakai, Y., et al., *International consensus statements for endoscopic management of distal biliary stricture.* J Gastroenterol Hepatol, 2020. **35**(6): p. 967-979.

4.	Inamdar, S., et al., *Comparison of Adverse Events for Endoscopic vs Percutaneous Biliary Drainage in the Treatment of Malignant Biliary Tract Obstruction in an Inpatient National Cohort.* JAMA Oncol, 2016. **2**(1): p. 112-7.

5.	Wang, D.H., et al., *Comparative Analysis of ERCP and PTBD for Biliary Interventions for Readmission Rates and Patient Outcomes.* J Clin Gastroenterol, 2024.

6.	Paik, W.H., et al., *EUS-Guided Biliary Drainage Versus ERCP for the Primary Palliation of Malignant Biliary Obstruction: A Multicenter Randomized Clinical Trial.* Am J Gastroenterol, 2018. **113**(7): p. 987-997.

7.	Bang, J.Y., et al., *Stent placement by EUS or ERCP for primary biliary decompression in pancreatic cancer: a randomized trial (with videos).* Gastrointest Endosc, 2018. **88**(1): p. 9-17.

8.	Park, J.K., et al., *Efficacy of EUS-guided and ERCP-guided biliary drainage for malignant biliary obstruction: prospective randomized controlled study.* Gastrointest Endosc, 2018. **88**(2): p. 277-282.

9.	Kaassis, M., et al., *Plastic or metal stents for malignant stricture of the common bile duct? Results of a randomized prospective study.* Gastrointest Endosc, 2003. **57**(2): p. 178-82.

10.	Davids, P.H., et al., *Randomised trial of self-expanding metal stents versus polyethylene stents for distal malignant biliary obstruction.* Lancet, 1992. **340**(8834-8835): p. 1488-92.

11.	Dumonceau, J.M., et al., *Biliary stenting: indications, choice of stents and results: European Society of Gastrointestinal Endoscopy (ESGE) clinical guideline.* Endoscopy, 2012. **44**(3): p. 277-98.

12.	Isayama, H., et al., *A prospective randomised study of "covered" versus "uncovered" diamond stents for the management of distal malignant biliary obstruction.* Gut, 2004. **53**(5): p. 729-34.

13.	Martinez, N.S., et al., *Evaluation of post-ERCP pancreatitis after biliary stenting with self-expandable metal stents vs. plastic stents in benign and malignant obstructions.* Endosc Int Open, 2021. **9**(6): p. E888-E894.

14.	Jung, J.H., et al., *Identification of risk factors for obstructive cholecystitis following placement of biliary stent in unresectable malignant biliary obstruction: a 5-year retrospective analysis in single center.* Surg Endosc, 2021. **35**(6): p. 2679-2689.

15.	Ishii, T., et al., *Prophylactic endoscopic gallbladder stent placement for cholecystitis after covered metal stent placement for distal biliary obstruction (with video).* Gastrointest Endosc, 2023. **98**(1): p. 36-42 e1.

16.	Gosain, S., et al., *Gallbladder stent placement for prevention of cholecystitis in patients receiving covered metal stent for malignant obstructive jaundice: a feasibility study.* Dig Dis Sci, 2010. **55**(8): p. 2406-11.

17.	Watanabe, M., et al., *Cholecystitis after Placement of Covered Self-Expandable Metallic Stents in Patients with Distal Malignant Biliary Obstructions.* Clin Endosc, 2021. **54**(4): p. 589-595.

18.	Yokoe, M., et al., *Tokyo Guidelines 2018: diagnostic criteria and severity grading of acute cholecystitis (with videos).* J Hepatobiliary Pancreat Sci, 2018. **25**(1): p. 41-54.

19.	Committee, A.S.o.P., et al., *Guidelines for privileging, credentialing, and proctoring to perform GI endoscopy.* Gastrointest Endosc, 2017. **85**(2): p. 273-281.

20.	Cotton, P.B., et al., *A lexicon for endoscopic adverse events: report of an ASGE workshop.* Gastrointest Endosc, 2010. **71**(3): p. 446-54.
