# Supplementary material for: Prophylactic endoscopic transpapillary gallbladder stenting at fully covered self-expandable metal stent placement to prevent cholecystitis in malignant distal biliary obstruction: A multicenter prospective single-arm study protocol (PROTECT study)
Source: PLoS One. 2026 Oct 29;21(10):e0361468. doi: 10.1371/journal.pone.0361468 (PMC13718957; doi:10.1371/journal.pone.0361468)
Supplement: Supplementary file 4 [file pone.0361468.s005.docx]

**Response to the Editor and Reviewers**

**Manuscript title:**

Prophylactic endoscopic transpapillary gallbladder stenting at fully covered self-expandable metal stent placement to prevent cholecystitis in malignant distal biliary obstruction: A multicenter prospective single-arm study protocol (PROTECT study)

**Dear Editor and Reviewers,**

Thank you for your careful and constructive review of our manuscript. We have revised the manuscript in accordance with the comments and believe that the revisions have improved its clarity and scientific rigor. The reviewers’ comments are reproduced below, followed by our responses and the corresponding changes in the revised manuscript. Page and line numbers refer to the revised manuscript.

We sincerely appreciate the time and effort devoted to evaluating our work.

**Journal Requirements:**
1. Please ensure that your manuscript meets PLOS ONE's style requirements, including those for file naming.

**Response:** Thank you. We reviewed the revised manuscript against the PLOS ONE style and file-preparation requirements. The clean manuscript, revised manuscript with tracked changes, and response letter will be uploaded as separate files with the requested file designations. We also standardized the references and Supporting Information legends to match PLOS ONE style.

2. Your ethics statement should only appear in the Methods section of your manuscript. If your ethics statement is written in any section besides the Methods, please delete it from any other section.

**Response:** Thank you for this comment. We confirm that the ethics statement appears only in the Materials and Methods section, under the subsection “Ethics and dissemination.” No ethics statement is included in any other section of the manuscript. Therefore, no revision was required in response to this comment.

3. Please provide a complete Data Availability Statement in the submission form, ensuring you include all necessary access information or a reason for why you are unable to make your data freely accessible. If your research concerns only data provided within your submission, please write "All data are in the manuscript and/or supporting information files" as your Data Availability Statement.

**Response:** Thank you. Because this article reports a study protocol and no datasets were generated or analyzed for the current manuscript, we entered the complete Data Availability Statement in the submission form and revised the statement in the manuscript accordingly.

**Changes in the manuscript (Data availability statement; Lines 20–22):**
“For Study Protocols: No datasets were generated or analyzed during the current study. All relevant data from this study will be made available upon study completion.”

4. Thank you for providing your underlying data as Supporting Information. We note that the data set contains text or data that is not in English. Please note that PLOS is an English-language publisher, so we require data sets to be provided in English as well. Please upload an English-language version of your data set.

**Response:** Thank you for this comment. We apologize for the confusion. S2 File is not an underlying dataset; it is protocol version 1.3 in the original Japanese. The corresponding English translation is provided as S3 File. We revised the Supporting Information legends to make the relationship between these files explicit.

**Changes in the manuscript (Supporting information; Lines 608–610):**
“S1 File. SPIRIT checklist.
S2 File. Study protocol, version 1.3 (original Japanese-language version).
S3 File. Study protocol, version 1.3 (English translation of S2 File).”

Thus, the revised submission includes both the original Japanese-language protocol and a clearly identified English-language translation.

5. If the reviewer comments include a recommendation to cite specific previously published works, please review and evaluate these publications to determine whether they are relevant and should be cited. There is no requirement to cite these works unless the editor has indicated otherwise.

**Response:** Thank you. We reviewed the publications and guidance mentioned in the reviewer comments and evaluated their relevance to the study protocol. Citations were used where they directly support the response or manuscript; no publication was cited solely because it was mentioned by a reviewer.

**Reviewer #1: Comment 1.**

Exclusion criterion 3 excludes patients if pre-ERCP imaging suggests FCSEMS placement is "unlikely to obstruct the cystic duct orifice, as judged by the site principal investigator." Because this evaluation is highly subjective, please clarify if any specific anatomical parameters (e.g., distance from the tumor margin to the cystic duct confluence) will be used to standardize this decision across the four participating institutions to minimize selection bias (pp. 12-13).

**Response:**

Thank you for this important comment. We agree that the original wording allowed subjective judgment and could result in inconsistent patient selection across the participating institutions. We considered using the distance between the proximal margin of the malignant biliary stricture and the cystic duct orifice as an anatomical parameter. However, no validated cutoff has been established, and this distance cannot always be measured reliably because the cystic duct orifice and the precise margin of the malignant stricture may be difficult to identify on pre-ERCP imaging. We therefore did not adopt a fixed distance cutoff.

We have clarified that patients will be excluded under this criterion only when both of the following conditions are met on pre-ERCP CT and/or MRI: (i) the cystic duct orifice is clearly identifiable, and (ii) the proximal end of the planned FCSEMS is expected to remain distal to the cystic duct orifice without overlapping it. If either condition cannot be determined, the patient will not be excluded on the basis of this criterion. The imaging assessment will be performed independently by two experienced endoscopists at each participating institution, and exclusion will require agreement between both endoscopists. The manuscript and study protocol have been revised accordingly.

**Changes in the manuscript (Methods, Exclusion criteria; Lines 122–129):**
“Patients will be excluded only when both of the following conditions are met on pre-ERCP CT and/or MRI: (i) the cystic duct orifice is clearly identifiable, and (ii) the proximal end of the planned FCSEMS is expected to remain distal to the cystic duct orifice without overlapping it. If either condition cannot be determined, the patient will not be excluded on the basis of this criterion. No fixed cutoff will be applied for the distance between the proximal margin of the malignant biliary stricture and the cystic duct orifice. The imaging assessment will be performed independently by two experienced endoscopists at each participating institution, and exclusion will require agreement between both endoscopists;”

**Changes in the manuscript (Discussion, Limitations; Lines 464–468):**
“Third, although prespecified imaging criteria and independent assessment by two experienced endoscopists will be used, no blinded central imaging review or formal assessment of interobserver agreement is planned. Some site-level interobserver variability may therefore remain and may limit the reproducibility and external validity of exclusion criterion 3.”

**Reviewer #1: Comment 2.**

The primary endpoint is evaluated among all patients in whom ETGBS is "attempted" (p. 16). Please explicitly define what constitutes a valid "attempt" (e.g., does it require intentional guidewire cannulation of the cystic duct orifice, or simply the visualization of the orifice during ERCP?) (pp. 15-16). This is critical for clear baseline registration and FAS population tracking (p. 19).

**Response:**

Thank you for this important comment. We agree that an explicit definition of an ETGBS attempt is necessary to ensure consistent procedural classification and tracking of the FAS population. In this study, an ETGBS attempt is defined as the initiation of intentional guidewire manipulation toward the visualized or presumed location of the cystic duct orifice, with the aim of advancing the guidewire through the cystic duct into the gallbladder. An ETGBS attempt will therefore be recorded regardless of whether the cystic duct orifice is visualized or guidewire cannulation of the cystic duct is successful. Visualization of the cystic duct orifice alone, without intentional guidewire manipulation, will not be considered an ETGBS attempt. Once such guidewire manipulation is initiated, the procedure will be classified as an ETGBS attempt regardless of its duration or technical outcome. We have added this definition to the Procedural details and Primary endpoint subsections and have revised the definition of the FAS accordingly. The corresponding definitions have also been added to the study protocol. Reasons for not initiating an ETGBS attempt after enrollment will be recorded and reported in the study flow diagram.

**Changes in the manuscript (Methods, Procedural details; Lines 169–174):**
“After cholangiography, the endoscopist will intentionally manipulate a guidewire toward the visualized or presumed location of the cystic duct orifice with the aim of advancing it through the cystic duct into the gallbladder. Initiation of this intentional guidewire manipulation will constitute an ETGBS attempt, regardless of whether the cystic duct orifice is visualized or guidewire cannulation of the cystic duct is successful. Visualization of the cystic duct orifice without intentional guidewire manipulation will not be considered an ETGBS attempt.”

**Changes in the manuscript (Methods, Primary endpoint; Lines 210–213):**
“An ETGBS attempt is defined as the initiation of intentional guidewire manipulation toward the visualized or presumed location of the cystic duct orifice with the aim of advancing the guidewire through the cystic duct into the gallbladder.”

**Changes in the manuscript (Methods, Statistical analysis; Lines 285–291):**
“The full analysis set (FAS) will serve as the primary analysis population and will include all enrolled participants in whom an ETGBS attempt is initiated and for whom at least one item of post-procedure outcome information is available, regardless of the technical success of ETGBS or subsequent treatment. Participants in whom an ETGBS attempt is not initiated, participants for whom no post-procedure outcome information is available, participants without baseline data, and participants with protocol violations that preclude inclusion in the FAS will be excluded. The number of excluded participants and the reasons for exclusion will be reported.”

The manuscript also clarifies that participants in whom biliary cannulation fails before initiation of an ETGBS attempt will not be included in the FAS but will be included in the safety analysis set, and that their number and the reasons for not initiating ETGBS will be reported (Lines 295–298).

**Reviewer #1: Comment 3.**

In the Sample Size section, you anticipate a 10% attrition rate (leaving 45 evaluable patients) (p. 18). Please clarify whether patients with technically failed ETGBS procedures are considered part of this 10% attrition or if they remain fully evaluable within the FAS group, as lines 179–180 state the primary endpoint covers all attempted cases (pp. 16, 18-19).

**Response:**

Thank you for this important comment. We confirm that technical failure of ETGBS is not included in the anticipated 10% attrition. The primary endpoint evaluates the strategy of attempting ETGBS and therefore includes both technically successful and technically failed procedures. Participants with technically failed ETGBS will remain in the FAS and will contribute to the primary analysis, provided that an ETGBS attempt, as explicitly defined in the revised manuscript, has been initiated and the required 30-day outcome data are available. The anticipated 10% of participants who may be unevaluable for the primary endpoint refers to withdrawal of consent, major protocol deviations, or unavailability of 30-day outcome data, and not to technical failure of ETGBS. We have clarified this distinction in the Sample Size and Statistical Analysis sections of the manuscript and in the corresponding sections of the study protocol.

**Changes in the manuscript (Methods, Sample size; Lines 262–268):**
“Allowing for 10% of enrolled participants to be unevaluable for the primary endpoint because of withdrawal of consent, major protocol deviations, or unavailability of 30-day outcome data, we anticipate that 45 patients will be evaluable for the primary analysis. Technical failure of ETGBS will not be considered attrition. Participants with technically unsuccessful ETGBS will remain evaluable for the primary endpoint, provided that an ETGBS attempt has been initiated and the primary endpoint can be ascertained through day 30 or until an earlier competing event.”

**Reviewer #1: Comment 4.**

Line 174 states that procedure time will be measured from scope insertion to scope withdrawal (p. 16). Since attempting ETGBS can significantly prolong ERCP procedures (p. 10), consider tracking the specific time spent solely on attempting the gallbladder stenting (up to the allowed 60 minutes) versus the time spent on the standard biliary stent placement (pp. 15-16).

**Response:**

Thank you for this valuable suggestion. We agree that total procedure time alone does not distinguish the additional procedural burden attributable to ETGBS from the time required for standard biliary stent placement. We have therefore added separate measurements of total procedure time, ETGBS-specific procedure time, and FCSEMS placement time. ETGBS-specific procedure time is defined as the interval from initiation of intentional guidewire manipulation toward the visualized or presumed location of the cystic duct orifice to either successful gallbladder stent placement or discontinuation of the ETGBS attempt. FCSEMS placement time is defined as the interval from initiation of advancement of the FCSEMS delivery system over the biliary guidewire to completion of stent deployment.

We have also clarified that the 60-minute threshold is measured from scope insertion and includes standard ERCP steps performed before the ETGBS attempt, such as biliary cannulation and cholangiography; it does not represent 60 minutes of ETGBS-specific guidewire manipulation. Furthermore, the ETGBS attempt may be discontinued earlier if continued manipulation is considered technically difficult or unsafe. The manuscript and study protocol have been revised accordingly.

**Changes in the manuscript (Methods, Procedural details; Lines 183–188):**
“The ETGBS attempt will be discontinued if ETGBS is not successfully completed within 60 minutes after scope insertion. The attempt may be discontinued earlier if the endoscopist considers further manipulation technically difficult or unsafe. The 60-minute limit refers to the total elapsed time from scope insertion and includes standard ERCP steps performed before the ETGBS attempt, such as biliary cannulation, cholangiography, and endoscopic sphincterotomy when required. It does not represent 60 minutes of ETGBS-specific guidewire manipulation.”

**Changes in the manuscript (Methods, Procedural details; Lines 189–194):**
“Total procedure time will be measured from scope insertion to scope withdrawal. ETGBS attempt time will be measured from the initiation of intentional guidewire manipulation toward the visualized or presumed location of the cystic duct orifice to either successful placement of the gallbladder stent or discontinuation of the ETGBS attempt. FCSEMS placement time will be measured from the initiation of advancement of the FCSEMS delivery system over the biliary guidewire to completion of FCSEMS deployment.”

**Reviewer #1: Comment 5.**

Lines 157–160 and 165–167 allow for various brands of guidewires, catheters, and plastic stents ("or equivalent") (p. 15). Because different guidewire stiffness profiles and stent designs (5-Fr vs. 7-Fr) can impact technical success or cystic duct perforation rates (pp. 10, 15), please state whether these device specifics will be recorded and explored in post hoc analyses (p. 20).

**Response:**

Thank you for this important comment. We have clarified that the plastic stents used in this study are limited to two prespecified devices: a 7-Fr Through & Pass double-pigtail stent and a 5-Fr IYO stent. The type and caliber of the plastic stent placed are recorded for each participant. The study protocol already specifies that, among patients with technically successful ETGBS, the incidence of acute cholecystitis will be summarized separately according to the plastic stent type and caliber, and we have now added this information to the manuscript.

Although the guidewire and catheter models are not restricted to specific products, we have revised the manuscript and study protocol to specify that the manufacturer and model of all guidewires and catheters used during the ETGBS attempt will be recorded for each procedure. However, because the target sample size is limited to 50 participants and several guidewire and catheter models may be used, device-specific analyses according to guidewire or catheter model are not planned, as such analyses would yield small and difficult-to-interpret subgroups. We have clarified this point in the Statistical Analysis section.

**Changes in the manuscript (Methods, Procedural details; Lines 197–199):**
“The manufacturer and model of all guidewires and catheters used during the ETGBS attempt will be recorded for each procedure. The type and caliber of the gallbladder stent placed and the FCSEMS model used will also be recorded.”

**Changes in the manuscript (Methods, Secondary endpoints; Lines 236–239):**
“Among patients with technically successful ETGBS, the incidence of acute cholecystitis will also be summarized separately according to the type and caliber of the plastic stent placed (7-Fr Through & Pass double-pigtail stent or 5-Fr IYO stent).”

**Changes in the manuscript (Methods, Statistical analysis; Lines 346–349, 362–363):**
“The incidence will also be summarized descriptively according to the type and caliber of the gallbladder stent placed. Because gallbladder stent selection is not randomized, these descriptive summaries will not be interpreted as causal comparisons between the two stent types.”

“The guidewire and catheter models used will also be summarized descriptively; no formal device-specific comparisons are planned.”

**Reviewer #1: Comment 6.**

While you appropriately define the primary endpoint using the Tokyo Guidelines 2018 (TG18) for definite acute cholecystitis, you do not mention severity grading (pp. 16-17). Will the study capture and report the severity (Grade I, II, or III) of the cholecystitis events? This would add high-utility data regarding clinical impact.

**Response:**
Thank you for this valuable comment. We agree that the severity of acute cholecystitis is clinically important for evaluating the clinical impact of this complication. The study protocol already specifies that the severity of acute cholecystitis will be assessed according to TG18. We have now clarified in the manuscript that each definite acute cholecystitis event will be graded as Grade I (mild), Grade II (moderate), or Grade III (severe) according to TG18 and that the severity grades will be summarized descriptively.

**Changes in the manuscript (Methods, Primary endpoint; Lines 223–225):**
“The severity of every definite acute cholecystitis event occurring during follow-up will be graded as Grade I (mild), Grade II (moderate), or Grade III (severe) according to TG18.”

**Changes in the manuscript (Methods, Secondary endpoints; Lines 235–236):**
“The diagnosis and severity grading of acute cholecystitis will be performed as described for the primary endpoint according to TG18.”

**Changes in the manuscript (Methods, Study status and timeline; Lines 252–253):**
“Any episode of acute cholecystitis occurring during follow-up, including its TG18 severity grade, will be recorded.”

**Changes in the manuscript (Methods, Statistical analysis; Lines 361–362):**
“The TG18 severity grade and management of definite acute cholecystitis will be summarized descriptively.”

**Reviewer #1: Comment 7.**

If a patient experiences a failed ETGBS attempt and subsequently develops severe acute cholecystitis (pp. 23-24), is there a standardized management protocol across the four institutions (e.g., prioritizing EUS-guided drainage vs. percutaneous cholecystostomy) (p. 10)? Please clarify if rescue therapies will be tracked as a secondary safety outcome (p. 16).

**Response:**
Thank you for this comment. No standardized management algorithm or predefined priority among rescue interventions will be mandated across the four participating institutions. Management will be determined at each institution in accordance with TG18 and the patient’s clinical condition, irrespective of the technical outcome of ETGBS. All treatments provided for acute cholecystitis, including rescue interventions, will be recorded and summarized descriptively. We have clarified these points in the manuscript.

**Changes in the manuscript (Methods, Procedural details; Lines 202–206):**
“If acute cholecystitis develops during follow-up, management will be determined at each institution in accordance with Tokyo Guidelines 2018 (TG18) [22] and the patient’s clinical condition. No standardized rescue-treatment algorithm or predefined priority among interventions will be mandated across the participating institutions. All treatments provided for acute cholecystitis, including rescue interventions, will be recorded.”

**Changes in the manuscript (Methods, Statistical analysis; Lines 361–362):**
“The TG18 severity grade and management of definite acute cholecystitis will be summarized descriptively.”

**Reviewer #1: Comment 8.**

Secondary endpoint 4 evaluates the incidence of acute cholecystitis from day 31 until the end of follow-up (April 2028) (pp. 16-17). Given that the inclusion criteria require an expected survival of at least 4 months, please provide an estimate of the expected median follow-up duration per patient to help contextualize long-term stent patency (pp. 12, 21).

**Response:**
Thank you for this important comment. We agree that the observed follow-up duration is important for interpreting the incidence of acute cholecystitis occurring from day 31 onward. However, a reliable estimate of the median follow-up duration cannot be provided before study completion because the actual follow-up duration will vary according to the timing of enrollment, the type and stage of the underlying malignancy, disease progression, response to anticancer treatment, and survival. Furthermore, the eligibility requirement for an expected survival of at least 4 months represents a clinical assessment at enrollment and does not guarantee a fixed follow-up duration.

Although previous studies of therapeutic transpapillary gallbladder stenting in patients with established acute cholecystitis have reported favorable long-term clinical outcomes, their patient populations and treatment objectives differ substantially from those of the present study of prophylactic ETGBS in patients with malignant distal biliary obstruction. Therefore, the follow-up durations reported in those studies cannot be directly extrapolated to our study population. Similarly, an estimate based solely on an assumption of uniform enrollment would represent an artificial administrative estimate and could be misleading.

We have therefore not specified an expected median follow-up duration. Instead, the median observed follow-up duration from the day-31 landmark to the end of observation will be reported, and the late cholecystitis endpoint will be analyzed using a landmark time-to-event approach that accounts for competing events. We have clarified these points in the Statistical analysis subsection.

**Changes in the manuscript (Methods, Statistical analysis; Lines 350–361):**
“For definite acute cholecystitis occurring from day 31 onward, a landmark time-to-event analysis will be performed among participants who are alive and under follow-up at day 31 and have experienced neither definite acute cholecystitis nor a competing event before that time. Time will be measured from the day-31 landmark to the first definite acute cholecystitis event. Death before definite acute cholecystitis and cholecystectomy or gallbladder drainage performed for an indication other than definite acute cholecystitis will be treated as competing events. Cholecystectomy or gallbladder drainage performed for definite acute cholecystitis will be counted as an acute cholecystitis event. Participants without an event or competing event will be censored at the last follow-up assessment or the administrative end of follow-up on April 30, 2028, whichever occurs first. The cumulative incidence function will be estimated using the Aalen–Johansen method. The cumulative incidence curve and the median observed follow-up duration from the day-31 landmark to the end of observation will be reported.”

**Reviewer #1: Comment 9.**

As noted in your limitations, this single-arm study relies on historical thresholds (12.1%) for comparison (pp. 18, 24). Please detail how you plan to address confounding baseline variations (such as the presence of pre-existing gallstones or tumor invasion of the cystic duct) when interpreting the exploratory one-sample test results (pp. 19, 21-22).

**Response:**

Thank you for this important comment. We agree that differences in baseline risk factors between the present study population and the historical cohort may confound the comparison with the historical threshold of 12.1%. The one-sample test is a supplementary exploratory analysis intended to identify a preliminary signal of potential benefit and inform future comparative studies; it cannot provide confirmatory evidence of efficacy.

Available baseline information on factors potentially associated with acute cholecystitis after FCSEMS placement, including the presence of gallstones and tumor involvement of the cystic duct orifice, will be summarized descriptively where these factors can be assessed from the collected pre-ERCP imaging data. Where corresponding information is available in the historical study, differences in the distributions of these risk factors will be considered when interpreting the exploratory one-sample test.

Because the target sample size is 50 participants and the expected number of acute cholecystitis events is small, a multivariable-adjusted analysis or an adjusted comparison with the historical cohort would not be statistically robust and is therefore not planned. The results of the exploratory comparison will instead be interpreted in the context of the available baseline risk-factor information and the possibility of residual confounding. We have clarified the exploratory nature of the historical comparison and the potential for residual confounding in the Statistical Analysis and Discussion sections of the manuscript.

**Changes in the manuscript (Methods, Statistical analysis; Lines 309–311):**
“Baseline factors potentially associated with acute cholecystitis after FCSEMS placement, including gallstones and tumor involvement of the cystic duct orifice, will also be recorded and summarized descriptively.”

**Changes in the manuscript (Methods, Statistical analysis; Lines 323–331):**
“As a supplementary exploratory analysis, a one-sample test for a proportion will be performed using a null value of 12.1% and a single two-sided significance level of 10%. This analysis is intended to assess whether the observed incidence provides a preliminary signal relative to the historical benchmark and to inform the design of future comparative studies. Because of the single-arm design, the comparison will not by itself be interpreted as establishing a causal treatment effect or providing confirmatory evidence of efficacy. Differences in relevant baseline risk factors between the present study population and the historical cohort will be considered when interpreting the results, where corresponding historical data are available. No multivariable-adjusted analysis is planned because of the limited sample size and expected small number of events.”

**Changes in the manuscript (Discussion, Limitations; Lines 455–460):**
“Any comparison with a non-ETGBS strategy will rely on historical data and may be confounded by differences in patient characteristics, stent selection, and procedural techniques. Although relevant baseline risk factors will be described and considered when interpreting the historical comparison, residual confounding cannot be excluded. Therefore, the exploratory one-sample test will be interpreted as a hypothesis-generating comparison with the historical benchmark rather than as confirmatory evidence of treatment efficacy.”

**Reviewer #1: Comment 10.**

Line 248 mentions that no interim analyses are planned (p. 20). Since historical literature reports procedural risks like cystic duct perforation from ETGBS attempts (pp. 10, 22), please clarify if the central Data Center at Yokohama City University Hospital has specific "stopping rules" or safety thresholds that would trigger an unplanned safety review if procedural adverse events exceed acceptable baselines (p. 20).

**Response:**
Thank you for this important comment. The study protocol does not specify a fixed numerical stopping boundary for procedural adverse events, and the Data Center does not have independent authority to suspend or terminate the study. Given the small target sample size of 50 participants and the variability in the procedural adverse event rates reported in previous studies, a numerical threshold based on a small number of events could be unstable and potentially misleading.

Instead, safety oversight is event-driven. All adverse events will be recorded in the case report forms and reviewed through central monitoring by the Data Center at Yokohama City University Hospital. Serious adverse events will be reported promptly by the site principal investigator to the study representative and the relevant institutional authorities without waiting for the scheduled central monitoring. Any serious adverse event considered related or possibly related to the ETGBS attempt, including cystic duct perforation requiring unplanned invasive treatment, or any emerging pattern of procedural adverse events that raises concern regarding continued study safety, will trigger an ad hoc safety review by the study representative in consultation with the participating investigators and the Ethics Committee.

On the basis of this review, enrollment may be temporarily suspended, the study protocol may be modified, or the study may be terminated if continuation is considered unsafe. This event-driven safety review is separate from a planned interim analysis. We have clarified the safety monitoring and study suspension procedures in the Data Management and Monitoring subsection of the manuscript.

**Changes in the manuscript (Methods, Data management and monitoring; Lines 372–380):**
“The Data Center at Yokohama City University Hospital will conduct central monitoring to ensure data quality, oversee study conduct, and review all adverse events recorded in the case report forms. The Data Center monitors safety data but does not independently make decisions regarding study suspension, and no fixed numerical stopping boundary has been prespecified. Any serious adverse event related or possibly related to ETGBS, or any concerning pattern of procedural adverse events, including cystic duct perforation, will be promptly reported and trigger an ad hoc safety review by the study representative in consultation with the participating investigators and the Ethics Committee. Enrollment may be suspended, the protocol modified, or the study terminated if continuation is considered unsafe.”

**Reviewer #2: Comment 1.**

Exclusion criterion 3 rules out patients based on pre-ERCP imaging findings suggesting that FCSEMS placement is unlikely to obstruct the cystic duct orifice, as judged by the site principal investigator. This introduces substantial selection bias because it relies on an unquantified visual assessment by unblinded investigators. The protocol lacks standardized, reproducible radiological criteria (e.g., minimum tumor-margin distance from the cystic duct, insertion angle, or volumetric tumor burden) to objectively define the likelihood of obstruction. Furthermore, the lack of an independent, blinded central radiological review and formal evaluation of interobserver agreement (e.g., kappa or intraclass correlation coefficient) compromises the study’s external validity and cohort representativeness.

**Response:**

Thank you for this important comment. We agree that the original wording of exclusion criterion 3 involved an unquantified visual assessment and could have introduced selection bias. As described in our response to Reviewer 1, Comment 1, no validated radiological cutoff or quantitative parameter has been established for predicting whether FCSEMS placement will obstruct the cystic duct orifice. Moreover, this determination depends not only on individual anatomical parameters, such as distance, angle, or tumor burden, but also on the intended length and position of the FCSEMS. We therefore considered that adopting an arbitrary quantitative cutoff would not necessarily improve the validity or reproducibility of patient selection.

To minimize subjective exclusion, we revised exclusion criterion 3 to specify two conditions that must both be met: (i) the cystic duct orifice must be clearly identifiable on pre-ERCP CT and/or MRI, and (ii) the proximal end of the planned FCSEMS must be expected to remain distal to the cystic duct orifice without overlapping it. The assessment will be performed independently by two experienced endoscopists at each participating institution, and exclusion will require agreement between both assessors. Thus, if the assessments are discordant or the anatomical relationship is indeterminate, the patient will not be excluded on the basis of this criterion. This conservative approach is intended to reduce subjective exclusion and preserve cohort representativeness.

We acknowledge that a blinded central imaging review and formal assessment of interobserver agreement could provide additional information regarding reproducibility. However, these assessments were not prespecified, and the study was not designed or powered as an imaging reproducibility study. In addition, different pairs of endoscopists perform the assessments at the four participating institutions, which would make formal agreement estimates difficult to interpret. Therefore, a blinded central review and formal interobserver agreement analysis are not planned. This limitation has been explicitly acknowledged in the Discussion.

The relevant revisions to the Methods and Discussion are already described in our response to Reviewer 1, Comment 1. No additional changes were made in response to this comment.

**Reviewer #2: Comment 2.**
Inclusion criterion 4: “Expected survival of at least 4 months." Without the use of validated prognostic scoring systems, predicting survival in advanced MDBO is difficult. The protocol does not specify the quantification of this metric, ignoring existing standards such as ECOG performance status, TNM staging, or liver function scores (Child-Pugh/MELD). The use of subjective investigator discretion is likely to lead to heterogeneity among cohorts across participating centers. If institutions enroll patients with different disease aggressiveness who expire prior to the 30-day evaluation, the primary endpoint can be confounded due to survivorship bias. by survivorship bias.

**Response:**

Thank you for this important comment. We agree that prediction of individual survival in patients with MDBO is inherently imprecise and that an unstructured assessment could introduce interinstitutional variability. The 4-month criterion was adopted because it reflects the anticipated clinical indication for FCSEMS placement in patients expected to benefit from longer stent patency, rather than a score-derived prognostic endpoint.

The study includes MDBO caused by different malignant diseases with varying stages and treatment options, and no single prognostic scoring system has been validated to provide a uniform cutoff for 4-month survival across this heterogeneous population. ECOG performance status and tumor stage are relevant components of prognosis but do not independently provide such a cutoff, whereas Child-Pugh and MELD scores were developed primarily for patients with chronic liver disease and are not appropriate as universal prognostic tools in patients with malignant obstructive jaundice. We therefore did not introduce a fixed prognostic score threshold. Instead, we have clarified that expected survival will be assessed by the site principal investigator based on the type and extent of the underlying malignancy, performance status, organ function, and planned anticancer treatment.

In addition, inclusion criterion 4 is applied together with exclusion criterion 7, which excludes patients for whom follow-up at 30 days after FCSEMS placement is anticipated to be difficult. Therefore, patients who are expected at enrollment to have a survival of less than 4 months or for whom 30-day follow-up is expected to be unavailable will not be enrolled. Nevertheless, because survival prediction is inherently uncertain, a participant may unexpectedly die within 30 days. Such a participant will not be retrospectively excluded from the FAS solely because of death; the occurrence of acute cholecystitis up to the time of death and the death itself will be recorded and reported. Thus, the primary analysis will not be restricted to participants who survive to day 30. The manuscript has been revised accordingly.

**Changes in the manuscript (Methods, Inclusion criteria; Lines 109–111):**
“Expected survival of at least 4 months, as assessed by the site principal investigator based on the type and extent of the underlying malignancy, performance status, organ function, and planned anticancer treatment.”

**Changes in the manuscript (Methods, Primary endpoint; Lines 215–223):**
“Definite acute cholecystitis occurring within 30 days after FCSEMS placement will be counted as a primary endpoint event. Cholecystectomy or gallbladder drainage performed for definite acute cholecystitis will also be counted as an event. Participants who die without preceding definite acute cholecystitis or who undergo cholecystectomy or gallbladder drainage for an indication other than definite acute cholecystitis within 30 days will remain in the denominator and will not be counted as having the primary endpoint. The number of such participants, the timing and causes of death, and the timing and indications for these interventions will be reported separately.”

**Reviewer #2: Comment 3.**
The protocol allows for up to 60 minutes of ETGBS attempts before discontinuation. Such a duration poses serious safety issues and is inconsistent with the accepted interventional guidelines. ESGE (European Society of Gastrointestinal Endoscopy) defines difficult biliary cannulation as taking more than 5 minutes or requiring more than 5 papillary contacts. Since extensive papillary manipulation is the main independent risk factor for post-ERCP pancreatitis (PEP), an hour of cannulation attempts biases the risk of severe papillary trauma, massive local edema, and accidental pancreatic duct cannulation.

**Response:**

Thank you for this important comment. We agree that prolonged repeated manipulation of the major papilla and unintended pancreatic duct cannulation increase the risk of post-ERCP pancreatitis. However, we believe that an important distinction is required regarding the 5-minute criterion cited in the comment.

The ESGE criterion of more than 5 minutes or more than 5 contacts with the papilla defines difficult selective biliary cannulation through a native major papilla before access to the common bile duct is achieved. It is not a recommended 5-minute upper limit for the entire ERCP procedure or for guidewire cannulation of the cystic duct after biliary access has been established. In the present study, ETGBS is initiated only after transpapillary biliary cannulation and cholangiography. Subsequent guidewire manipulation is performed within the biliary system toward the cystic duct orifice and does not involve repeated contact with the major papilla. Therefore, the ESGE definition of difficult biliary cannulation cannot be directly applied as the time limit for the ETGBS attempt.

Moreover, the 60-minute threshold in this protocol represents the total elapsed time from scope insertion, not 60 minutes of ETGBS-specific guidewire manipulation. It includes scope advancement, biliary cannulation, cholangiography, and endoscopic sphincterotomy when required. In a previous study of ETGBD for acute cholecystitis, the median procedure time from scope insertion to successful stent deployment was 24 minutes, with an interquartile range of 8–52 minutes (Nishiguchi et al., Endosc Ultrasound. 2021;10:448–454). ETGBS may be more technically challenging in patients with MDBO because tumor-related biliary narrowing can restrict guidewire manipulation within the bile duct.

The 60-minute threshold is an upper limit and does not mandate continuation of the ETGBS attempt for 60 minutes. The attempt will be discontinued earlier whenever the endoscopist considers further manipulation technically difficult or unsafe, including when the cystic duct orifice cannot be identified or the guidewire cannot be advanced because of the cystic duct anatomy. Furthermore, all procedures will be performed by experienced endoscopists, and total procedure time and ETGBS-specific procedure time will be recorded separately. We have clarified these points in the Methods and have retained the 60-minute threshold.

**Changes in the manuscript (Methods, Procedural details; Lines 183–188; also described in our response to Reviewer 1, Comment 4):**

“The ETGBS attempt will be discontinued if ETGBS is not successfully completed within 60 minutes after scope insertion. The attempt may be discontinued earlier if the endoscopist considers further manipulation technically difficult or unsafe. The 60-minute limit refers to the total elapsed time from scope insertion and includes standard ERCP steps performed before the ETGBS attempt, such as biliary cannulation, cholangiography, and endoscopic sphincterotomy when required. It does not represent 60 minutes of ETGBS-specific guidewire manipulation.”

**Reviewer #2: Comment 4.**
The choice of the operator between two types of 10-mm FCSEMSs (HANAROSTENT and BONASTENT) introduces an important confounding factor. Stent biomechanics (axial and radial forces) are independent risk factors for cystic duct occlusion. These stents have different braided architecture and thus different deployed biomechanical properties. The protocol should have mandated a single model of FCSEMS or a subgroup analysis based on the type of stent to properly assess the efficacy of ETGBS.

**Response:**

Thank you for this important comment. We agree that differences in the biomechanical properties of FCSEMSs, including axial and radial forces, may influence the risk of cystic duct obstruction and acute cholecystitis after FCSEMS placement.

The present study was designed as a pragmatic multicenter exploratory study, and the two prespecified 10-mm FCSEMS models are both used in routine clinical practice at the participating institutions. Mandating a single FCSEMS model would reduce the feasibility and generalizability of the study. In addition, because enrollment has already begun, restricting subsequent enrollment to a single model would introduce inconsistency between participants enrolled before and after such a change. We therefore did not restrict FCSEMS placement to a single model.

To address the reviewer’s concern, the FCSEMS model used in each procedure will be recorded and reported. We have also added an exploratory subgroup analysis in which the incidence of acute cholecystitis within 30 days after FCSEMS placement will be summarized separately according to the FCSEMS model used (HANAROSTENT or BONASTENT). Because the target sample size is 50 participants and the expected number of acute cholecystitis events is small, no formal statistical comparison or multivariable adjustment according to FCSEMS model is planned. The results will be interpreted descriptively and as hypothesis-generating. The manuscript and study protocol have been revised accordingly.

**Changes in the manuscript (Methods, Procedural details; Lines 198–199):**
“The type and caliber of the gallbladder stent placed and the FCSEMS model used will also be recorded.”

**Changes in the manuscript (Methods, Statistical analysis; Lines 333–335):**

“The incidence of acute cholecystitis within 30 days will also be summarized descriptively according to the FCSEMS model used. No formal statistical comparison between FCSEMS models is planned.”

**Reviewer #2: Comment 5.**

Furthermore, the decision to place prophylactic plastic stents, 7-Fr or 5-Fr, based on a subjective “cholangiographic assessment” by endoscopists, complicates the interpretation of the data. By Poiseuille’s law, a 5-Fr stent is exponentially more likely to undergo early biliary sludge-induced occlusion and may inadvertently trigger the acute cholecystitis the trial seeks to prevent. On the other hand, the 5-Fr stent’s smaller caliber may be easier to deliver through tortuous ducts and may introduce performance bias. If difficult cases arise, operators may decide to use the 5-Fr stent without randomized assignment or objective sizing criteria. This conflates technical success rates with clinical efficacy and may overrepresent highly occlusive stents, thus skewing the 30-day outcome data.

**Response:**

Thank you for this important comment. We agree that nonrandomized selection between the 5-Fr and 7-Fr plastic stents may introduce confounding by indication because stent selection may be influenced by cystic duct anatomy and technical feasibility. However, we do not consider that a 5-Fr gallbladder stent can be assumed to have a higher risk of early occlusion solely on the basis of Poiseuille’s law. Clinical performance of gallbladder stents may also depend on factors such as stent design, side holes, the wicking effect, cystic duct anatomy, and ease of insertion.

In a previous comparative study of endoscopic transpapillary gallbladder stenting for acute cholecystitis, the 5-Fr and 7-Fr stents showed no significant differences in clinical success, early or late adverse events, or recurrent cholecystitis (Nakahara et al., Endosc Int Open. 2025;13:a24657130). Moreover, four stent-insertion failures occurred in the 7-Fr group, whereas none occurred in the 5-Fr group, suggesting a potential advantage of the smaller-caliber stent in terms of insertability. We acknowledge that this study involved patients with acute cholecystitis and did not evaluate the exact stent designs or clinical setting used in the present study; therefore, it does not establish equivalence between the two stents in our population.

Because cystic duct anatomy and technical deliverability vary among patients, mandating a single stent or randomly assigning stent caliber could result in unsuccessful placement or procedural injury. We therefore retained the use of the two prespecified plastic stents, with selection based on cholangiographic assessment of the course and diameter of the cystic duct and technical feasibility. The type and caliber of the stent placed will be recorded.

To address possible confounding associated with nonrandomized stent selection, the incidence of acute cholecystitis will be summarized descriptively according to the type and caliber of the plastic stent among patients with technically successful ETGBS. Because stent selection is not randomized, this analysis will not be interpreted as a causal comparison between the two stents, and no formal between-stent statistical comparison is planned given the limited sample size and expected small number of events. Furthermore, the primary endpoint is evaluated among all patients in whom ETGBS is attempted, irrespective of technical success, whereas technical success is evaluated separately as a secondary endpoint.

**Changes in the manuscript (Methods, Statistical analysis; Lines 346–349):**
“The incidence will also be summarized descriptively according to the type and caliber of the gallbladder stent placed. Because gallbladder stent selection is not randomized, these descriptive summaries will not be interpreted as causal comparisons between the two stent types.”

**Reviewer #3: Comment 1.**

Reframing the study objectives: The text repeatedly refers to evaluating “efficacy,” but as a single-arm study without a control group, the design is better suited for estimating feasibility, safety, technical success, and event rates to inform a future comparative trial. We suggest revising the objectives, endpoints, and conclusions accordingly so that the interpretation better matches the study design.

**Response:**

Thank you for this important comment. We agree that a single-arm study without a concurrent control group cannot establish the efficacy of prophylactic ETGBS. We have therefore reframed the study as an exploratory evaluation of the potential clinical benefit of attempting prophylactic ETGBS by estimating the acute cholecystitis event rate and characterizing technical feasibility and safety.

We revised the objectives and concluding statements throughout the manuscript to remove claims of confirmatory efficacy and to emphasize potential clinical benefit, event-rate estimation, technical feasibility, safety, and the role of the findings in planning future comparative studies.

We did not change the prespecified endpoints because they already comprise acute cholecystitis, technical success, and procedure-related adverse events, which are appropriate outcomes for this exploratory single-arm study. The historical comparison remains supplementary and exploratory.

**Changes in the manuscript (Abstract, Background; Lines 35–37):**

“This study aims to explore the potential clinical benefit of attempting prophylactic ETGBS by estimating the incidence of acute cholecystitis after FCSEMS placement and characterizing the technical feasibility and safety of the strategy in patients with MDBO.”

**Changes in the manuscript (Abstract, Discussion; Lines 51–52):**

“This study will provide prospective data on the potential clinical utility, technical feasibility, and safety of attempting prophylactic ETGBS and may inform the design of future comparative studies.”

**Changes in the manuscript (Introduction; Lines 81–84):**

“The aim of this study is to explore the potential clinical benefit of attempting prophylactic ETGBS by estimating the incidence of acute cholecystitis after FCSEMS placement and characterizing the technical feasibility and safety of the strategy in patients with MDBO, thereby providing preliminary data to inform the design of future comparative studies.”

**Changes in the manuscript (Discussion; Lines 448–452 and 470–483):**

“Given these uncertainties, we designed a multicenter, prospective, single-arm exploratory study to explore the potential clinical benefit of attempting prophylactic ETGBS by estimating the incidence of acute cholecystitis after FCSEMS placement and characterizing the technical feasibility and safety of the strategy in patients with MDBO. This approach will capture outcomes among all patients in whom ETGBS is attempted, rather than limiting the evaluation to technically successful cases alone.”

“To the best of our knowledge, this is the first multicenter prospective study to explore the potential clinical benefit of attempting prophylactic ETGBS during FCSEMS placement for MDBO and to characterize the technical feasibility and safety of this strategy. The decision to proceed to a future randomized trial will be based on the totality of the findings, including technical feasibility, procedure-related safety, and the observed 30-day incidence of acute cholecystitis. As a quantitative framework for interpreting the results, an upper limit of the two-sided 90% confidence interval below the historical benchmark of 12.1% will be considered supportive of proceeding to a future randomized trial, whereas achievement of the same criterion using the two-sided 95% confidence interval will be regarded as stronger supportive evidence. These criteria will be interpreted together with technical feasibility and procedure-related safety and will not, by themselves, be considered confirmatory evidence of efficacy. Conversely, low technical success, safety concerns, or an incidence that does not suggest potential benefit would indicate the need to refine the intervention or study design before proceeding to a randomized trial. The findings may therefore help clarify the potential role of this strategy in clinical practice and determine whether and how it should be evaluated in a future randomized trial.”

**Changes in the manuscript (Discussion, Limitations; Lines 453–455):**

“First, because this is a single-arm study without randomization or a concurrent control group, it is designed to estimate feasibility, safety, and event rates rather than to establish the efficacy of prophylactic ETGBS.”

**Reviewer #3: Comment 2.**

Clarifying the historical comparison: The one-sample test against a 12.1% historical rate is not fully justified, given potential differences in patient populations, stents, and the subjective exclusion criterion based on imaging judgment of cystic-duct obstruction. We suggest focusing primarily on descriptive estimation with confidence intervals and, if retained, clearly presenting the historical comparison as exploratory and non-causal.

**Response:**

Thank you for this important comment. We agree that differences in patient populations, stent selection, procedural techniques, and imaging-based patient selection limit the comparability of the present study with the historical cohort.

As also addressed in our response to Reviewer 1, Comment 9, the primary analysis focuses on descriptive estimation using a proportion and its two-sided 90% Wilson score confidence interval. The one-sample test against 12.1% is retained only as a supplementary exploratory comparison intended to identify a preliminary signal of potential benefit and inform future comparative studies. Because of the single-arm design, it cannot establish a causal treatment effect or provide confirmatory evidence of efficacy.

Relevant differences in baseline risk factors will be considered when interpreting the historical comparison where corresponding historical information is available. Nevertheless, residual confounding cannot be eliminated, particularly because of differences in patient characteristics, stents, procedural techniques, and imaging-based eligibility assessment. These limitations and the hypothesis-generating nature of the historical comparison have been explicitly acknowledged in the revised manuscript. The imaging-based exclusion criterion and its limitations have also been clarified, as described in our responses to the preceding comments.

No changes were made to the prespecified endpoints because the primary endpoint is already analyzed primarily as a descriptive estimate with a confidence interval.

**Changes in the manuscript (Methods, Statistical analysis; Lines 314–317, 323–328):**

“In the primary analysis, the frequency and proportion of participants who develop definite acute cholecystitis within 30 days after FCSEMS placement will be calculated among participants in the FAS whose primary endpoint status is ascertainable. A two-sided 90% confidence interval will be calculated using the Wilson score method.”

“As a supplementary exploratory analysis, a one-sample test for a proportion will be performed using a null value of 12.1% and a single two-sided significance level of 10%. This analysis is intended to assess whether the observed incidence provides a preliminary signal relative to the historical benchmark and to inform the design of future comparative studies. Because of the single-arm design, the comparison will not by itself be interpreted as establishing a causal treatment effect or providing confirmatory evidence of efficacy.”

**Changes in the manuscript (Discussion, Limitations; Lines 455–460):**

“Any comparison with a non-ETGBS strategy will rely on historical data and may be confounded by differences in patient characteristics, stent selection, and procedural techniques. Although relevant baseline risk factors will be described and considered when interpreting the historical comparison, residual confounding cannot be excluded. Therefore, the exploratory one-sample test will be interpreted as a hypothesis-generating comparison with the historical benchmark rather than as confirmatory evidence of treatment efficacy.”

**Reviewer #3: Comment 3.**

Defining the analysis population more clearly: The primary endpoint refers to patients in whom ETGBS is “attempted,” yet the full analysis set excludes those without a post-procedure assessment. A modified intention-to-treat or all-attempted-procedure population may better reflect the clinical strategy, with per-protocol analyses reserved for sensitivity analyses. It would also be helpful to prespecify how deaths, early surgery, or gallbladder drainage before day 30 will be handled, as these are competing events rather than simple missing data.

**Response:**

Thank you for this important comment. We agree that the primary analysis population should reflect the clinical strategy of attempting prophylactic ETGBS, irrespective of technical success. As partly clarified in response to the preceding reviewer comments, technically unsuccessful ETGBS cases will remain eligible for the primary endpoint analysis and will not be treated as attrition.

We have further clarified that the FAS is the primary analysis population and includes all enrolled participants in whom an ETGBS attempt is initiated and for whom at least one item of post-procedure outcome information is available, regardless of technical success or subsequent treatment. Participants with no post-procedure outcome information will be excluded because the primary endpoint cannot be assessed; their number and reasons for exclusion will be reported. The PPS analysis will be retained as a supplementary analysis.

We have also prespecified the handling of competing events before day 30. Participants who die without preceding acute cholecystitis will remain in the denominator and will not be counted as having the primary endpoint; deaths and their causes will be reported separately. Similarly, surgery involving cholecystectomy without preceding acute cholecystitis will be treated as a competing event. Gallbladder drainage performed for definite acute cholecystitis according to TG18 will be counted as a primary endpoint event, whereas gallbladder drainage performed for another indication in the absence of definite acute cholecystitis will be treated as a competing intervention. The timing and indications for these competing events and interventions will be reported separately.

**Changes in the manuscript (Methods, Primary endpoint; Lines 219–221):**

“Participants who die without preceding definite acute cholecystitis or who undergo cholecystectomy or gallbladder drainage for an indication other than definite acute cholecystitis within 30 days will remain in the denominator and will not be counted as having the primary endpoint.”

**Changes in the manuscript (Methods, Statistical analysis; Lines 285–291 and 318–320):**

“The full analysis set (FAS) will serve as the primary analysis population and will include all enrolled participants in whom an ETGBS attempt is initiated and for whom at least one item of post-procedure outcome information is available, regardless of the technical success of ETGBS or subsequent treatment. Participants in whom an ETGBS attempt is not initiated, participants for whom no post-procedure outcome information is available, participants without baseline data, and participants with protocol violations that preclude inclusion in the FAS will be excluded. The number of excluded participants and the reasons for exclusion will be reported.”

“Participants who are lost to follow-up before day 30 and whose primary endpoint status cannot be ascertained will be treated as having missing primary endpoint data and will be excluded from the denominator used to calculate the primary endpoint proportion. Missing data will not be imputed.”

**Reviewer #3: Comment 4.**

Standardizing the subjective eligibility criterion: Exclusion criterion 3 relies on the investigator’s judgment that FCSEMS is “unlikely” to obstruct the cystic duct. Providing objective imaging criteria and documenting reasons for exclusion would enhance reproducibility and reduce between-center variability.

**Response:**

Thank you for this important comment. We agree that standardized eligibility assessment is important for improving reproducibility and reducing between-center variability. As described in our responses to the preceding reviewer comments, we have already revised exclusion criterion 3 to remove the original open-ended judgment that FCSEMS placement was “unlikely” to obstruct the cystic duct orifice.

Under the revised criterion, exclusion is permitted only when both of the following conditions are met on pre-ERCP CT and/or MRI: (i) the cystic duct orifice is clearly identifiable, and (ii) the proximal end of the planned FCSEMS is expected to remain distal to the cystic duct orifice without overlapping it. If either condition is indeterminate, the patient will not be excluded on the basis of this criterion. The assessment will be performed independently by two experienced endoscopists at each participating institution, and exclusion will require agreement between both assessors. Thus, exclusion under criterion 3 is limited to fulfillment of these two prespecified conditions rather than an open-ended investigator judgment.

No validated quantitative cutoff or other objective imaging parameter has been established for this clinical decision, and the relevant anatomical landmarks cannot always be reliably identified on pre-ERCP imaging. We therefore did not introduce an arbitrary quantitative cutoff.

Regarding documentation of individual reasons for exclusion, the original protocol did not prespecify research data collection for patients who were screened but not enrolled. Because participant recruitment is already ongoing, introducing a new standardized screening log retrospectively across the four institutions could result in incomplete and inconsistent documentation. We therefore did not add individual-level data collection for non-enrolled patients. The limitations of site-based assessment, including the absence of blinded central imaging review and formal assessment of interobserver agreement, have already been acknowledged in the Discussion.

The relevant revisions to the Methods and Discussion are already described in our responses to the preceding reviewer comments. No additional changes were made in response to this comment.

**Changes in the manuscript:**
The relevant revisions to the Methods and Discussion are already described in our responses to the preceding reviewer comments. No additional changes were made in response to this comment.

**Reviewer #3: Comment 5.**

Approach to the late cholecystitis endpoint: Since follow-up durations will vary considerably, we recommend using time-to-event methods, such as cumulative incidence with competing risks, rather than a simple proportion for the secondary endpoint from day 31 onward.

**Response:**

Thank you for this important comment. We agree that a simple proportion does not adequately account for the considerable variation in follow-up duration or for death as a competing event in this population with advanced malignancy.

We have therefore revised the late endpoint analysis. A landmark time-to-event analysis will be performed among participants who are alive, under follow-up, and have experienced neither definite acute cholecystitis nor a competing event before day 31. The cumulative incidence function will be estimated using the Aalen–Johansen method, with death and cholecystectomy treated as competing events. Gallbladder drainage for definite acute cholecystitis will be counted as an event; drainage for another indication will be treated as a competing intervention.

Given the limited sample size and expected small number of late acute cholecystitis events, the analysis will focus on descriptive estimation of the cumulative incidence function. The cumulative incidence curve and the median observed follow-up duration from the day-31 landmark to the end of observation will be reported.

**Changes in the manuscript (Methods, Secondary endpoints; Lines 231–232):**

“the cumulative incidence of definite acute cholecystitis from day 31 after FCSEMS placement until the end of follow-up, accounting for competing events.”

**Changes in the manuscript (Methods, Statistical analysis; Lines 350–361):**

“For definite acute cholecystitis occurring from day 31 onward, a landmark time-to-event analysis will be performed among participants who are alive and under follow-up at day 31 and have experienced neither definite acute cholecystitis nor a competing event before that time. Time will be measured from the day-31 landmark to the first definite acute cholecystitis event. Death before definite acute cholecystitis and cholecystectomy or gallbladder drainage performed for an indication other than definite acute cholecystitis will be treated as competing events. Cholecystectomy or gallbladder drainage performed for definite acute cholecystitis will be counted as an acute cholecystitis event. Participants without an event or competing event will be censored at the last follow-up assessment or the administrative end of follow-up on April 30, 2028, whichever occurs first. The cumulative incidence function will be estimated using the Aalen–Johansen method. The cumulative incidence curve and the median observed follow-up duration from the day-31 landmark to the end of observation will be reported.”

**Reviewer #3: Comment 6.**

Safety oversight and protocol amendments: We noted that protocol version 1.2 was approved after enrollment began. A brief summary of substantive amendments and confirmation that the registry was updated would be helpful. Adding predefined stopping rules or a safety monitoring plan would also strengthen the study governance.

**Response:**

Thank you for this important comment. The initial protocol version 1.0 was approved on September 4, 2025, before the first participant was enrolled on October 15, 2025. Protocol versions 1.1 and 1.2 involved only organizational changes to the participating institutions and site principal investigators and did not alter the study design or analysis plan.

Protocol version 1.3 incorporated clarifications to eligibility assessment, procedural definitions, data collection, safety monitoring, and statistical analysis without changing the study intervention or primary endpoint. Protocol version 1.3 was approved on September 3, 2026, and the jRCT record was updated to reflect the current approved study information. A brief summary of the amendments and the process for communicating future substantive amendments have been added to the manuscript.

Regarding safety oversight, no fixed numerical stopping boundary has been prespecified because there is no validated safety threshold for this exploratory procedure and the limited sample size would make an arbitrary numerical boundary difficult to interpret. Instead, all adverse events will be recorded and reviewed through central monitoring. Any serious adverse event considered related or possibly related to ETGBS, or any concerning pattern of procedural adverse events, will trigger an ad hoc safety review by the study representative in consultation with the participating investigators and the Ethics Committee. Enrollment may be suspended, the protocol modified, or the study terminated if continuation is considered unsafe. We consider this risk-based safety monitoring plan more appropriate than introducing an unvalidated numerical stopping threshold.

**Changes in the manuscript (Methods, Data management and monitoring; Lines 375–380):**

“Any serious adverse event related or possibly related to ETGBS, or any concerning pattern of procedural adverse events, including cystic duct perforation, will be promptly reported and trigger an ad hoc safety review by the study representative in consultation with the participating investigators and the Ethics Committee. Enrollment may be suspended, the protocol modified, or the study terminated if continuation is considered unsafe.”

**Changes in the manuscript (Methods, Ethics and dissemination; Lines 388–398):**

“Protocol versions 1.1 and 1.2 involved only organizational changes to the participating institutions and site principal investigators and did not alter the study design or analysis plan. The current protocol version 1.3, approved on September 3, 2026, incorporated revisions to eligibility assessment, procedural definitions, data collection, safety monitoring, and statistical analysis without changing the study intervention or primary endpoint. The study was registered with the Japan Registry of Clinical Trials (jRCT1030250361) on September 12, 2025. The registry record has been updated to reflect the current approved study information. The study overview and recruitment status are publicly available in the registry. Any future substantive protocol amendments will be submitted to the Ethics Committee for approval before implementation, communicated to all participating institutions, and reflected in the trial registry as applicable.”

**Reviewer #4: Major Comment 1.**

Safety monitoring plan. Cystic duct perforation occurred in ~6% of patients in prior studies (lines 80–81, 316–317). The protocol currently describes only site-level adverse event reporting (lines 256–258) with no independent safety oversight or stopping rules. It may be worth considering a DSMB, or at minimum a pre-specified adverse event threshold for pausing enrollment.

**Response:**

Thank you for this important comment. We agree that cystic duct perforation is an important procedural adverse event requiring careful safety oversight. This concern is closely related to the preceding comment regarding safety monitoring and has been addressed through the risk-based safety monitoring plan added to the revised manuscript.

We considered establishing a separate DSMB or a fixed numerical threshold for pausing enrollment. However, this is a small, single-arm exploratory study with a target sample size of 50 participants, and no validated numerical safety threshold has been established for cystic duct perforation during ETGBS. Given the limited sample size, a threshold derived from the approximately 6% incidence reported in previous studies would be statistically unstable and potentially arbitrary. We therefore did not establish a separate DSMB or a fixed numerical stopping boundary.

Instead, all adverse events, including cystic duct perforation, will be recorded and reviewed through central monitoring by the Data Center at Yokohama City University Hospital. Any serious adverse event considered related or possibly related to ETGBS, or any concerning pattern of procedural adverse events, including cystic duct perforation, will trigger an ad hoc safety review by the study representative in consultation with the participating investigators and the Ethics Committee. Enrollment may be temporarily suspended while the safety review is conducted, and the protocol may be modified or the study terminated if continuation is considered unsafe. This approach provides a prespecified mechanism for safety review and possible enrollment suspension without relying on an unvalidated numerical threshold.

**Changes in the manuscript (Methods, Data management and monitoring; Lines 375–380):**

“Any serious adverse event related or possibly related to ETGBS, or any concerning pattern of procedural adverse events, including cystic duct perforation, will be promptly reported and trigger an ad hoc safety review by the study representative in consultation with the participating investigators and the Ethics Committee. Enrollment may be suspended, the protocol modified, or the study terminated if continuation is considered unsafe.”

**Reviewer #4: Major Comment 2**

Confidence level and significance threshold. Using 90% CIs (instead of the standard 95%) and testing at both 5% and 10% alpha without designating which is primary (lines 241–243) could create ambiguity. The authors might consider using 95% CIs and selecting a single alpha level, or providing a clear justification for the current approach.

**Response:**

Thank you for this important comment. We agree that specifying both 5% and 10% significance levels could create ambiguity regarding the interpretation of the supplementary one-sample test.

We retained the two-sided 90% confidence interval as the primary interval estimate because this is a small exploratory study intended to estimate event rates and identify a preliminary signal for planning a future comparative study. To provide a conventional estimate of precision, we also added a two-sided 95% Wilson score confidence interval as a supplementary analysis.

To eliminate ambiguity, we removed the 5% significance threshold and specified a single two-sided significance level of 10% for the supplementary exploratory one-sample test. The historical comparison will be interpreted as hypothesis-generating and will not, by itself, be considered confirmatory evidence of efficacy.

**Changes in the manuscript (Abstract, Methods; Lines 45–49):**

“The primary endpoint will be summarized as a proportion with a two-sided 90% Wilson score confidence interval. A two-sided 95% Wilson score confidence interval will also be reported as a supplementary analysis, and a supplementary exploratory one-sample test will compare the observed proportion with the historical benchmark of 12.1%.”

**Changes in the manuscript (Methods, Statistical analysis; Lines 314–317, 323–324, 332–333):**

“In the primary analysis, the frequency and proportion of participants who develop definite acute cholecystitis within 30 days after FCSEMS placement will be calculated among participants in the FAS whose primary endpoint status is ascertainable. A two-sided 90% confidence interval will be calculated using the Wilson score method.”

“As a supplementary exploratory analysis, a one-sample test for a proportion will be performed using a null value of 12.1% and a single two-sided significance level of 10%.”

“As supplementary analyses, a two-sided 95% Wilson score confidence interval will also be calculated for the primary endpoint, and the primary analysis will be repeated in the PPS.”

**Reviewer #4: Major Comment 3**
The 12.1% comparator. This number comes from one retrospective study (line 243, referencing line 222), but the cited literature reports background cholecystitis rates of 6.5–15.3% (lines 280–281). It would be helpful to explain why 12.1% was chosen over other values, and perhaps to show what the results would look like if the true rate were at the low end of that range.

**Response:**

Thank you for this important comment. We agree that the reported incidence of acute cholecystitis after covered SEMS placement varies across studies and that the historical rate of 12.1% was derived from a single retrospective study.

We selected 12.1% because the study by Ishii et al. was considered the most relevant available historical cohort for the present primary endpoint. It included 339 patients undergoing initial placement of a 10-mm covered SEMS for distal malignant biliary obstruction and excluded patients who had undergone prior or concurrent gallbladder drainage. Early acute cholecystitis was defined using the same 30-day time window as the primary endpoint of the present study and occurred in 41 of 339 patients (12.1%). In addition, this was one of the largest reported cohorts restricted to covered SEMS placement. We therefore considered this estimate more directly comparable with the present study than estimates derived from smaller or less comparable populations.

We acknowledge that the published background rates vary and that 12.1% should not be regarded as a universally applicable background incidence. We considered adding a separate comparison with the lower end of the published range. However, the studies underlying this range differ in patient populations, stent characteristics, definitions, and procedural practices, and no single alternative value can be regarded as a validated comparator. Adding multiple historical thresholds could also complicate the interpretation of this small exploratory study. We therefore retained 12.1% as the sole prespecified historical benchmark.

The comparison will remain supplementary and exploratory. An observed incidence lower than the historical benchmark will be interpreted only as a preliminary signal that prophylactic ETGBS may be beneficial and warrants evaluation in a future comparative study, rather than as confirmatory evidence of efficacy.

**Changes in the manuscript (Methods, Sample size; Lines 271–279):**

“The historical benchmark of 12.1% was selected from the study by Ishii et al. [24] because it included a relatively large cohort of 339 patients who underwent initial placement of a 10-mm covered SEMS for distal malignant biliary obstruction, excluded patients with prior or concurrent gallbladder drainage, and evaluated early acute cholecystitis using the same 30-day period as the primary endpoint of the present study. In that study, early acute cholecystitis occurred in 41 of 339 patients (12.1%). Thus, despite the limitations inherent in using a single retrospective study as a historical reference, we considered this estimate the most directly comparable benchmark available. The incidence of acute cholecystitis within 30 days after FCSEMS placement was reported to be 3.1% when ETGBS was attempted [18]. Based on these findings, we assumed an incidence of 3% in the present study.”

**Reviewer #4: Major Comment 4**

Exclusion criterion number 3 states that “Imaging suggesting FCSEMS is unlikely to obstruct the cystic duct orifice, as judged by the site principal investigator" (lines 123–125) which may be difficult for four sites to apply consistently. It could be strengthened by defining specific imaging features (e.g., distance from stricture to cystic duct orifice) that would trigger exclusion.

**Response:**

Thank you for this important comment. We agree that the original wording could have resulted in inconsistent application of exclusion criterion 3 across the participating institutions. As described in our responses to Reviewer 1, Comment 1 and Reviewer 2, Comment 1, we considered using the distance between the proximal margin of the malignant biliary stricture and the cystic duct orifice as an objective anatomical parameter. However, no validated cutoff has been established, and this distance cannot always be measured reliably on pre-ERCP imaging.

We therefore revised exclusion criterion 3 to specify that patients will be excluded only when both of the following conditions are met on pre-ERCP CT and/or MRI: (i) the cystic duct orifice is clearly identifiable, and (ii) the proximal end of the planned FCSEMS is expected to remain distal to the cystic duct orifice without overlapping it. If either condition cannot be determined, the patient will not be excluded on the basis of this criterion. The assessment will be performed independently by two experienced endoscopists at each participating institution, and exclusion will require agreement between both assessors. Thus, discordant or indeterminate assessments will not result in exclusion. We believe that these revisions improve the consistency of eligibility assessment while avoiding the use of an arbitrary, unvalidated distance cutoff.

The relevant revisions to the Methods and Discussion are already described in our responses to Reviewer 1, Comment 1 and Reviewer 2, Comment 1. No additional changes were made in response to this comment.

**Reviewer #4: Major Comment 5.**

Analysis population. The primary endpoint covers "all patients in whom ETGBS is attempted" (lines 179–180), but the FAS requires having received "the protocol-defined intervention" and at least one post-procedure assessment (lines 229–231). It would be helpful to clarify what happens to a patient whose bile duct cannulation fails before ETGBS is even tried.

**Response:**

Thank you for this comment. We have clarified the definition of an ETGBS attempt and its relationship to the analysis populations. An ETGBS attempt is defined as the initiation of intentional guidewire manipulation toward the visualized or presumed location of the cystic duct orifice. Therefore, if biliary cannulation fails before this guidewire manipulation can be initiated, the participant will not be considered to have undergone an ETGBS attempt and will not be included in the FAS or in the denominator of the primary endpoint. The participant will, however, be included in the SAS because the protocol-defined ERCP procedure has been initiated.

The number of such participants and the reasons for not initiating ETGBS, including failed biliary cannulation, will be reported. Once the protocol-defined guidewire manipulation has been initiated, the participant will be considered to have undergone an ETGBS attempt regardless of technical success and will be included in the FAS if post-procedure outcome information is available. These definitions have been clarified in the Procedural details and Statistical analysis sections.

The relevant revisions to the Procedural details and Statistical analysis sections are already described in our responses to Reviewer 1, Comment 2 and Reviewer 3, Comment 3. No additional changes were made in response to this comment.

**Reviewer #4: Minor Comment 1.**

It would be helpful to state the statistical software to be used (not currently specified in the statistical analysis section, lines 228–248).

**Response:**

Thank you for this suggestion. We have added the statistical software to be used. Because the analyses will be conducted after completion of follow-up, a software version has not been prespecified.

**Changes in the manuscript (Methods, Statistical analysis; Lines 363–365):**
“All statistical analyses will be performed using R (R Foundation for Statistical Computing, Vienna, Austria).”

**Reviewer #4: Minor Comment 2.**

The statement that "missing data will not be imputed and will be handled as missing" (line 246) could be made more informative by specifying how patients who die or are lost to follow-up before day 30 are counted in the primary endpoint.

**Response:**

Thank you for this important comment. We agree that the original statement regarding missing data did not sufficiently specify the handling of deaths and loss to follow-up before day 30.

We have clarified that participants who die within 30 days after FCSEMS placement will remain in the FAS and in the denominator of the primary endpoint. Acute cholecystitis occurring before death will be counted as a primary endpoint event, whereas death without preceding acute cholecystitis will be treated as a competing event and will not be counted as a primary endpoint event. The number and causes of deaths will be reported separately.

Participants who are lost to follow-up before day 30 without a documented primary endpoint event or a prespecified competing event will remain in the FAS, but their primary endpoint status will be treated as missing and they will be excluded from the denominator used to calculate the primary endpoint proportion. Missing primary endpoint data will not be imputed. The number of such participants, their available follow-up duration, and the reasons for loss to follow-up will be reported. Participants for whom no post-procedure outcome information is available will not be included in the FAS, and their number and reasons for exclusion will also be reported. The Statistical analysis section has been revised accordingly.

**Changes in the manuscript (Methods, Primary endpoint; Lines 219–223):**

“Participants who die without preceding definite acute cholecystitis or who undergo cholecystectomy or gallbladder drainage for an indication other than definite acute cholecystitis within 30 days will remain in the denominator and will not be counted as having the primary endpoint. The number of such participants, the timing and causes of death, and the timing and indications for these interventions will be reported separately.”

**Changes in the manuscript (Methods, Primary endpoint analysis; Lines 318–322):**

“Participants who are lost to follow-up before day 30 and whose primary endpoint status cannot be ascertained will be treated as having missing primary endpoint data and will be excluded from the denominator used to calculate the primary endpoint proportion. Missing data will not be imputed. The number of such participants, their available follow-up duration, and the reasons for loss to follow-up will be reported.”

**Reviewer #4: Minor Comment 3.**
Describing what result would justify proceeding to a randomized trial could help readers interpret the study's conclusions regardless of outcome (relevant to lines 319–322).

**Response:**

Thank you for this helpful comment. We agree that an interpretive framework for progression to a randomized trial would help readers assess the implications of the study findings. We have clarified that the decision will be based on the totality of the findings, including technical feasibility, procedure-related safety, and the observed 30-day incidence of acute cholecystitis.

As a quantitative framework, an upper limit of the two-sided 90% confidence interval below the historical benchmark of 12.1% will be considered supportive of proceeding to a future randomized trial, whereas meeting the same criterion using the two-sided 95% confidence interval will be regarded as stronger supportive evidence. These criteria will be considered together with technical feasibility and safety and will not be treated as confirmatory evidence of efficacy.

**Changes in the manuscript (Discussion, concluding paragraph; Lines 472–482):**

“The decision to proceed to a future randomized trial will be based on the totality of the findings, including technical feasibility, procedure-related safety, and the observed 30-day incidence of acute cholecystitis. As a quantitative framework for interpreting the results, an upper limit of the two-sided 90% confidence interval below the historical benchmark of 12.1% will be considered supportive of proceeding to a future randomized trial, whereas achievement of the same criterion using the two-sided 95% confidence interval will be regarded as stronger supportive evidence. These criteria will be interpreted together with technical feasibility and procedure-related safety and will not, by themselves, be considered confirmatory evidence of efficacy. Conversely, low technical success, safety concerns, or an incidence that does not suggest potential benefit would indicate the need to refine the intervention or study design before proceeding to a randomized trial.”

**Reviewer #4: Minor Comment 4.**
The 200-ERCP experience threshold (line 176) may not ensure ETGBS-specific competency. The authors could consider acknowledging this or setting a more specific requirement.

**Response:**

Thank you for this important comment. We agree that experience with at least 200 ERCP procedures reflects general ERCP experience and does not necessarily ensure ETGBS-specific competency. We considered establishing an ETGBS-specific experience requirement; however, no validated procedural-volume threshold for ETGBS competency has been established. We therefore retained the existing requirement to ensure a baseline level of ERCP experience while acknowledging that differences in ETGBS-specific experience may affect technical success and safety. This point has been added to the Limitations section.

**Changes in the manuscript (Discussion, Limitations; Lines 460–464):**

“Second, although all operators and assistants are required to have performed at least 200 ERCP procedures as the primary operator, this threshold reflects general ERCP experience and does not necessarily ensure ETGBS-specific competency. Technical success and safety may therefore depend on individual ETGBS experience, and generalizability to less experienced or lower-volume settings may be limited.”

**Reviewer #4: Minor Comment 5.**
Since three of four sites are in the Yokohama/Kanagawa area (lines 90–92), it could be worth noting this geographic concentration in the limitations section (lines 323–327).

**Response:**

Thank you for this comment. We agree that the geographic concentration of the participating institutions may limit the generalizability of the findings to other regions. All four participating institutions are located in Kanagawa Prefecture, including three in Yokohama. We have briefly acknowledged this point in the Limitations section.

**Changes in the manuscript (Discussion, Limitations; Lines 468–469):**

“Fourth, all four participating institutions are located in Kanagawa Prefecture, which may limit the geographic generalizability of the findings.”

**Reviewer #4: Minor Comment 6.**

The "80.1%" figure from Ishii et al. (line 299) seems oddly precise for a small sample, and it may be worth double-checking this value.

**Response:**

Thank you for pointing this out. We rechecked the original report by Ishii et al. The study included 339 patients, of whom 51 developed acute cholecystitis after CSEMS placement. Among these 51 patients, 41 developed acute cholecystitis within 30 days, corresponding to 80.4%. The value of 80.1% in the original manuscript was a transcription error. We have corrected the percentage to 80.4%, added the numerator and denominator for clarity, and corrected the corresponding reference.

**Changes in the manuscript (Discussion; Lines 428–430):**

“Ishii et al. reported that 41 of 51 acute cholecystitis cases (80.4%) occurred within 30 days after FCSEMS placement [24], and Kozakai et al. reported a median time to onset of 18 days after FCSEMS placement [19].”
