# Supplementary material for: Prophylactic endoscopic transpapillary gallbladder stenting at fully covered self-expandable metal stent placement to prevent cholecystitis in malignant distal biliary obstruction: A multicenter prospective single-arm study protocol (PROTECT study)
Source: PLoS One. 2026 Oct 29;21(10):e0361468. doi: 10.1371/journal.pone.0361468 (PMC13718957; doi:10.1371/journal.pone.0361468)
Supplement: Supplementary file 5 [file pone.0361468.s006.docx]

**Response to the Editor and Reviewers**

**Manuscript title:**

Prophylactic endoscopic transpapillary gallbladder stenting at fully covered self-expandable metal stent placement to prevent cholecystitis in malignant distal biliary obstruction: A multicenter prospective single-arm study protocol (PROTECT study)

**Dear Editor and Reviewers,**

Thank you for your careful and constructive review of our manuscript. We have revised the manuscript in accordance with the comments and believe that the revisions have improved its clarity and scientific rigor. The reviewers’ comments are reproduced below, followed by our responses and the corresponding changes in the revised manuscript. Page and line numbers refer to the revised manuscript.

We sincerely appreciate the time and effort devoted to evaluating our work.

**Additional Editor Comments:**

Thank you for your careful revision. The manuscript has improved considerably, and it is close to acceptance. Reviewer 5, a statistical reviewer, has raised several points, some of which are already covered in the current version (for example, the handling of deaths before day 30 and the rationale for the 90% confidence interval). Please address the remaining points before the protocol is published. In particular:

1) Give the rationale for excluding participants lost to follow-up before day 30 from the denominator, and add a simple sensitivity analysis (for example, best-case and worst-case scenarios) to show how missing primary endpoint data could affect the estimate. Please also mention this in the limitations.

2) Name the exact method for the one-sample comparison with the 12.1% benchmark (for example, exact binomial or score test).
describe how center-level variability will be assessed or considered.

3) Name the electronic data capture platform.

4) Define what counts as a major protocol deviation.

5) State whether the subgroup summaries by FCSEMS model and stent type will include confidence intervals.

6) State whether follow-up time will be summarized beyond the median.

**Response:**
Thank you for your encouraging assessment and helpful guidance. We have addressed all six points in the revised manuscript. Detailed explanations and the corresponding manuscript changes are provided below in our responses to Reviewer #5, Comments 1–3 and 6–11.

**Reviewer #5: Comment 1.**

Clarify the rationale for excluding participants lost to follow-up before day 30 from the denominator of the primary endpoint analysis and discuss the potential impact on the estimated event rate.

**Response:**

Thank you for this comment. We have clarified that participants with unascertainable primary endpoint status are excluded from the denominator of the primary analysis to avoid implicitly treating unknown outcomes as non-events. We have also added a statement to the Discussion acknowledging that this approach may introduce selection bias and reduce precision, and that the estimated event proportion may be underestimated or overestimated if the risk of acute cholecystitis differs between participants with and without ascertainable outcomes.

**Changes in the manuscript (Methods, Primary endpoint analysis; Lines 331–333):**

This approach avoids assuming that participants with incomplete follow-up and unascertainable endpoint status remained free of acute cholecystitis throughout the 30-day period.

**Changes in the manuscript (Discussion; Lines 501–504):**

Fifth, excluding participants with unascertainable primary endpoint status may introduce selection bias and reduce precision. If their risk of acute cholecystitis differs from that of participants with ascertainable outcomes, the estimated event proportion may be underestimated or overestimated.

**Reviewer #5: Comment 2.**

Conduct sensitivity analyses for missing primary endpoint data to evaluate the robustness of the primary findings under alternative assumptions.

**Response:**

Thank you for this comment. We have added planned supplementary sensitivity analyses for missing primary endpoint data. In the primary analysis, FAS participants whose primary endpoint status is unascertainable will be excluded from the denominator. In the best-case sensitivity analysis, these participants will be included in the denominator and classified as non-events, whereas in the worst-case sensitivity analysis, they will be included in the denominator and classified as events. Participants classified as non-events under the prespecified rules for death without preceding definite acute cholecystitis or for cholecystectomy or gallbladder drainage performed for other indications will retain this classification in both sensitivity analyses. The resulting event proportions and corresponding two-sided 90% Wilson score confidence intervals will be compared descriptively with the primary analysis to assess the robustness of the findings.

**Changes in the manuscript (Methods, Primary endpoint analysis; Lines 333–343):**

As supplementary sensitivity analyses, the robustness of the primary endpoint estimate to missing outcome data will be assessed using best-case and worst-case scenarios. FAS participants with unascertainable primary endpoint status will be included in the denominator and classified as non-events in the best-case scenario and as events in the worst-case scenario. Participants classified as non-events under the prespecified rules for death without preceding definite acute cholecystitis or for cholecystectomy or gallbladder drainage performed for other indications will retain this classification in both scenarios. Event proportions and corresponding two-sided 90% Wilson score confidence intervals will be calculated under each scenario and compared descriptively with the primary analysis. Missing primary endpoint data will not be imputed in the primary analysis. The number of participants with unascertainable primary endpoint status, their available follow-up duration, and the reasons for loss to follow-up will be reported.

**Reviewer #5: Comment 3.**

Specify the exact statistical method that will be used for the exploratory one-sample comparison with the historical 12.1% benchmark.

**Response:**

Thank you for this comment. We have specified the statistical method for the exploratory one-sample comparison with the historical benchmark of 12.1%. Specifically, a two-sided one-sample score test for a proportion without continuity correction will be performed using a null proportion of 12.1% and a significance level of 10%. This method was selected for consistency with the two-sided 90% Wilson score confidence interval. Because this comparison is exploratory, interpretation will emphasize the estimated event proportion and its confidence interval, with the p-value considered supplementary, hypothesis-generating information. The corresponding descriptions in the Abstract, Statistical analysis, and Discussion sections have been revised accordingly.

**Changes in the manuscript (Abstract, Methods; Lines 46–49):**

A two-sided 95% Wilson score confidence interval will also be reported as a supplementary analysis, and a supplementary exploratory two-sided one-sample score test will compare the observed proportion with the historical benchmark of 12.1%.

**Changes in the manuscript (Methods, Statistical analysis; Lines 344–346):**

As a supplementary exploratory analysis, a two-sided one-sample score test for a proportion without continuity correction will be performed using a null proportion of 12.1% and a significance level of 10%.

**Changes in the manuscript (Discussion; Lines 489–492):**

Therefore, the exploratory one-sample score test without continuity correction will be interpreted as a hypothesis-generating comparison with the historical benchmark rather than as confirmatory evidence of treatment efficacy.

**Reviewer #5: Comment 4.**

Clarify how deaths occurring before day 30 without acute cholecystitis will be interpreted in relation to the primary endpoint and discuss the potential implications of treating such events differently from missing outcomes.

**Response:**

Thank you for this comment. As already specified in the manuscript, participants who die within 30 days without preceding definite acute cholecystitis will remain in the denominator and will not be counted as having experienced the primary endpoint. Such deaths are distinguished from missing outcomes because the absence of acute cholecystitis can be ascertained up to the time of death, whereas participants with unascertainable primary endpoint status have insufficient information to determine the outcome. The number of such participants and the timing and causes of death will be reported separately.

**Reviewer #5: Comment 5.**

Provide additional justification for the use of a two-sided 90% confidence interval as the primary measure of precision rather than the more conventional 95% confidence interval.

**Response:**

Thank you for this comment. The two-sided 90% Wilson score confidence interval was prespecified as the primary interval estimate for this exploratory single-arm study, which aims to estimate the event rate and provide preliminary information for planning a future comparative study. Its use reflects the exploratory purpose of the study and is not intended to provide confirmatory evidence of efficacy. We acknowledge that a 90% confidence interval provides a lower level of confidence than a 95% interval. Accordingly, as already specified in the manuscript, a two-sided 95% Wilson score confidence interval will also be reported as a supplementary analysis to allow assessment at the conventional confidence level.

**Reviewer #5: Comment 6.**

Describe how center-level variability across the four participating institutions will be assessed or accounted for in the interpretation of study results.

**Response:**

Thank you for this comment. We have added a description of how center-level variability will be explored in the “Descriptive analyses” subsection. Enrollment numbers, key baseline characteristics, and primary endpoint event counts, denominators, and proportions will be summarized separately for the four participating institutions. Given the limited sample size and expected small number of events, formal between-center comparisons and center-adjusted regression analyses are not planned. Differences across institutions will be considered when interpreting the pooled results, with appropriate caution regarding the limited precision of center-specific estimates.

**Changes in the manuscript (Methods, Descriptive analysis; Lines 316–322):**

To explore center-level variability, the number of enrolled participants, key baseline characteristics, and primary endpoint event counts, denominators, and proportions will be summarized by institution. The same denominator criteria as in the overall primary analysis will be applied within each institution. Given the limited sample size and expected small number of events, no formal between-center comparisons or center-adjusted regression analyses are planned. Differences in recruitment, patient characteristics, and observed event rates across institutions will be considered when interpreting the pooled results, recognizing the limited precision of center-specific estimates.

**Reviewer #5: Comment 7.**

Specify the software or platform that will be used for electronic data capture and data collection.

**Response:**

Thank you for this comment. We have clarified the data collection and data management procedures in the Methods section. No dedicated electronic data capture system will be used. Investigators at each participating institution will initially record data on paper case report forms, and de-identified data will subsequently be entered centrally into a password-protected Microsoft Excel database by authorized study-office personnel. Electronic entries will be verified against the paper case report forms by a designated data-management investigator, discrepancies will be resolved through queries to the relevant institution, and subsequent changes will be documented in a data-change log with version-controlled copies retained. Access to the electronic database will be restricted to authorized study personnel, and the database will be locked before statistical analysis.

**Changes in the manuscript (Methods, Data management and monitoring; Lines 392–403):**

Data will initially be recorded on paper case report forms by investigators at each participating institution. Completed case report forms, identified only by study-specific participant codes, will be submitted to the study office. No dedicated electronic data capture system will be used. Authorized study-office personnel will centrally enter the data into a password-protected Microsoft Excel database (Microsoft Corporation, Redmond, WA, USA). A designated data-management investigator will verify the electronic entries against the paper case report forms, and any discrepancies will be resolved through queries to the relevant institution. Corrections to the electronic database will be documented in a data-change log, and version-controlled copies will be retained. The database will be locked before statistical analysis. Identifiable participant information and de-identified study data will be stored separately, and access will be restricted to authorized study personnel. Electronic data will be stored on password-protected devices or electronic media, and paper records will be stored securely in locked cabinets.

**Reviewer #5: Comment 8.**

Report the exact criteria that will define a major protocol deviation for inclusion in the per-protocol analysis set.

**Response:**

Thank you for this comment. We have clarified the definition of major protocol deviations for PPS eligibility in the “Analysis populations” subsection. These deviations are defined as confirmed failure to meet any prespecified inclusion criterion or fulfillment of any prespecified exclusion criterion at enrollment, including violations identified after enrollment. We have also clarified that the expected survival criterion is based on the site principal investigator’s assessment at enrollment and that death within 4 months after enrollment does not, in itself, constitute a violation of this criterion. Technical failure of ETGBS alone is likewise not considered a major protocol deviation. The number of participants excluded from the PPS and the specific eligibility criteria violated will be reported. If the FAS and PPS are identical, this will be explicitly stated. These clarifications do not change the prespecified inclusion or exclusion criteria.

**Changes in the manuscript (Methods, Statistical analysis; Lines 292–300):**

The per-protocol set (PPS) will be defined as a subset of the FAS, excluding participants with major protocol deviations related to eligibility. For PPS eligibility, such deviations will be defined as confirmed failure to meet any of the prespecified inclusion criteria (criteria 1–5) or fulfillment of any of the prespecified exclusion criteria (criteria 1–9) at enrollment, including violations identified after enrollment. For the expected survival criterion, eligibility will be based on the site principal investigator’s assessment at enrollment. Death occurring within 4 months after enrollment will not, in itself, be considered a violation of this criterion. Technical failure of ETGBS will not, in itself, constitute a major protocol deviation. The number of participants excluded from the PPS and the specific eligibility criteria violated will be reported.

**Changes in the manuscript (Methods, Statistical analysis; Lines 307–308):**

The PPS analysis will be supplementary. If the FAS and PPS are identical, this will be explicitly reported.

**Reviewer #5: Comment 9.**

Describe whether exploratory subgroup summaries according to FCSEMS model or gallbladder stent type will include corresponding confidence intervals.

**Response:**

Thank you for this comment. We have clarified in the “Primary endpoint analysis” and “Secondary endpoint analyses” subsections that exploratory subgroup summaries by FCSEMS model and gallbladder stent type and caliber will include event counts, denominators, and proportions with corresponding two-sided 90% Wilson score confidence intervals. This is consistent with the main interval estimation approach used for the primary and secondary endpoints. These summaries will remain exploratory and descriptive. The confidence intervals will describe the precision of subgroup-specific estimates and will not be used to draw confirmatory conclusions regarding differences between devices.

**Changes in the manuscript (Methods, Primary endpoint analysis; Lines 354–357):**

The incidence of acute cholecystitis within 30 days will also be summarized descriptively according to the FCSEMS model used, reporting event counts, denominators, and proportions with two-sided 90% Wilson score confidence intervals.

**Changes in the manuscript (Methods, Secondary endpoint analysis; Lines 368–370):**

The incidence will also be summarized descriptively according to the type and caliber of the gallbladder stent placed, reporting event counts, denominators, and proportions with two-sided 90% Wilson score confidence intervals.

**Reviewer #5: Comment 10.**

Clarify whether follow-up time distributions will be summarized beyond the planned reporting of median observed follow-up duration.

**Response:**

Thank you for this comment. We have clarified in the “Secondary endpoint analyses” subsection that observed follow-up duration from the day-31 landmark to the end of observation among participants included in the landmark analysis will be summarized using the median and interquartile range (25th–75th percentiles). Reporting the interquartile range will provide information on the variability of follow-up duration beyond the median alone.

**Changes in the manuscript (Methods, Secondary endpoint analysis; Lines 382–385):**

The cumulative incidence curve will be reported. Among participants included in the landmark analysis, observed follow-up duration from the day-31 landmark to the end of observation will be summarized using the median and interquartile range (25th–75th percentiles).

**Reviewer #5: Comment 11.**

Discuss the limitations associated with excluding participants with unascertainable primary endpoint status from the primary analysis population.

**Response:**

Thank you for this comment. As also addressed in our response to Comment 1, we have added a statement to the limitations paragraph of the Discussion acknowledging that exclusion of participants with unascertainable primary endpoint status from the primary endpoint denominator may introduce selection bias and reduce precision. We also state that the event proportion may be underestimated or overestimated if the risk of acute cholecystitis differs between participants with and without ascertainable outcomes.

**Changes in the manuscript (Discussion; Lines 501–504):**

Fifth, excluding participants with unascertainable primary endpoint status may introduce selection bias and reduce precision. If their risk of acute cholecystitis differs from that of participants with ascertainable outcomes, the estimated event proportion may be underestimated or overestimated.
