# Supplementary material for: Prophylactic endoscopic transpapillary gallbladder stenting at fully covered self-expandable metal stent placement to prevent cholecystitis in malignant distal biliary obstruction: A multicenter prospective single-arm study protocol (PROTECT study)
Source: PLoS One. 2026 Oct 29;21(10):e0361468. doi: 10.1371/journal.pone.0361468 (PMC13718957; doi:10.1371/journal.pone.0361468)
Supplement: Peer review history

# Peer review: Decision Letter 0

**DOI:** 10.1371/journal.pone.0361468.r001

**Authors:** Athanasios G. Pantelis

14 Jul 2026

Dear Dr. kurita,

Thank you for submitting your manuscript to PLOS ONE. After careful consideration, we feel that it has merit but does not fully meet PLOS ONE’s publication criteria as it currently stands. Therefore, we invite you to submit a revised version of the manuscript that addresses the points raised during the review process.

==============================

**ACADEMIC EDITOR:**  Please address all reviewers' comments before resubmitting your manuscript.

==============================

Please submit your revised manuscript by Aug 13 2026 11:59PM. If you will need more time than this to complete your revisions, please reply to this message or contact the journal office at plosone@plos.org. When you're ready to submit your revision, log on to https://www.editorialmanager.com/pone/ and select the 'Submissions Needing Revision' folder to locate your manuscript file.

- A letter that responds to each point raised by the academic editor and reviewer(s). You should upload this letter as a separate file labeled 'Response to Reviewers'.
- A marked-up copy of your manuscript that highlights changes made to the original version. You should upload this as a separate file labeled 'Revised Manuscript with Track Changes'.
- An unmarked version of your revised paper without tracked changes. You should upload this as a separate file labeled 'Manuscript'.

If you would like to make changes to your financial disclosure, please include your updated statement in your cover letter. Guidelines for resubmitting your figure files are available below the reviewer comments at the end of this letter.

If applicable, we recommend that you deposit your laboratory protocols in protocols.io to enhance the reproducibility of your results. Protocols.io assigns your protocol its own identifier (DOI) so that it can be cited independently in the future. For instructions see: https://journals.plos.org/plosone/s/submission-guidelines#loc-laboratory-protocols. Additionally, PLOS ONE offers an option for publishing peer-reviewed Lab Protocol articles, which describe protocols hosted on protocols.io. Read more information on sharing protocols at https://plos.org/protocols?utm_medium=editorial-email&utm_source=authorletters&utm_campaign=protocols.

As the corresponding author, your ORCID iD is verified in the submission system and will appear in the published article. PLOS supports the use of ORCID, and we encourage all coauthors to register for an ORCID iD and use it as well. Please encourage your coauthors to verify their ORCID iD within the submission system before final acceptance, as unverified ORCID iDs will not appear in the published article. *Only*  the individual author can complete the verification step; PLOS staff *cannot*  verify ORCID iDs on behalf of authors.

We look forward to receiving your revised manuscript.

Kind regards,

Athanasios G. Pantelis

Academic Editor

PLOS One

Journal Requirements:

When submitting your revision, we need you to address these additional requirements.

1. Please ensure that your manuscript meets PLOS ONE's style requirements, including those for file naming. The PLOS ONE style templates can be found at

https://journals.plos.org/plosone/s/file?id=wjVg/PLOSOne_formatting_sample_main_body.pdf and https://journals.plos.org/plosone/s/file?id=ba62/PLOSOne_formatting_sample_title_authors_affiliations.pdf

2. Your ethics statement should only appear in the Methods section of your manuscript. If your ethics statement is written in any section besides the Methods, please delete it from any other section.

3. Please provide a complete Data Availability Statement in the submission form, ensuring you include all necessary access information or a reason for why you are unable to make your data freely accessible. If your research concerns only data provided within your submission, please write "All data are in the manuscript and/or supporting information files" as your Data Availability Statement.

4. Thank you for providing your underlying data as Supporting Information.

We note that the data set contains text or data that is not in English. Please note that PLOS is an English-language publisher, so we require data sets to be provided in English as well. Please upload an English-language version of your data set.

This will also allow us to determine if your data follows PLOS standards per our Data Availability policy here: https://journals.plos.org/plosone/s/data-availability

5. If the reviewer comments include a recommendation to cite specific previously published works, please review and evaluate these publications to determine whether they are relevant and should be cited. There is no requirement to cite these works unless the editor has indicated otherwise.

[Note: HTML markup is below. Please do not edit.]

Reviewers' comments:

Reviewer's Responses to Questions

**Comments to the Author**

1. Does the manuscript provide a valid rationale for the proposed study, with clearly identified and justified research questions?

Reviewer #1: Yes

Reviewer #2: Yes

Reviewer #3: No

Reviewer #4: Yes

**********

2. Is the protocol technically sound and planned in a manner that will lead to a meaningful outcome and allow testing the stated hypotheses?

Reviewer #1: Yes

Reviewer #2: Partly

Reviewer #3: No

Reviewer #4: Partly

**********

3. Is the methodology feasible and described in sufficient detail to allow the work to be replicable?

Reviewer #1: Yes

Reviewer #2: Yes

Reviewer #3: No

Reviewer #4: Yes

**********

4. Have the authors described where all data underlying the findings will be made available when the study is complete?

The PLOS Data policy

Reviewer #1: Yes

Reviewer #2: Yes

Reviewer #3: Yes

Reviewer #4: Yes

**********

5. Is the manuscript presented in an intelligible fashion and written in standard English?

Reviewer #1: Yes

Reviewer #2: Yes

Reviewer #3: Yes

Reviewer #4: Yes

**********

Please use the space provided to explain your answers to the questions above and, if applicable, provide comments about issues authors must address before this protocol can be accepted for publication. You may also include additional comments for the author, including concerns about research or publication ethics.

You may also provide optional suggestions and comments to authors that they might find helpful in planning their study.

Reviewer #1: 1. Exclusion criterion 3 excludes patients if pre-ERCP imaging suggests FCSEMS placement is "unlikely to obstruct the cystic duct orifice, as judged by the site principal investigator." Because this evaluation is highly subjective, please clarify if any specific anatomical parameters (e.g., distance from the tumor margin to the cystic duct confluence) will be used to standardize this decision across the four participating institutions to minimize selection bias (pp. 12-13).

2. The primary endpoint is evaluated among all patients in whom ETGBS is "attempted" (p. 16). Please explicitly define what constitutes a valid "attempt" (e.g., does it require intentional guidewire cannulation of the cystic duct orifice, or simply the visualization of the orifice during ERCP?) (pp. 15-16). This is critical for clear baseline registration and FAS population tracking (p. 19).

3. In the Sample Size section, you anticipate a 10% attrition rate (leaving 45 evaluable patients) (p. 18). Please clarify whether patients with technically failed ETGBS procedures are considered part of this 10% attrition or if they remain fully evaluable within the FAS group, as lines 179–180 state the primary endpoint covers all attempted cases (pp. 16, 18-19).

4. Line 174 states that procedure time will be measured from scope insertion to scope withdrawal (p. 16). Since attempting ETGBS can significantly prolong ERCP procedures (p. 10), consider tracking the specific time spent solely on attempting the gallbladder stenting (up to the allowed 60 minutes) versus the time spent on the standard biliary stent placement (pp. 15-16).

5. Lines 157–160 and 165–167 allow for various brands of guidewires, catheters, and plastic stents ("or equivalent") (p. 15). Because different guidewire stiffness profiles and stent designs (5-Fr vs. 7-Fr) can impact technical success or cystic duct perforation rates (pp. 10, 15), please state whether these device specifics will be recorded and explored in post hoc analyses (p. 20).

6. While you appropriately define the primary endpoint using the Tokyo Guidelines 2018 (TG18) for definite acute cholecystitis, you do not mention severity grading (pp. 16-17). Will the study capture and report the severity (Grade I, II, or III) of the cholecystitis events? This would add high-utility data regarding clinical impact.

7. If a patient experiences a failed ETGBS attempt and subsequently develops severe acute cholecystitis (pp. 23-24), is there a standardized management protocol across the four institutions (e.g., prioritizing EUS-guided drainage vs. percutaneous cholecystostomy) (p. 10)? Please clarify if rescue therapies will be tracked as a secondary safety outcome (p. 16).

8. Secondary endpoint 4 evaluates the incidence of acute cholecystitis from day 31 until the end of follow-up (April 2028) (pp. 16-17). Given that the inclusion criteria require an expected survival of at least 4 months, please provide an estimate of the expected median follow-up duration per patient to help contextualize long-term stent patency (pp. 12, 21).

9. As noted in your limitations, this single-arm study relies on historical thresholds (12.1%) for comparison (pp. 18, 24). Please detail how you plan to address confounding baseline variations (such as the presence of pre-existing gallstones or tumor invasion of the cystic duct) when interpreting the exploratory one-sample test results (pp. 19, 21-22).

10. Line 248 mentions that no interim analyses are planned (p. 20). Since historical literature reports procedural risks like cystic duct perforation from ETGBS attempts (pp. 10, 22), please clarify if the central Data Center at Yokohama City University Hospital has specific "stopping rules" or safety thresholds that would trigger an unplanned safety review if procedural adverse events exceed acceptable baselines (p. 20).

Reviewer #2: The research question is the management of a clinically important and costly complication of malignant distal biliary obstruction (MDBO). A safe and effective prophylactic protocol for ETGBS may improve quality of life, reduce readmissions and prevent delays in oncology treatment. This represents a sound methodological step forward from single-center retrospective data to a prospective multicenter design, which enhances the generalizability of these findings.

Exclusion Criterion #3 rules out patients based on "Pre-ERCP imaging findings... suggesting that FCSEMS placement is unlikely to obstruct the cystic duct orifice, as judged by the site's principal investigator." "This introduces substantial selection bias, as it relies on an unquantified visual assessment by unblinded investigators. The protocol lacks standardized, reproducible radiological criteria (e.g., minimum tumor margin distance from the cystic duct, insertion angle, or volumetric tumor burden) to objectively define obstruction likelihood. Furthermore, the lack of an independent, blinded central radiological review to evaluate the inter-observer agreement (e.g., by Kappa or intraclass correlation coefficient) severely compromises the study’s external validity and cohort representativeness.

Inclusion criterion 4: “Expected survival of at least 4 months." Without the use of validated prognostic scoring systems, predicting survival in advanced MDBO is difficult. The protocol does not specify the quantification of this metric, ignoring existing standards such as ECOG performance status, TNM staging, or liver function scores (Child-Pugh/MELD). The use of subjective investigator discretion is likely to lead to heterogeneity among cohorts across participating centers. If institutions enroll patients with different disease aggressiveness who expire prior to the 30-day evaluation, the primary endpoint can be confounded due to survivorship bias. by survivorship bias.

The protocol allows for up to 60 minutes of ETGBS attempts before discontinuation. Such a duration poses serious safety issues and is inconsistent with the accepted interventional guidelines. ESGE (European Society of Gastrointestinal Endoscopy) defines difficult biliary cannulation as taking more than 5 minutes or requiring more than 5 papillary contacts. Since extensive papillary manipulation is the main independent risk factor for post-ERCP pancreatitis (PEP), an hour of cannulation attempts biases the risk of severe papillary trauma, massive local edema, and accidental pancreatic duct cannulation.

The choice of the operator between two types of 10-mm FCSEMSs (HANAROSTENT and BONASTENT) introduces an important confounding factor. Stent biomechanics (axial and radial forces) are independent risk factors for cystic duct occlusion. These stents have different braided architecture and thus different deployed biomechanical properties. The protocol should have mandated a single model of FCSEMS or a subgroup analysis based on the type of stent to properly assess the efficacy of ETGBS.

Furthermore, the decision to place prophylactic plastic stents, 7-Fr or 5-Fr, based on a subjective “cholangiographic assessment” by endoscopists, complicates the interpretation of the data. By Poiseuille’s law, a 5-Fr stent is exponentially more likely to undergo early biliary sludge-induced occlusion and may inadvertently trigger the acute cholecystitis the trial seeks to prevent. On the other hand, the 5-Fr stent’s smaller caliber may be easier to deliver through tortuous ducts and may introduce performance bias. If difficult cases arise, operators may decide to use the 5-Fr stent without randomized assignment or objective sizing criteria. This conflates technical success rates with clinical efficacy and may overrepresent highly occlusive stents, thus skewing the 30-day outcome data.

Reviewer #3: This manuscript presents a multicenter prospective protocol addressing an important and insufficiently studied clinical question. The multicenter prospective design, the inclusion of all attempted ETGBS procedures rather than only technically successful cases, and the use of validated definitions for cholecystitis and adverse events are important strengths that enhance the methodological rigor of the protocol.

However, several points should be addressed to improve the manuscript.

1-Reframing the study objectives: The text repeatedly refers to evaluating “efficacy,” but as a single-arm study without a control group, the design is better suited for estimating feasibility, safety, technical success, and event rates to inform a future comparative trial. We suggest revising the objectives, endpoints, and conclusions accordingly so that the interpretation better matches the study design.

2-Clarifying the historical comparison: The one-sample test against a 12.1% historical rate is not fully justified, given potential differences in patient populations, stents, and the subjective exclusion criterion based on imaging judgment of cystic-duct obstruction. We suggest focusing primarily on descriptive estimation with confidence intervals and, if retained, clearly presenting the historical comparison as exploratory and non-causal.

3-Defining the analysis population more clearly: The primary endpoint refers to patients in whom ETGBS is “attempted,” yet the full analysis set excludes those without a post-procedure assessment. A modified intention-to-treat or all-attempted-procedure population may better reflect the clinical strategy, with per-protocol analyses reserved for sensitivity analyses. It would also be helpful to prespecify how deaths, early surgery, or gallbladder drainage before day 30 will be handled, as these are competing events rather than simple missing data.

4-Standardizing the subjective eligibility criterion: Exclusion criterion 3 relies on the investigator’s judgment that FCSEMS is “unlikely” to obstruct the cystic duct. Providing objective imaging criteria and documenting reasons for exclusion would enhance reproducibility and reduce between-center variability.

5-Approach to the late cholecystitis endpoint: Since follow-up durations will vary considerably, we recommend using time-to-event methods, such as cumulative incidence with competing risks, rather than a simple proportion for the secondary endpoint from day 31 onward.

6-Safety oversight and protocol amendments: We noted that protocol version 1.2 was approved after enrollment began. A brief summary of substantive amendments and confirmation that the registry was updated would be helpful. Adding predefined stopping rules or a safety monitoring plan would also strengthen the study governance.

Reviewer #4: This is a well-designed protocol studying a practical clinical question. The choice to analyze all patients in whom ETGBS is attempted (not just successes) is the right approach. The issues below are fixable and would meaningfully strengthen the protocol.

Major Comments

1. Safety monitoring plan. Cystic duct perforation occurred in ~6% of patients in prior studies (lines 80–81, 316–317). The protocol currently describes only site-level adverse event reporting (lines 256–258) with no independent safety oversight or stopping rules. It may be worth considering a DSMB, or at minimum a pre-specified adverse event threshold for pausing enrollment.

2. Confidence level and significance threshold. Using 90% CIs (instead of the standard 95%) and testing at both 5% and 10% alpha without designating which is primary (lines 241–243) could create ambiguity. The authors might consider using 95% CIs and selecting a single alpha level, or providing a clear justification for the current approach.

3. The 12.1% comparator. This number comes from one retrospective study (line 243, referencing line 222), but the cited literature reports background cholecystitis rates of 6.5–15.3% (lines 280–281). It would be helpful to explain why 12.1% was chosen over other values, and perhaps to show what the results would look like if the true rate were at the low end of that range.

4. Exclusion criterion number 3 states that “Imaging suggesting FCSEMS is unlikely to obstruct the cystic duct orifice, as judged by the site principal investigator" (lines 123–125) which may be difficult for four sites to apply consistently. It could be strengthened by defining specific imaging features (e.g., distance from stricture to cystic duct orifice) that would trigger exclusion.

5. Analysis population. The primary endpoint covers "all patients in whom ETGBS is attempted" (lines 179–180), but the FAS requires having received "the protocol-defined intervention" and at least one post-procedure assessment (lines 229–231). It would be helpful to clarify what happens to a patient whose bile duct cannulation fails before ETGBS is even tried.

Minor Comments

1. It would be helpful to state the statistical software to be used (not currently specified in the statistical analysis section, lines 228–248).

2. The statement that "missing data will not be imputed and will be handled as missing" (line 246) could be made more informative by specifying how patients who die or are lost to follow-up before day 30 are counted in the primary endpoint.

3. Describing what result would justify proceeding to a randomized trial could help readers interpret the study's conclusions regardless of outcome (relevant to lines 319–322).

4. The 200-ERCP experience threshold (line 176) may not ensure ETGBS-specific competency. The authors could consider acknowledging this or setting a more specific requirement.

5. Since three of four sites are in the Yokohama/Kanagawa area (lines 90–92), it could be worth noting this geographic concentration in the limitations section (lines 323–327).

6. The "80.1%" figure from Ishii et al. (line 299) seems oddly precise for a small sample, and it may be worth double-checking this value.

**********

what does this mean?). If published, this will include your full peer review and any attached files.

If you choose “no”, your identity will remain anonymous but your review may still be made public.

**Do you want your identity to be public for this peer review?**  For information about this choice, including consent withdrawal, please see our Privacy Policy

Reviewer #1: **Yes:**  Hamza Islam

Reviewer #2: **Yes:**  Ali Abdi

Reviewer #3: No

Reviewer #4: No

**********

[NOTE: If reviewer comments were submitted as an attachment file, they will be attached to this email and accessible via the submission site. Please log into your account, locate the manuscript record, and check for the action link "View Attachments". If this link does not appear, there are no attachment files.]

To ensure your figures meet our technical requirements, please review our figure guidelines: https://journals.plos.org/plosone/s/figures

You may also use PLOS’s free figure tool, NAAS, to help you prepare publication quality figures: https://journals.plos.org/plosone/s/figures#loc-tools-for-figure-preparation.

NAAS will assess whether your figures meet our technical requirements by comparing each figure against our figure specifications.

# Author comment: Author response to Decision Letter 1

**DOI:** 10.1371/journal.pone.0361468.r002

13 Sep 2026

We thank the Editor and Reviewers for their careful evaluation of our manuscript. We have addressed all comments and revised the manuscript accordingly. A detailed point-by-point response has been uploaded as a separate “Response to Reviewers” file, together with clean and tracked-changes versions of the revised manuscript.

Attachment Submitted filename: Response_to_Reviewers.docx

# Peer review: Decision Letter 1

**DOI:** 10.1371/journal.pone.0361468.r003

**Authors:** Athanasios G. Pantelis

24 Sep 2026

Dear Dr. kurita,

Thank you for submitting your manuscript to PLOS One. After careful consideration, we feel that it has merit but does not fully meet PLOS One’s publication criteria as it currently stands. Therefore, we invite you to submit a revised version of the manuscript that addresses the points raised during the review process.

==============================

**ACADEMIC EDITOR:**  Please see my comments below.

Please submit your revised manuscript by Oct 09 2026 11:59PM. If you will need more time than this to complete your revisions, please reply to this message or contact the journal office at plosone@plos.org. When you're ready to submit your revision, log on to https://www.editorialmanager.com/pone/ and select the 'Submissions Needing Revision' folder to locate your manuscript file.

- A letter that responds to each point raised by the academic editor and reviewer(s). You should upload this letter as a separate file labeled 'Response to Reviewers'.
- A marked-up copy of your manuscript that highlights changes made to the original version. You should upload this as a separate file labeled 'Revised Manuscript with Track Changes'.
- An unmarked version of your revised paper without tracked changes. You should upload this as a separate file labeled 'Manuscript'.

If you would like to make changes to your financial disclosure, please include your updated statement in your cover letter. Guidelines for resubmitting your figure files are available below the reviewer comments at the end of this letter.

If applicable, we recommend that you deposit your laboratory protocols in protocols.io to enhance the reproducibility of your results. Protocols.io assigns your protocol its own identifier (DOI) so that it can be cited independently in the future. For instructions see: https://journals.plos.org/plosone/s/submission-guidelines#loc-laboratory-protocols. Additionally, PLOS One offers an option for publishing peer-reviewed Lab Protocol articles, which describe protocols hosted on protocols.io. Read more information on sharing protocols at https://plos.org/protocols?utm_medium=editorial-email&utm_source=authorletters&utm_campaign=protocols.

As the corresponding author, your ORCID iD is verified in the submission system and will appear in the published article. PLOS supports the use of ORCID, and we encourage all coauthors to register for an ORCID iD and use it as well. Please encourage your coauthors to verify their ORCID iD within the submission system before final acceptance, as unverified ORCID iDs will not appear in the published article. *Only*  the individual author can complete the verification step; PLOS staff *cannot*  verify ORCID iDs on behalf of authors.

We look forward to receiving your revised manuscript.

Kind regards,

Athanasios G. Pantelis

Academic Editor

PLOS One

**Journal Requirements:**

1. If the reviewer comments include a recommendation to cite specific previously published works, please review and evaluate these publications to determine whether they are relevant and should be cited. There is no requirement to cite these works unless the editor has indicated otherwise.

2. Please review your reference list to ensure that it is complete and correct. If you have cited papers that have been retracted, please include the rationale for doing so in the manuscript text, or remove these references and replace them with relevant current references. Any changes to the reference list should be mentioned in the rebuttal letter that accompanies your revised manuscript. If you need to cite a retracted article, indicate the article’s retracted status in the References list and also include a citation and full reference for the retraction notice.

**Additional Editor Comments:**

Thank you for your careful revision. The manuscript has improved considerably, and it is close to acceptance. Reviewer 5, a statistical reviewer, has raised several points, some of which are already covered in the current version (for example, the handling of deaths before day 30 and the rationale for the 90% confidence interval). Please address the remaining points before the protocol is published. In particular:

1) Give the rationale for excluding participants lost to follow-up before day 30 from the denominator, and add a simple sensitivity analysis (for example, best-case and worst-case scenarios) to show how missing primary endpoint data could affect the estimate. Please also mention this in the limitations.

2) Name the exact method for the one-sample comparison with the 12.1% benchmark (for example, exact binomial or score test).

describe how center-level variability will be assessed or considered.

3) Name the electronic data capture platform.

4) Define what counts as a major protocol deviation.

5) State whether the subgroup summaries by FCSEMS model and stent type will include confidence intervals.

6) State whether follow-up time will be summarized beyond the median.

These are minor, and I expect to evaluate the revised manuscript without further external review.

[Note: HTML markup is below. Please do not edit.]

Reviewers' comments:

Reviewer's Responses to Questions

**Comments to the Author**

1. Does the manuscript provide a valid rationale for the proposed study, with clearly identified and justified research questions?

Reviewer #5: Partly

**********

2. Is the protocol technically sound and planned in a manner that will lead to a meaningful outcome and allow testing the stated hypotheses?

Reviewer #5: Partly

**********

3. Is the methodology feasible and described in sufficient detail to allow the work to be replicable?

Reviewer #5: Yes

**********

4. Have the authors described where all data underlying the findings will be made available when the study is complete?

The PLOS Data policy

Reviewer #5: Yes

**********

5. Is the manuscript presented in an intelligible fashion and written in standard English?

Reviewer #5: Yes

**********

Please use the space provided to explain your answers to the questions above and, if applicable, provide comments about issues authors must address before this protocol can be accepted for publication. You may also include additional comments for the author, including concerns about research or publication ethics.

You may also provide optional suggestions and comments to authors that they might find helpful in planning their study.

Reviewer #5: This manuscript presents the protocol for the PROTECT study, a multicenter prospective single-arm exploratory study that will enroll 50 patients with malignant distal biliary obstruction undergoing fully covered self-expandable metal stent (FCSEMS) placement and attempt prophylactic endoscopic transpapillary gallbladder stenting (ETGBS) in all participants. The primary objective is to estimate the 30-day incidence of acute cholecystitis following FCSEMS placement, while secondary objectives include evaluating the technical success, safety, and longer-term outcomes of ETGBS to generate prospective data that may inform the design of future comparative studies.

Minor Statistical Issues

1- Clarify the rationale for excluding participants lost to follow-up before day 30 from the denominator of the primary endpoint analysis and discuss the potential impact on the estimated event rate.

2- Conduct sensitivity analyses for missing primary endpoint data to evaluate the robustness of the primary findings under alternative assumptions.

3- Specify the exact statistical method that will be used for the exploratory one-sample comparison with the historical 12.1% benchmark.

4- Clarify how deaths occurring before day 30 without acute cholecystitis will be interpreted in relation to the primary endpoint and discuss the potential implications of treating such events differently from missing outcomes.

5- Provide additional justification for the use of a two-sided 90% confidence interval as the primary measure of precision rather than the more conventional 95% confidence interval.

6- Describe how center-level variability across the four participating institutions will be assessed or accounted for in the interpretation of study results.

7- Specify the software or platform that will be used for electronic data capture and data collection.

8- Report the exact criteria that will define a major protocol deviation for inclusion in the per-protocol analysis set.

9- Describe whether exploratory subgroup summaries according to FCSEMS model or gallbladder stent type will include corresponding confidence intervals.

10- Clarify whether follow-up time distributions will be summarized beyond the planned reporting of median observed follow-up duration.

11- Discuss the limitations associated with excluding participants with unascertainable primary endpoint status from the primary analysis population.

**********

what does this mean?). If published, this will include your full peer review and any attached files.

If you choose “no”, your identity will remain anonymous but your review may still be made public.

**Do you want your identity to be public for this peer review?**  For information about this choice, including consent withdrawal, please see our Privacy Policy

Reviewer #5: No

**********

[NOTE: If reviewer comments were submitted as an attachment file, they will be attached to this email and accessible via the submission site. Please log into your account, locate the manuscript record, and check for the action link "View Attachments". If this link does not appear, there are no attachment files.]

To ensure your figures meet our technical requirements, please review our figure guidelines: https://journals.plos.org/plosone/s/figures

You may also use PLOS’s free figure tool, NAAS, to help you prepare publication quality figures: https://journals.plos.org/plosone/s/figures#loc-tools-for-figure-preparation.

NAAS will assess whether your figures meet our technical requirements by comparing each figure against our figure specifications.

# Author comment: Author response to Decision Letter 2

**DOI:** 10.1371/journal.pone.0361468.r004

2 Oct 2026

Dear Editor and Reviewers,

Thank you for your careful and constructive review of our manuscript. We have revised the manuscript in accordance with the comments and believe that the revisions have improved its clarity and scientific rigor.

Attachment Submitted filename: Response_to_Reviewers_auresp_2.docx

# Peer review: Decision Letter 2

**DOI:** 10.1371/journal.pone.0361468.r005

**Authors:** Athanasios G. Pantelis

12 Oct 2026

Prophylactic endoscopic transpapillary gallbladder stenting at fully covered self-expandable metal stent placement to prevent cholecystitis in malignant distal biliary obstruction: A multicenter prospective single-arm study protocol (PROTECT study)

PONE-D-26-28021R2

Dear Dr. Kurita,

We’re pleased to inform you that your manuscript has been judged scientifically suitable for publication and will be formally accepted for publication once it meets all outstanding technical requirements.

Within one week, you’ll receive an e-mail detailing the required amendments. When these have been addressed, you’ll receive a formal acceptance letter and your manuscript will be scheduled for publication.

An invoice will be generated when your article is formally accepted. Please note, if your institution has a publishing partnership with PLOS and your article meets the relevant criteria, all or part of your publication costs will be covered. Please make sure your user information is up-to-date by logging into Editorial Manager at Editorial Manager® and clicking the ‘Update My Information' link at the top of the page. For questions related to billing, please contact billing support.

If your institution or institutions have a press office, please notify them about your upcoming paper to help maximize its impact. If they’ll be preparing press materials, please inform our press team as soon as possible -- no later than 48 hours after receiving the formal acceptance. Your manuscript will remain under strict press embargo until 2 pm Eastern Time on the date of publication. For more information, please contact onepress@plos.org.

Kind regards,

Athanasios G. Pantelis

Academic Editor

PLOS One

Additional Editor Comments (optional):

Reviewers' comments:

Reviewer's Responses to Questions

**Comments to the Author**

1. Does the manuscript provide a valid rationale for the proposed study, with clearly identified and justified research questions?

Reviewer #5: Yes

**********

2. Is the protocol technically sound and planned in a manner that will lead to a meaningful outcome and allow testing the stated hypotheses?

Reviewer #5: Yes

**********

3. Is the methodology feasible and described in sufficient detail to allow the work to be replicable?

Reviewer #5: Yes

**********

4. Have the authors described where all data underlying the findings will be made available when the study is complete?

The PLOS Data policy

Reviewer #5: Yes

**********

5. Is the manuscript presented in an intelligible fashion and written in standard English?

Reviewer #5: Yes

**********

Please use the space provided to explain your answers to the questions above and, if applicable, provide comments about issues authors must address before this protocol can be accepted for publication. You may also include additional comments for the author, including concerns about research or publication ethics.

You may also provide optional suggestions and comments to authors that they might find helpful in planning their study.

Reviewer #5: All statistical comments have been adequately addressed.

**********

what does this mean?). If published, this will include your full peer review and any attached files.

If you choose “no”, your identity will remain anonymous but your review may still be made public.

**Do you want your identity to be public for this peer review?**  For information about this choice, including consent withdrawal, please see our Privacy Policy

Reviewer #5: No

**********

# Editor report: Acceptance letter

**DOI:** 10.1371/journal.pone.0361468.r006

**Authors:** Athanasios G. Pantelis

PONE-D-26-28021R2

PLOS One

Dear Dr. Kurita,

I'm pleased to inform you that your manuscript has been deemed suitable for publication in PLOS One. Congratulations! Your manuscript is now being handed over to our production team.

At this stage, our production department will prepare your paper for publication. This includes ensuring the following:

* All references, tables, and figures are properly cited

* All relevant supporting information is included in the manuscript submission,

* There are no issues that prevent the paper from being properly typeset

You will receive further instructions from the production team, including instructions on how to review your proof when it is ready. Please keep in mind that we are working through a large volume of accepted articles, so please give us a few days to review your paper and let you know the next and final steps.

Lastly, if your institution or institutions have a press office, please let them know about your upcoming paper now to help maximize its impact. If they'll be preparing press materials, please inform our press team within the next 48 hours. Your manuscript will remain under strict press embargo until 2 pm Eastern Time on the date of publication. For more information, please contact onepress@plos.org.

You will receive an invoice from PLOS for your publication fee after your manuscript has reached the completed accept phase. If you receive an email requesting payment before acceptance or for any other service, this may be a phishing scheme. Learn how to identify phishing emails and protect your accounts at https://explore.plos.org/phishing.

If we can help with anything else, please email us at customercare@plos.org.

Thank you for submitting your work to PLOS One and supporting open access.

Kind regards,

PLOS One Editorial Office Staff

on behalf of

Dr. Athanasios G. Pantelis

Academic Editor

PLOS One
